# Novel Association Strategy with Copy Number Variation for Identifying New Risk Loci of Human Diseases

**DOI:** 10.1371/journal.pone.0012185

**Published:** 2010-08-20

**Authors:** Xianfeng Chen, Xinlei Li, Ping Wang, Yang Liu, Zhenguo Zhang, Guoping Zhao, Haiming Xu, Jun Zhu, Xueying Qin, Suchao Chen, Landian Hu, Xiangyin Kong

**Affiliations:** 1 The Key Laboratory of Stem Cell Biology, Institute of Health Sciences, Shanghai Institutes for Biological Sciences (SIBS), Chinese Academy of Sciences (CAS) and Shanghai Jiao Tong University School of Medicine (SJTUSM), Shanghai, People's Republic of China; 2 Chinese National Human Genome Center at Shanghai, Shanghai, People's Republic of China; 3 Institute of Bioinformatics, College of Agriculture and Biotechnology, Zhejiang University, Hangzhou, People's Republic of China; 4 State Key Laboratory of Computer Aided Design and Computer Graphics, Zhejiang University, Hangzhou, People's Republic of China; 5 State Key Laboratory of Medical Genomics, Ruijin Hospital, Shanghai Jiaotong University, Shanghai, People's Republic of China; University of Montreal, Canada

## Abstract

**Background:**

Copy number variations (CNV) are important causal genetic variations for human disease; however, the lack of a statistical model has impeded the systematic testing of CNVs associated with disease in large-scale cohort.

**Methodology/Principal Findings:**

Here, we developed a novel integrated strategy to test CNV-association in genome-wide case-control studies. We converted the single-nucleotide polymorphism (SNP) signal to copy number states using a well-trained hidden Markov model. We mapped the susceptible CNV-loci through SNP site-specific testing to cope with the physiological complexity of CNVs. We also ensured the credibility of the associated CNVs through further window-based CNV-pattern clustering. Genome-wide data with seven diseases were used to test our strategy and, in total, we identified 36 new susceptible loci that are associated with CNVs for the seven diseases: 5 with bipolar disorder, 4 with coronary artery disease, 1 with Crohn's disease, 7 with hypertension, 9 with rheumatoid arthritis, 7 with type 1 diabetes and 3 with type 2 diabetes. Fifteen of these identified loci were validated through genotype-association and physiological function from previous studies, which provide further confidence for our results. Notably, the genes associated with bipolar disorder converged in the phosphoinositide/calcium signaling, a well-known affected pathway in bipolar disorder, which further supports that CNVs have impact on bipolar disorder.

**Conclusions/Significance:**

Our results demonstrated the effectiveness and robustness of our CNV-association analysis and provided an alternative avenue for discovering new associated loci of human diseases.

## Introduction

Copy number variations (CNV) are DNA segments with gains or losses in copy number longer than 1 kb compared to a reference genome. At least 12% of the human genome has been identified as copy number variable [1] and expression correlation studies have revealed that these pervasive CNVs may affect physiological function through regulating gene expression [2]. Increasing evidence has shown that CNVs play important causal roles in human diseases. For example, *CCL3L1*-related segmental duplication influences susceptibility to HIV-1/AIDS [3]. CNVs at 1q21.1 were associated with neuroblastoma [4]. What is more, *de novo* CNVs have been associated with autism [5] and sporadic schizophrenia [6].

The increasing functions found for CNVs in human diseases make a genome-wide systematic survey of CNVs become intriguing. However, there are no such effective tools for testing the association of CNVs with disease in genome-wide scale. Although many challenges have been reviewed previously [7], [8], here we re-emphasize the challenges in establishing such statistical model. First, CNVs have a genomic localization pattern that spans thousands of nucleotides, thus comparison among a chromosomal region may be more effective than the specific testing on a designated nucleotide. However, it is hard to pre-define the range required for comparison in a large case-control dataset, since the range is closely related to the function of the DNA segment. Second, traditional association methods mostly summarized single-nucleotide polymorphism (SNP) allele intensity into raw copy number signal. However, the multiple Gauss-like distribution of the raw copy number signal among cases and controls, which has been recognized in other studies [1], [7], [8] and in our data manipulation, demands a complicated statistical model with multiple assumptions to analyze CNV-association. These assumptions will not be suitable for every test, and parameters for these assumptions that are inferred from experience will not be applicable to every analysis. Those two challenges together with the complexities that originate from signal noise, summarization declination (which occurs when combining the SNP's alleles with non-linear measurements) and batch bias (which is from unequal influence of individual selection and experimental condition), make it difficult to analyze CNV-association in a uniform one-step test. Additionally, one-step CNV-association testing, especially through complicated signal summarization, causes intractable results that cannot be compared to the primary data, whereas verification of association results from primary data is important in genome-wide association studies to provide the confidence for the findings by common consent.

**Figure 1:**
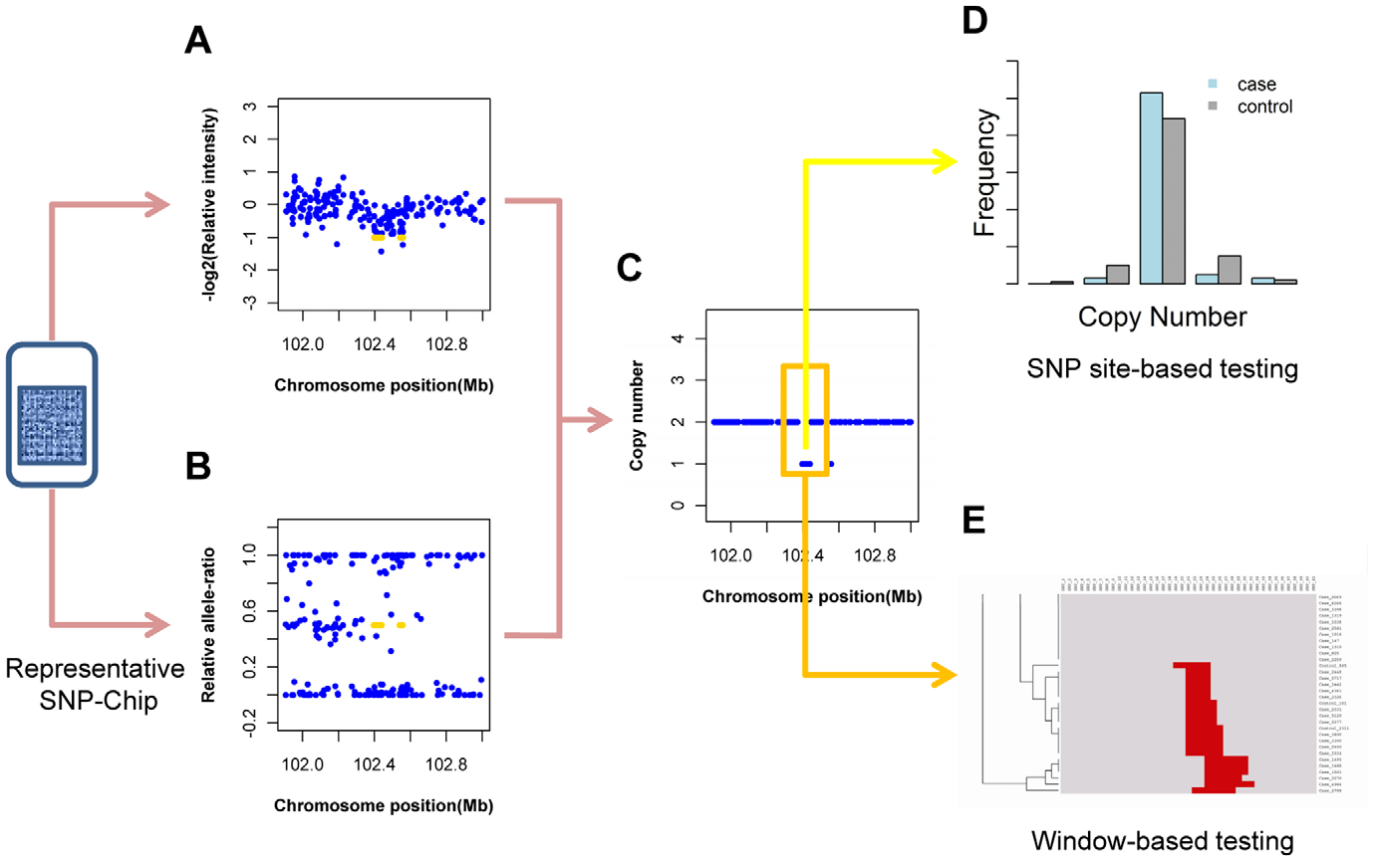
CNV-association strategy transforms raw signal into copy number and detects association through site-specific testing and CNV-pattern clustering. (**A**) Relative intensity was log2-transformed value for the normalized intensity-sum of the SNP alleles. (**B**) the relative allele-ratio was actually a normalized anti-tangent value for the intensity ratio of SNP alleles. These two measurements were arranged along the chromosomal sequence as a hidden Markov model. (**C**) In this model (with well-trained parameters), the copy number could be calculated from the measurements on each SNP site and the neighboring copy numbers. (**D**) The copy numbers of a designated site for cases and controls were classified before performing the SNP site-based testing, a Chi-squared test with triple NULL hypotheses in which deletion (labeled as **Loss**), amplification (labeled as **Gain**) or both (labeled as **Abnm**) were viewed as abnormal. Copy numbers in a window centered to the significant SNP site (denoted in the orange box) were subjected to a complete linkage clustering (**E**). To this clustering heat map, a statistical test on the CNV-pattern (named as window-based testing) was used to reconfirm the significance of association. (See details in the **Materials and Methods**.)

Here, we developed an integrated strategy to test CNV-association with disease in large-scale case-control studies in which single-nucleotide polymorphisms were used to calculate the copy numbers. Our strategy processed the data in a hierarchical mode to address the challenges individually (schematically illustrated in **Figure 1** and detailed in **Materials and Methods**). We transformed continuous signal into discrete copy number to eliminate signal noise and slight batch bias. We performed a SNP specific testing with triple null hypotheses (named as **SNP site-based testing**) to conform to pathophysiologically functioning way of CNV. We also conducted a geographical pattern-comparison of CNV (named as **window-based testing**) to ensure confidence. We applied this strategy to genome-wide data with seven common diseases from the Wellcome Trust Case-Control Consortium (WTCCC) [9]. In the original paper of these data, they found 24 independent associated signals and several moderate significant signals through genotype-association testing that was mostly applied in classical genome-wide association study. By using SNP site-based testing and further window-based testing, we identified 36 new susceptible loci for these seven diseases, and none of which were reported previously to be affected by CNVs. Through data querying for physiological mechanisms and genotype-association, 15 of these identified loci were reported to be relevant with those diseases, which indicate that our results are valuable for further disease-related studies.

## Results

Two features of our CNV-association strategy were proved effective in the following results. The first is the multiple hypotheses for the functioning mode of CNVs in disease. CNVs may affect phenotype through regulating gene's expression, such as the common CNV-loci for the gene *PDPR* [8], while most CNVs' deletion and amplification may not be consistent to the genes' down-regulation and over-expression. The most important reason is that most CNVs do not embrace the whole DNA segments of one gene, but just located or truncated in the coding, intron, enhancer or insulator region. Deletion and amplification in these CNVs may just prohibit the gene's expression or disable the gene's function with aberrant isoforms. Thus, the complex roles of deletion and amplification should be carefully considered in the association testing. Here, we hypothesized that deletion and/or amplification contribute to the gene function separately to cope with its complexity. CNVs may also affect diseases through their distribution tendency between cases and controls; therefore, three kinds of trend tests were applied to test the CNVs' association with disease (**Text S1**). The second feature is comparison of the CNV's geographical pattern between cases and controls. Our method applied a window-based chromosome-along clustering algorithm to the candidate CNV-loci, and then tested these clustering results statistically. The accidental signal noise could be easily excluded through the clustering procedure, and the credible CNVs from common ancestry or with identical physiological significance tend to congregate around nearby nodes in the clustering heat map. The statistical testing on these congregated CNVs is more accurate than SNP site-based testing, and provides further confidence to our results.

How to confirm the significance of multiple tests for CNV-association is another focus of our strategy. The CNV-association *P* values are different from common *P* values in multiple tests, in that these *P* values tend to be related with the neighboring sites, which is a phenomenon caused by the geographical stretch of CNVs. Thus, the classical Bonferroni correction is not suitable. A permutation method based on the actual data was required to generate the theoretical distribution of multiple *P* values. Here, we hypothesized that the case-control effect size for every loci is null, and the difference of measurements originate from individual selection. We permuted the labels of cases and controls, recalculated the association *P* values, and then computed the false discovery rate (FDR) to educe the appropriate significance level for the association results (see details in **Materials and Methods**).

### Genome-wide CNV-association results

**Figure 2:**
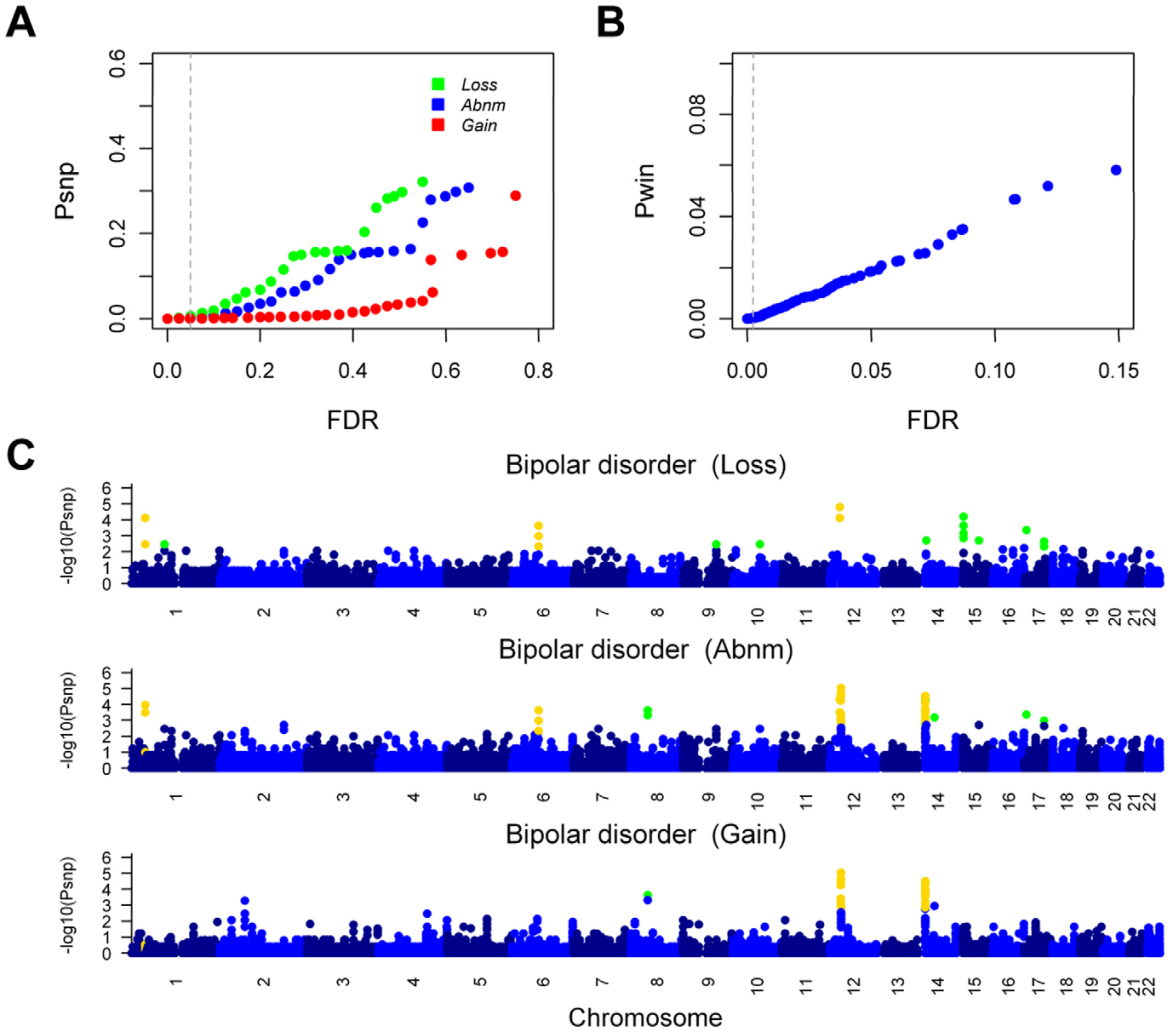
Thresholds for the significance of CNV-association and genome-wide distribution of the results in bipolar disorder. (**A**) In the SNP site-based testing, 1000 permutations were performed and the boundary *P* values (*Psnp*) were plotted against the false discovery rate (FDR) values, with different colors indicating the different hypotheses (blue for **Abnm**, green for **Loss** and red for **Gain**). FDR<0.05 (labeled with vertical dashed line) for each hypothesis was used to select 2488 SNPs as candidates for the window-based testing. (**B**) In the window-based testing, 25000 permutations were performed and the resulting *P* values (*Pwin*) were plotted against the FDR values. 401 SNP sites were selected as the final results, with an FDR of 2.35×10^−3^ (indicated by the vertical dashed line) to ensure that the false positives in all the results were less than 1. (**C**) The −log10 of the SNP site-based *P* values were plotted against the position on each chromosome. The three hypotheses are plotted in different panels, and the *P* values of the chromosomes are shown in alternating colors for clarity. The *P* values that passed the SNP site-based testing are highlighted in green, and the *P* values that passed the window-based testing are highlighted in yellow. The genome-wide distribution results for the seven diseases are in **Figure S1**.

When the FDR was set to less than 0.05 for each hypothesis in the SNP site-based testing, 2488 SNP sites with *P* values above the significance level were obtained for further window-based testing (**Figure 2A**). With an FDR of 2.35×10^−3^ for the window-based testing, we identified 401 disease susceptible SNP sites as disease susceptible, in which 219 SNP sites were non-redundant (**Figure 2B**, **Table S1**): 43 are associated with bipolar disorder (BD; MIM 125480), 17 with coronary artery disease (CAD; MIM 607339), 5 with Crohn's disease (CD; MIM 266600), 41 with hypertension (HT; MIM 145500), 61 with rheumatoid arthritis (RA; MIM 180300), 37 with type 1 diabetes (T1D; MIM 222100) and 15 with type 2 diabetes (T2D; MIM 125853). Thirty-six CNV-loci were identified from these SNP sites through combining neighboring SNP sites (**Table 1**).

**Table 1:** CNV-loci associated with seven diseases.

Disease	Chromosome	Landmark	Loss	Abnm	Gain	Window-based	Trend	Genotypic
BD	1p34.3	rs16824514	7.55×10^−5^	1.06×10^−4^	3.83×10^−1^	1.16×10^−5^	1.58×10^−1^	1.42×10^−1^
BD	6q13	rs4991400	2.29×10^−4^	2.29×10^−4^	1.00	2.29×10^−4^	6.73×10^−1^	8.78×10^−1^
BD	12p11.22	rs10843150	1.60×10^−5^	4.90×10^−5^	1.00	1.60×10^−5^	8.26×10^−1^	9.59×10^−1^
BD	12p11.21	rs4931443	1.00	2.98×10^−5^	2.98×10^−5^	1.36×10^−5^	NA	NA
BD	14q11.2	rs2635556	3.34×10^−1^	7.55×10^−5^	1.36×10^−4^	1.58×10^−5^	NA	NA
CAD	3p26.1	rs163968	2.01×10^−4^	2.01×10^−4^	1.00	2.01×10^−4^	9.16×10^−1^	8.46×10^−1^
CAD	7q21.11	rs10245061	4.85×10^−8^	4.85×10^−8^	1.00	4.85×10^−8^	2.62×10^−1^	2.10×10^−1^
CAD	16q22.1	rs2303200	1.64×10^−2^	3.93×10^−4^	8.98×10^−7^	8.98×10^−7^	NA	NA
CAD	19q13.2	rs2016070	1.51×10^−1^	9.54×10^−3^	1.39×10^−5^	1.39×10^−5^	NA	NA
CD	19q13.2	rs1015758	2.31×10^−1^	1.94×10^−3^	1.00×10^−4^	4.88×10^−5^	NA	NA
HT	1p31.1	rs596204	2.85×10^−5^	2.85×10^−5^	1.00	2.85×10^−5^	2.02×10^−1^	6.05×10^−2^
HT	2q13	rs3906021	1.52×10^−4^	2.03×10^−4^	1.00	5.36×10^−5^	9.52×10^−1^	5.78×10^−1^
HT	5q12.1	rs4302532	2.14×10^−4^	2.14×10^−4^	1.00	2.14×10^−4^	6.65×10^−1^	6.79×10^−1^
HT	5q22.1	rs152875	5.08×10^−5^	2.65×10^−5^	5.20×10^−1^	8.98×10^−5^	NA	NA
HT	10p14	rs263431	2.21×10^−4^	2.21×10^−4^	1.00	1.67×10^−4^	1.00	1.00
HT	10q25.3	rs2419854	1.34×10^−4^	1.34×10^−4^	1.00	1.69×10^−4^	2.70×10^−1^	5.33×10^−1^
HT	11q12.2	rs175126	2.26×10^−3^	2.26×10^−3^	1.00	5.20×10^−5^	2.70×10^−1^	4.24×10^−1^
RA	1q23.3	rs10917851	1.95×10^−4^	7.55×10^−5^	3.88×10^−1^	1.95×10^−4^	1.94×10^−1^	4.28×10^−1^
RA	2q31.2	rs2303836	7.55×10^−5^	7.55×10^−5^	1.00	7.55×10^−5^	5.91×10^−2^	6.26×10^−3^
RA	7p21.3	rs1467345	1.31×10^−3^	1.31×10^−3^	1.00	6.14×10^−6^	6.68×10^−1^	9.12×10^−1^
RA	7q21.11	rs10245061	8.20×10^−8^	8.20×10^−8^	1.00	8.20×10^−8^	8.04×10^−1^	4.09×10^−1^
RA	8p11.1	rs12550215	2.92×10^−5^	1.40×10^−5^	3.06×10^−1^	2.92×10^−5^	9.92×10^−1^	1.01×10^−1^
RA	9p23	rs10977624	2.92×10^−5^	2.92×10^−5^	1.00	1.95×10^−4^	1.86×10^−1^	3.96×10^−1^
RA	15q13.3	rs2926504	8.46×10^−7^	1.07×10^−5^	4.11×10^−1^	8.46×10^−7^	2.40×10^−1^	2.64×10^−1^
RA	16q22.1	rs2303200	2.75×10^−1^	1.18×10^−3^	1.05×10^−4^	6.67×10^−5^	NA	NA
RA	19q13.2	rs2016070	9.17×10^−1^	1.23×10^−4^	6.19×10^−6^	6.19×10^−6^	NA	NA
T1D	1p36.13	rs6429757	8.75×10^−6^	6.20×10^−6^	1.00	8.75×10^−6^	4.43×10^−1^	6.86×10^−1^
T1D	1q41	rs337147	9.51×10^−7^	8.85×10^−5^	1.00	9.51×10^−7^	5.66×10^−1^	6.62×10^−1^
T1D	2p14	rs13409606	1.06×10^−5^	1.06×10^−5^	1.00	1.06×10^−5^	NA	NA
T1D	5q22.1	rs524203	5.68×10^−4^	3.11×10^−4^	5.20×10^−1^	1.58×10^−4^	8.76×10^−1^	9.77×10^−1^
T1D	10p15.3	rs2210553	1.00×10^−5^	1.00×10^−5^	1.00	1.00×10^−5^	5.49×10^−1^	3.16×10^−1^
T1D	14q11.2	rs10873018	1.70×10^−7^	1.20×10^−7^	1.00	1.70×10^−7^	6.26×10^−3^	2.00×10^−3^
T1D	15q11.2	rs2880332	8.88×10^−6^	3.29×10^−1^	4.74×10^−2^	1.13×10^−4^	NA	NA
T2D	1p34.3	rs16824514	1.52×10^−3^	1.64×10^−2^	5.22×10^−1^	2.35×10^−4^	6.35×10^−1^	8.86×10^−1^
T2D	1q41	rs337147	3.65×10^−6^	1.17×10^−3^	4.13×10^−1^	3.65×10^−6^	9.61×10^−1^	8.03×10^−1^
T2D	19q13.2	rs2016070	5.79×10^−5^	3.64×10^−6^	4.44×10^−3^	5.79×10^−5^	NA	NA

**Notes: Landmark** is the representative SNP site in the associated CNV-loci. **Loss**, **Abnm** and **Gain** denote the three hypotheses in the SNP site-based testing, in which deletion, amplification and both were tested respectively; **Window-based** is the *P* values from the window-based testing; **Trend** and **Genotypic** indicate the genotype-association *P* values in the WTCCC paper, and missing *P* values in both of the tests are labeled with “**NA**” for Not Available. A detailled list of SNP sites that were associated with diseases could be found in the **Table S1**. Seven diseases were tested with CNV-association in the present work, which are bipolar disorder (**BD**), coronary artery disease (**CAD**), Crohn's disease (**CD**), hypertension (**HT**), rheumatoid arthritis (**RA**), type 1 diabetes (**T1D**) and type 2 diabetes (**T2D**).

We found that SNP sites in close proximity to one another tended to have similar levels of significance (**Figure 2C**, **Figure S1**). These results indicate that these SNP sites belong to the same copy number variable region and that these convergent associations are unlikely to be random events. The high quality of the final clustering heat map provides confidence for these susceptible CNV-loci, and the relatively clean CNV-pattern boundaries in the clustering heat map also indicates the high credibility of these CNVs (**Figure S4**). Some of the susceptible CNV-loci in our study are associated with multiple diseases (**Table 1**), which is consistent with the results obtained in the WTCCC genotype-association study (such as that rs6679677 is significant in RA and T1D) [9].

### Biological relevance of the risk loci

To further confirm the effectiveness of our strategy, we performed data mining within a 0.2-Mb region (which is an empirical estimation that regulatory elements have a median distance of 0.1 Mb away from the coding sequence [10], [11]) around the centered significant SNP sites for their functional relationships with corresponding diseases from previous publications. Fifteen CNV-loci were previously reported to be functionally related to or be associated with the investigated diseases (**Table S2**). For example, CEACAMs (Carcinoembryonic antigen-related cell adhesion molecule 4, 7, 21) are matrix molecules localized at the apical glycocalyx of normal colonic epithelium. They are bacteria receptors [12], [13] and have multiple roles in the pathogenesis of Crohn's disease [14], [15]. Another example is *CASP9* (MIM, 602234), which participates the immune attack in a murine model of type 1 diabetes [16].

Obvious physiological relevance of these results was observed in the susceptible genes identified for bipolar disorder, including *INPP5B* (MIM, 147264), *POU3F1* (MIM, 602479), *MTF1* (MIM, 600172), *CCDC91* (coiled-coil domain containing 91), *KCNQ5* (MIM 607357) and Olfactory receptors (*OR4K5*, *OR4K2*, *OR4M1*, *OR4K1*, *OR4N2*, *OR4K14*, *OR4K13*; 14q11.2). *INPP5B* hydrolyzes the calcium-mobilizing second messenger inositol 1,4,5-trisphosphate (IP_3_), which is a signal-terminating reaction in the calcium/IP_3_ pathway and may directly affect neurophysiologic regulation [17]. In this locus, deletion is prevalent in the cases (**Figure S4A**), which make sense that deletion might down-regulate the *INPP5B* expression, leading to inability of terminating the excited calcium flux. *POU3F1*, also known as Oct6, plays a crucial role in neurodevelopment and has been shown to be potentially relevant in schizophrenia [18]. *POU3F1* was also proved to affect calcium flux through binding to the promoter region of *PIK3C3* (MIM 602609), a member of the phosphatidylinositide 3-kinase family, and mutations in *PIK3C3* have been shown to be involved in a subset bipolar disorder and schizophrenia patients [19]. *MTF1* binds to the metal responsive element, which is regulated by lithium salts in the treatment of bipolar disorder [20] and may also interact with cytosolic calcium [21]. More than 1% of the cases could be confirmed with deletion in 12p11.22 (**Figure S4C**). The gene *CCDC91* in this locus may affect protein sorting and membrane trafficking through interacting with GGAs (Golgi-localized, Gamma ear-containing, ARF-binding proteins) [22]. It has been found that many members in the same pathway of *CCDC91* are associated with bipolar disorder, and its binding partner AP1G1 was up-regulated in the post-mortem cerebellum of schizophrenia patients [23], [24]. *KCNQ5* (potassium voltage-gated channel subfamily KQT member 5) may contribute to episodic disturbances of mood and behavior as well-characterized roles in other ion-channelopathies [25], and two family members, *KCNC2* and *KCNQ2*, were found to be associated with bipolar disorder [9], [26]. What is more, *KCNQ5* is connected to phosphoinositide signaling through regulation by *PIP5K2A*(MIM 603140), a schizophrenia-associated gene [27]. Olfactory receptors, which belong to the G-protein coupled receptor 1 family, may play some roles in intracellular aberrant calcium mobilization of olfactory neurons in bipolar patients [28]. The CNV-association significances of those seven olfactory receptors also match the nearby genotype association (rs7159947, trend *P* value = 4.91×10^−4^, genotypic *P* value = 2.02×10^−3^) in the WTCCC paper [9]. Moreover, the CNV differentiation of those olfactory receptors can also be used to explain the substantial olfactory deficits in patients with schizophrenia [29], a psychotic disorder related to bipolar disorder.

Therefore, all those associated genes listed above are related to phosphoinositide/calcium pathway (whose interaction-relationship is illustrated in **Figure S5**), which strongly suggests the role of this pathway in bipolar disorder. Moreover, detailed analysis of these associated genes (**Figure S4A, B, C**) implicated that CNVs with similar boundary and pattern might play roles in the inheritance of bipolar disorder.

### Our strategy found disease-associated CNV-loci from the SNP sites that were omitted by traditional genotype-association analysis

CNVs are different genetic variations from SNPs, in that they are deletion or amplification of DNA fragments but not single-nucleotide polymorphisms. Here, we compared our CNV-association results with SNP genotype-association in WTCCC. There was little tendency-accordance between the both results on SNP site level (**Figure S2**), which reflects that CNV-association are unique from genotype-association and could be new way in discovering associated loci with human disease. In comparison of the associated genes, we found that potassium voltage-gated channel subfamily (*KCNQ5* in CNV-association and *KCNC2* in genotype-association), olfactory receptors could be found in the both results.

**Figure 3:**
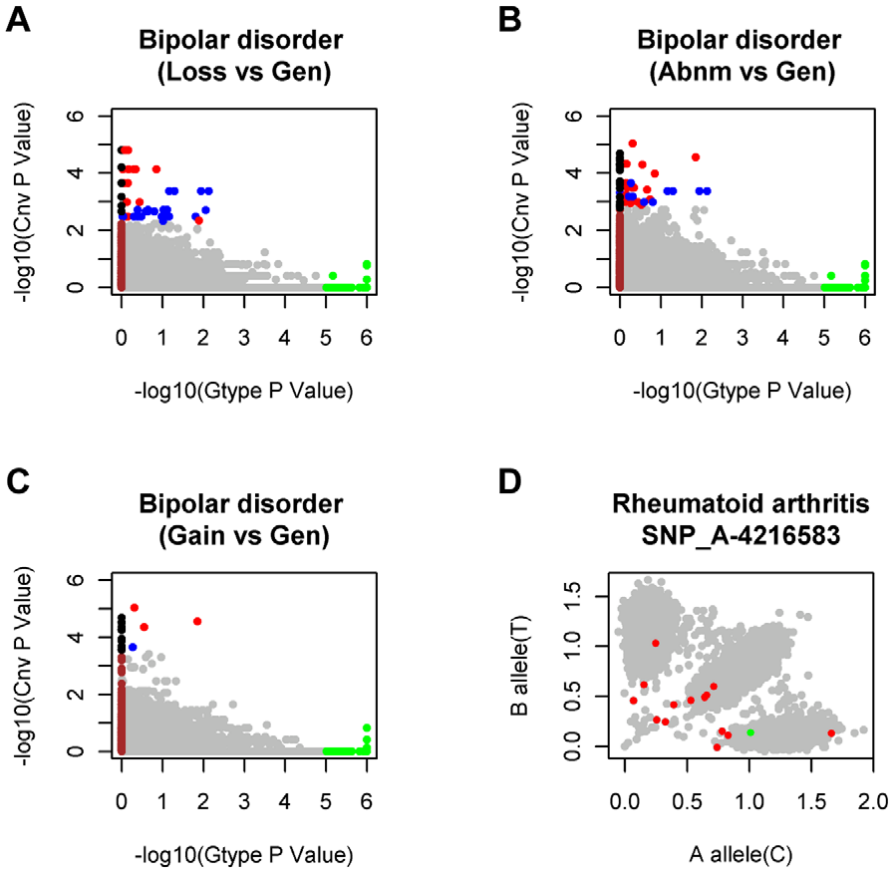
Comparison with the traditional genotype-association analysis demonstrates the priority of our method in CNV-regions. “**Gen**” labels the genotypic testing (a Chi-squared test with 2 degrees of freedom) results obtained from the WTCCC paper [9]. The −log10 of SNP site-based *P* values in our study with the triple NULL hypotheses, in which deletion (**A**, labeled **Loss**), amplification (**C**, labeled **Gain**) and both (**B**, labeled **Abnm**) were evaluated separately, are plotted against the −log10 of the *P* value from the genotype-association test of WTCCC [9]. For clarity, the genotype-association *P* values<10^−5^ are highlighted in green, the CNV-association *P* values that passed the single SNP site-based testing are in blue, and the CNV-association *P* values that passed the window-based testing are in red. The SNP sites that are absent from the genotype-association testing are plotted by default as zero (highlighted in brown), and the absent sites that passed the SNP site-based testing are labeled with black. The genotypic testing (**Gen**) and trend testing (**Add**, another testing for genotype tendency of disease in WTCCC [9]) for the seven disease are compared with our CNV-association results in **Figure S2**. (**D**) Evidence that CNVs can lead to chaotic genotyping clusters in copy number variable regions. All the 17000 individuals are labeled with grey, individuals with CNVs in the disease group are in red, and individuals with CNVs in controls are in green. More evidence of chaotic sample-wide intensity maps affected by CNVs can be found in **Figure S3**.

One interesting finding is that a large proportion (25.1%) of our results were absent in genotype-association analysis (**Figure 3A, B, C**, **Figure S2** and **Table S1**). This phenomenon results from two limitations of genotype association: the genotyping model in the association test is limited to three genotypes (AA, BB, AB) and the genotyping quality is dependent on the sample-wide intensity mapping of the A and B alleles. Both of these limitations are not suitable for SNPs in copy number variable regions with chaotic intensity mapping and with genotypes not limited to three (shown by the sample-wide intensity maps in **Figure 3D** and **Figure S3**). Similar phenomenon of CNVs' effect on genotype-association has been found in the locus of *CYP2D6* in breast cancer [30], and it has been pointed out that CNVs in this locus should be assessed before genotype-association analysis [31].

## Discussion

In the present study, we have shown that our two-level CNV-association testing is a reliable strategy to search for risk CNV-loci. This method is complement to the traditional genotype-association analysis, and provides a unique way to discover new causes for human diseases. Our results provide further evidence that CNVs involve in many common diseases. In addition, the new risk CNV-loci identified in our work will be helpful on understanding the pathogenesis of these diseases.

The functions of CNVs are interesting topics in disease-risk study, the complexity of which has been mentioned previously [1], [8], but they are far from being fully understood. Linear-effect model is the most common hypothesis on how CNVs function in disease, and the findings in the CNV-loci 16q22.1 (Armitage trend test *P* value = 3.98×10^−8^, the trend also shows in clustering heat map) can support this hypothesis: amplification of two genes (*PDPR* and *AARS*) in 16q22.1 might impose a counteractive effect on CAD, while loss of copy number may cause weaker recovery from heart attack as they function in post-ischemic heart [32], [33]. However, most loci have more complicated association with disease. Loss of copy number in the gene *INPP5B* may induce leakage-cleanup of IP_3_ in the nerve conduction, and then induce patients to lose control of excited emotion, while gain of copy number shows no obviously effect in bipolar disorder. In 14q11.2, a common copy number variable region, copy number differentiation may produce irregular isoforms of olfactory receptors, and then causes mis-perception in the olfactory and neuronal cells. Pattern discordance could be found in many CNV-loci of RA and T1D, which might be from aberration of DNA recombination and repair in somatic and germ cells. The CNVs with pattern discordance may make an impact on these diseases by interfering the genes' functions explicitly or through cell-recognition in complicated immune pathway. In a word, copy number variations may work in very complex way in the development of disease, and the function of risk CNV-loci need a locus-by-locus analysis.

## Materials and Methods

### Disease data and training set

Two Affymetrix Mapping 500K chip datasets were used in the present work, which include the Wellcome Trust Case-Control Study Consortium (WTCCC) data for seven diseases (each comprising 2000 cases and 3000 normal control individuals) [9] and a training data of 90 individuals in Utah, USA, from the Centre d'Etude du Polymorphisme Humain collection (abbreviated as CEU, which is of Northern and Western European ancestry) [34]. The WTCCC data was used to test the association of the CNVs with disease. Individuals from the WTCCC data were filtered following the instructions in the paper [9] in order to avoid contamination, false positives, non-Caucasian ancestry and relatedness. Based on the multi-scale comparison between the CEU data and WTCCC data in the original paper [9], the CEU data was selected as the training set to estimate the parameters of the hidden Markov model. The CEU data was quantile normalized to the WTCCC data as described in the WTCCC paper [9].

We processed the data using SNPs annotated in the NCBI build 35 and build 36 reference databases, and the results in the different builds were almost the same. In the main text and supporting information, results were only shown for build 35. Since the sex chromosomes are different from autosomes in copy number detection and comparison, only the autosomes were tested in our work.

### Strategy of CNV-association with one-step data transformation and two-pass statistical testing

CNVs are mostly detected from SNP genotyping data, thus a precise transformation from SNP allele intensity into copy number is essential in testing the association between CNVs and diseases. In our work, a powerful hidden Markov model that makes the best of the SNP allele information was applied. To make sure that the parameters of hidden Markov model were suitable for all the high-throughput dataset of WTCCC, we developed a training program to calculate these parameters. This hidden markov model is depicted in the **Figure 1A, B, C**, and is detailed in the following text.

The significance of the CNVs was evaluated in two levels of statistical tests: (i) **SNP site-based testing** to measure the disease-association on a specific SNP site and (ii) **Window-based testing** to measure the CNV-pattern differentiation in and around the selected SNP site. Additionally, **multiple trend testing** was also applied for exploring CNV-association with disease (see details in **Text S1**). The SNP site-based testing was used to selected candidate for window-based testing, and the window-based testing ensured the credibility of the identified CNV-loci. This statistical flow chart is shown in **Figure 1C, D, E** and is detailed in the following text.

### Transformation of SNP signal to copy number

The SNP data from the genotyping chips was first converted to copy number (hidden copy number state) using a well-trained hidden Markov model. The hidden Markov model treated the series of SNP sites, which were arranged along the chromosome sequence, as a hidden Markov chain. The copy number calculation on the site of the hidden Markov chain is dependent on the signal of each SNP site and the copy number of neighboring SNP site. Two measurements for each SNP site were used to calculate its copy number: (i) the Log R Ratio (LRR), which is the log2-transformed value for the normalized intensity-sum of the SNP alleles, and (ii) the B allele frequency (BAF), which actually is a normalized anti-tangent value for intensity-ratio of the SNP alleles. The expected value for each SNP genotype cluster and intensity-sum, which were used in the normalization for LRR and BAF, were the 90% trimmed mean of all of the corresponding values from the WTCCC samples. All of the measurements and transformations above were calculated according to the instructions from the original technical paper [35], but the copy number used in the following case-control association analysis was the total copy number integer from the hidden Markov state in the original technical paper [35]. For example on a certain SNP site, the CNV genotype is “AAB”, which had a copy number of 3 and belonged to state **5** in the original paper; but here we use **3** to denote the CNV genotype.

The hidden Markov model needs a series of parameters to calculate the hidden Markov state, including the LRR's expectation (and standard deviation) for each state, the BAF's expectation (and standard deviation) for each state, the transition probability matrix for inferring relationship among SNP-series, and other parameters. These parameters were trained from the CEU data of HapMap and the program was performed automatically to eliminate bias from manual selection in the original technical paper [35]. Before estimation, hidden states of the training set were assigned with initial values of these parameters, and CNVs that spanned at least three continuous SNP sites were treated as real variations to eliminate coincidence from noise. The LRR's expectation for each state was determined by linear extrapolation. The LRR's standard deviation calculated from SNP sites with two copies was adopted for all other states, as we assumed that noise plays similar roles in every state and this could be seen in the actual data distribution. The BAF's expectation and standard deviation for every state were inferred from the data distribution of the previous state calling. Transition probability for the hidden Markov model was calculated using the Baum-Welch algorithm, and a chromosome-weighted mean was used as the actual transition probability. Repetition of the estimation program was performed using the newly generated values of these parameters, until constant values were obtained independently of the initial values, with a change of <0.0001 for every parameter.

### SNP site-based testing for site-specific significance

The influence of changes in copy number (amplification and/or deletion) on physiological function is far from fully understood, so testings for amplification, deletion or the both were used to measure the significance on single SNP site. We postulated triple NULL hypotheses that (i) amplification (denoted as 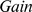), (ii) deletion (denoted as 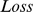) or (iii) the both (denoted as 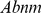) show no difference between the case and control groups. For a certain SNP site, the copy numbers of cases and controls could be summarized in **Table 2**.

**Table 2:** The numbers of cases and controls in SNP site-based testing.

		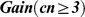
		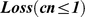
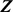	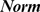	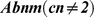
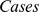	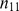	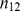
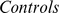	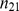	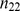

**Notes:** 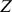 is the number of samples, 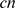 is the copy number of individuals. 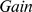, 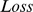 and 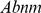 denote the three hypotheses, in which amplification, deletion and the both were tested separately. 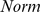 is the number of the samples that exclude those with CNVs tested. The element of this table, 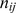, is the number of individuals in the different conditions.

If N denotes the total number of cases and controls, 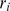 denotes the sum of the rows and 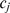 denotes the sum of columns, the expected value for 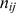 is calculated from 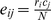. The *P* value could be calculated from the following chi-squared distribution with one degree of freedom:
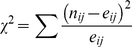
If any of the 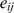 values were less than 5, a Fisher's exact test was applied instead. If 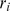 and 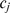 are constant in the permuted contingency table and 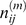 is number of the elements in permutation, the exact *P* value for one permutation can be calculated as follows:
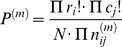
Then the Fisher's exact *P* value is calculated as follows:
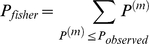



### Window-based testing for CNV-patterns

Window-based testing was based on clustering heat map of CNV-pattern, which can test the CNV-association in and around the specific SNP site. In this testing, copy number, which was generated from random noise or possessed irregular pattern, could be eliminated. For an individual, a series of SNP sites in a window centered on a specific site were extracted to measure the CNV-region. This region can be described by vectors, such as that 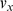 and 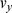 denote vectors from different individuals.
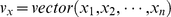


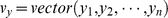
In vectors 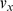 and 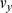, 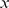 and 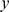 are used to denote the copy numbers for the series of SNP sites in the selected region. 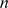 is the dimension of the vector, which is the same as the window size. Based on the distribution of the lengths of CNVs along chromosomes in the actual data, 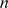 was set to 41 in the present work to bracket the most CNVs in the corresponding region. The Euclidean distance between 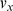 and 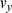 was adopted in the clustering method, and a complete linkage clustering algorithm [36] was modified slightly to accelerate the computation speed in our work. The distance calculation and clustering method above were selected through visual analysis using some example data.

All the vectors clustered in an unbiased manner between the cases and controls. If 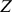 denotes the number of samples, 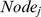 denotes a certain node in the clustering relationship tree and 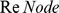 denotes the remaining samples apart from those in 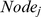. The data obtained for every node could be summarized into contingency **Table 3**.

**Table 3:** The numbers of cases and controls in window-based testing.

*Z*	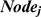	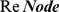
*Cases*	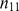	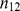
*Controls*	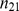	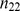

**Notes:** CNV-pattern is classified in the window-based chromosome-along clustering process and is organized in different nodes of the clustering heat map. 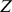 is the number of samples, and 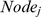 denotes the number of cases or controls in the specific node in the clustering heat map. 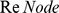 denotes the number of samples that exclude those in the 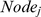. The element of this table, 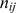, is the number of individuals in the different conditions.

A Chi-squared test could be applied to this table, but when 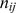 were less than 5, the Fisher's exact test was used instead. The *P* value could be calculated in the same way for the contingency table in SNP site-based testing.

For every node in the clustering tree, a corresponding *P* value could be calculated. The node for the most significant *P* value was defined as “First class node”, and then the corresponding *P* value was named as “First class *P* value,” which was also defined as the *P* value for the window-based testing.
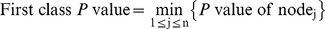
The First class *P* value, which is the most significant using different divisions in the vector dimensions, is the only reasonable measurement to scale the CNV differentiation between cases and controls, since we cannot predefine the actual dimensional division.

### Correction of multiple tests by calculating false discover rates (FDR)

A permutation-based method was used to obtain the significance level for the SNP site-based testing. In the permutation procedure, the labels of the cases and controls were randomly permuted 1000 times, and then the *P* values for all of the diseases were pooled together to calculate the FDR. 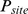 denotes a designated *P* value in the observed data, 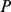 and 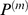 denote the *P* values in the observed data and permutated data respectively, 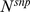 denotes the number of SNP sites and 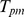 denotes the number of permutations. The FDR for the SNP site-based testing can be calculated using the following formula.
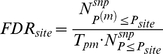
The *P* value in the window-based testing was not only dependent on the neighboring SNP sites but also built upon the clustering structure. To filter out the false positives obtained in multiple tests, we assumed that the copy number windows (which were labeled with the centered SNP sites) possess similar statistical power in all the diseases and all the window-based *P* values were pooled to calculate the FDR. 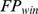 denotes a designated *P* value in the observed data, 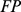 and 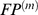 denote the *P* values in the observed data and permuted data respectively, 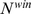 denotes the number of copy number window and 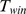 denotes the number of permutations. After the labels for the case and control were permuted 25000 times, the FDR for the window-based testing was calculated as follows:
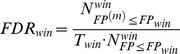
Multiplying the SNP sites number (above boundary *P* value, in the observed data) by the corresponding FDR could provide the estimation of false positives from random coincidence of individual selection. In the SNP site-based testing, FDR<0.05 was used to determine the proper number of candidates for the window-based testing. In the window-based testing, the boundary of the FDR was adjusted to ensure that false positives in the final results were less than 1 copy number window (centered SNP site).

### Supporting Information

Supporting information include supporting data with five figures and two tables, and supporting methods in Text S1. The code for the data transformation and the two-level CNV-association tests was written in the C/C++ programming language. This code and the manual for the data processing are available at http://www.ihs.ac.cn/xykong/CNV_Association_Test.rar.

## Supporting Information

Figure S1 The genome-wide distribution of the CNV-association results in the seven diseases. The −log10 of the SNP site-based P values in our testing with the three hypotheses, in which deletion (labeled as Loss), amplification (labeled as Gain) and both (labeled as Abnm) were evaluated as abnormal separately, are plotted against the position on each chromosome. For clarity, P values that passed the SNP site-based testing are highlighted in green and the SNP sites that passed the window-based testing are highlighted in yellow.
(1.10 MB DOC)

Figure S2 Comparison with the previous genotype-association analysis demonstrates the priority of the CNV-association test in copy number variable regions. “Gen” and “Add” indicate the genotypic test and trend test, respectively, in the WTCCC paper. The −log10 of the SNP site-based P values in our test with the triple NULL hypothesis (Loss, Abnm and Gain) were plotted against the −log10 of the P values from the genotype association test from the WTCCC (A–G). For clarity, the genotype association P values<10−5 are highlighted in green, the CNV-association P values that passed the single SNP site-based testing are in blue and the CNV-association P values that passed the window-based testing are in red. SNPs absent from the genotype association analysis are plotted by default as zero and highlighted in brown, in which many SNPs that passed the SNP site-based testing are labeled with black.
(0.78 MB DOC)

Figure S3 Evidence that CNVs can lead to chaotic genotyping clusters in copy number variable regions. The selected sample-wide intensity maps show the typical influence of CNVs in the seven diseases labeled with abbreviations (A–F). All of the 17000 individuals are labeled with grey, individuals with CNVs in the disease group are in red and individuals with CNVs in controls are in green.
(0.49 MB DOC)

Figure S4 Selected CNV-loci that show strong evidence of association with diseases. The −log10 of the SNP site-based P values are plotted against the genomic location, in which the SNPs that passed the window-based testing are indicated in dark blue for the deletion hypothesis, dark green for the amplification hypothesis and orange for the deletion and amplification hypothesis. SNPs that lacked significance are shown in light colors (light blue for deletion, light green for amplification and yellow for both). Functionally affected regions were characterized within a 0.2 Mb region centered on the identified SNP sites, and the region boundary (vertical dashed line) coincided with the length limitations or the location of neighboring genes. The clustering heat map for 41 SNP windows (each corresponding to the upper CNV-region) demonstrated good CNV boundaries in and around the “first class node.” In the heat map, black indicates a copy number of 0, red a copy number of 1, light grey a copy number of 2 and green a copy number of 3.
(6.21 MB DOC)

Figure S5 The cartoon depicts the function of the calcium-related pathway in bipolar disorder. The Ca2+/IP3 pathway has been reported to be closely related to bipolar disorder, and the molecules revealed in previous studies are labeled with blue circles and in red font. IP3 precursors in the membrane and metabolites in the cytosol are denoted by different shaped boxes. INPP5B, POU3F, Olfactory receptors (belonging to GPCR, G Protein-Coupled Receptors) and KCNQ5, which were found to be associated to CNVs in our work, are labeled with black font and orange circles (or boxes).
(0.09 MB DOC)

Table S1 List of SNP sites showing significance in the window-based testing.
(0.63 MB DOC)

Table S2 22 risk genes that were validated from previous studies.
(0.20 MB DOC)

Text S1 Supporting methods of multiple testing for trend.
(0.08 MB DOC)
