# Supplementary figures and images for: Novel Association Strategy with Copy Number Variation for Identifying New Risk Loci of Human Diseases
Source: PLoS One. 2010 Aug 20;5(8):e12185. doi: 10.1371/journal.pone.0012185 (PMC2924882; doi:10.1371/journal.pone.0012185)

**A**

**
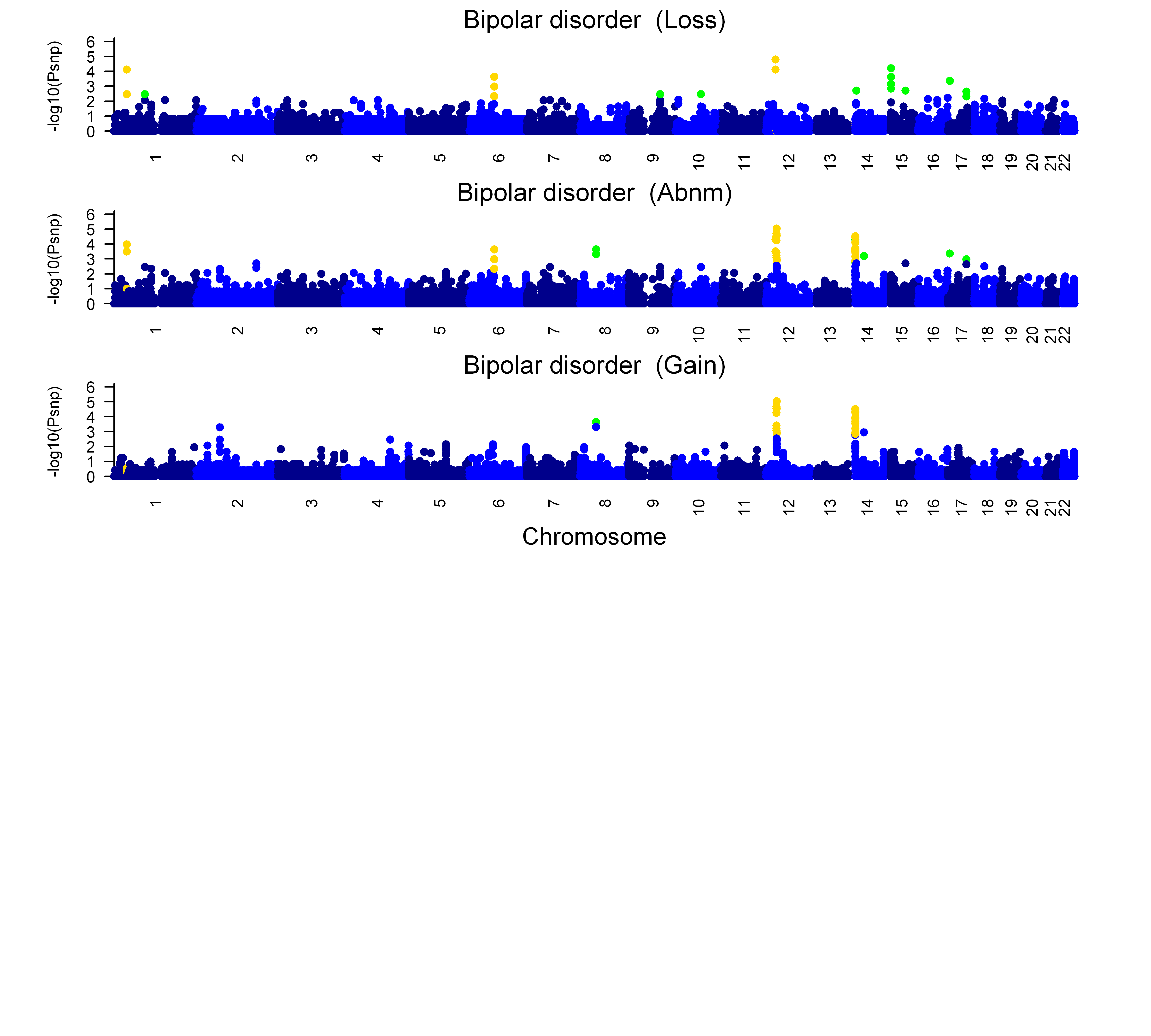
**

**B**

**
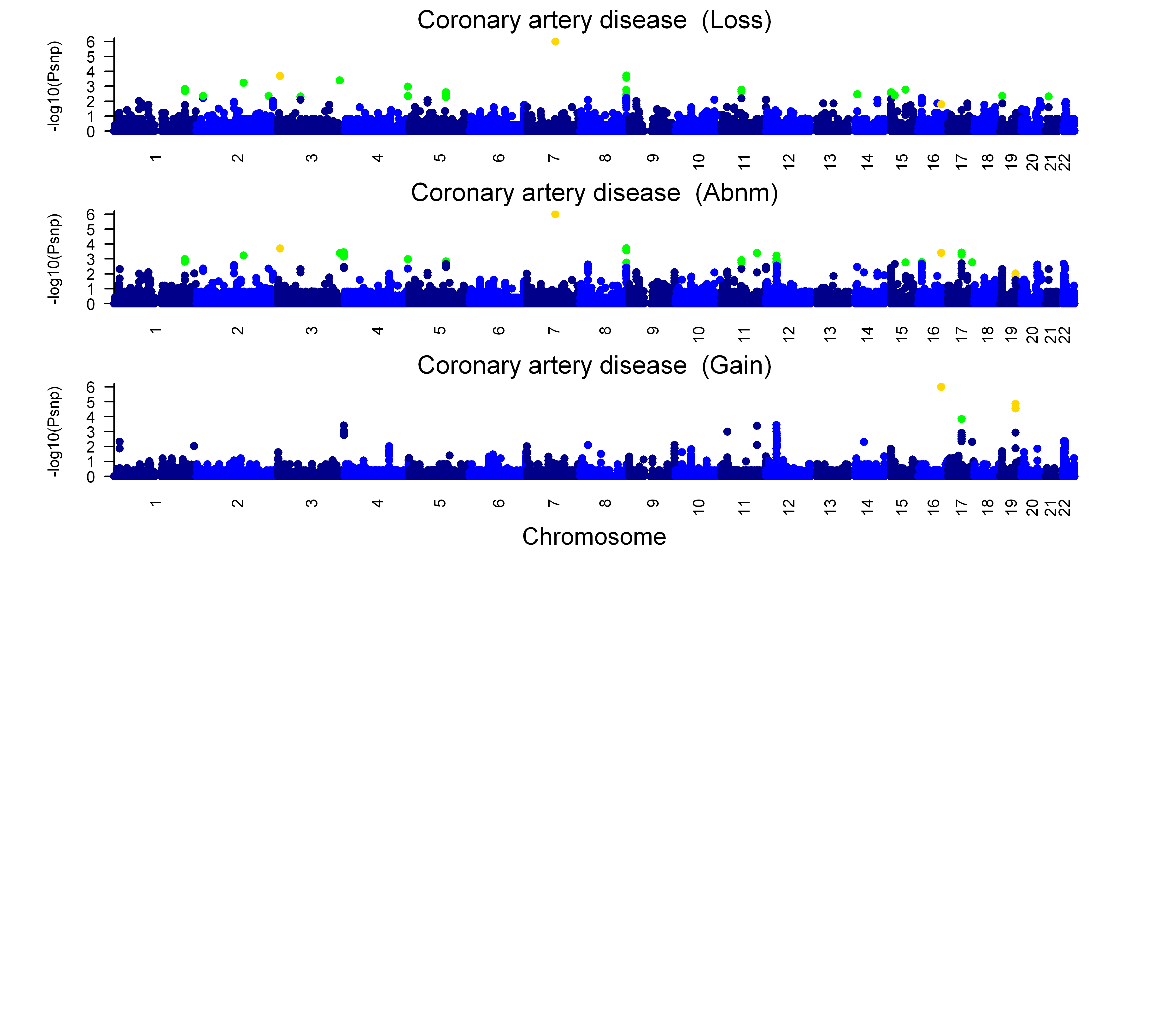
**

**C**

**
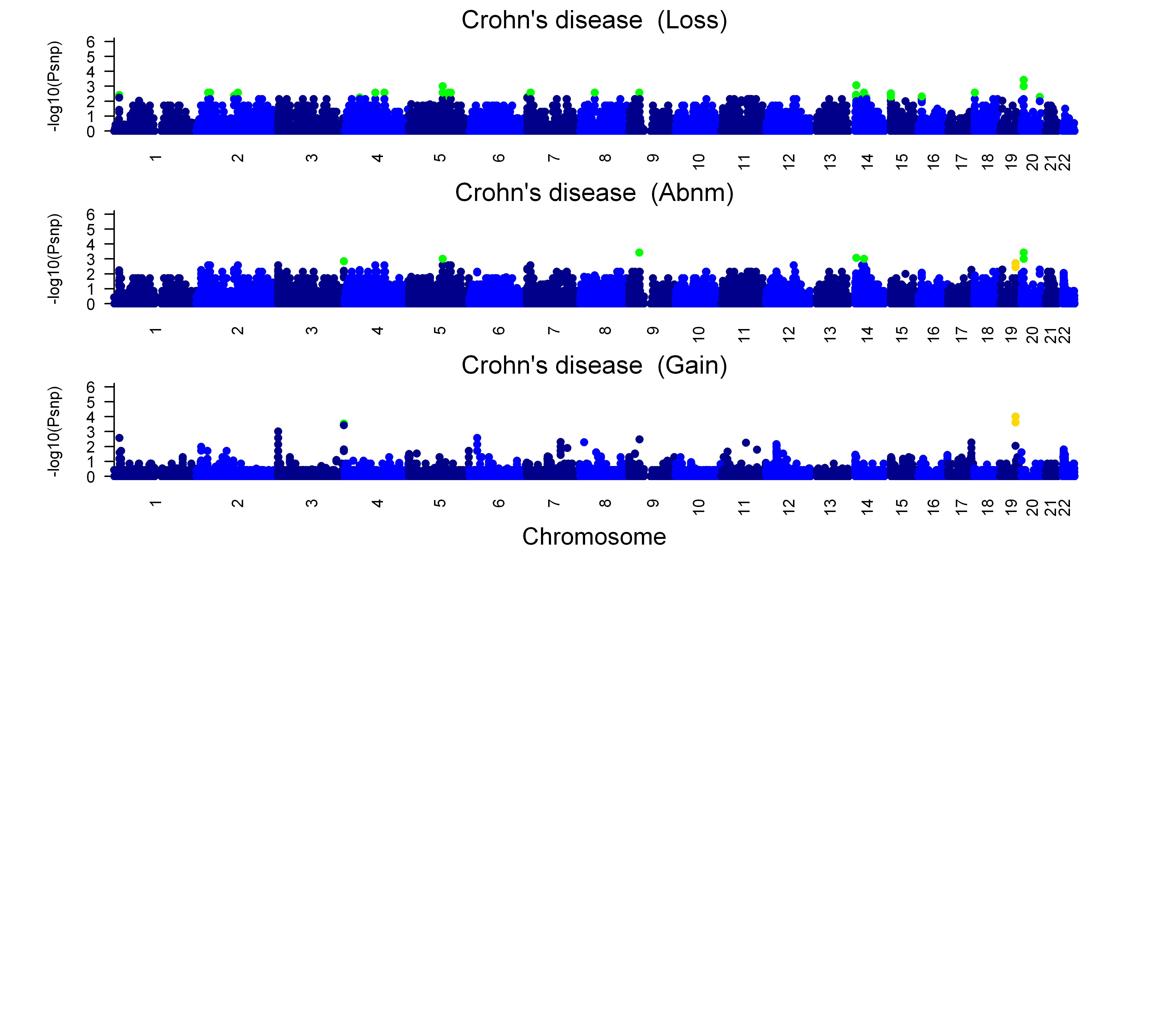
**

**D**

**
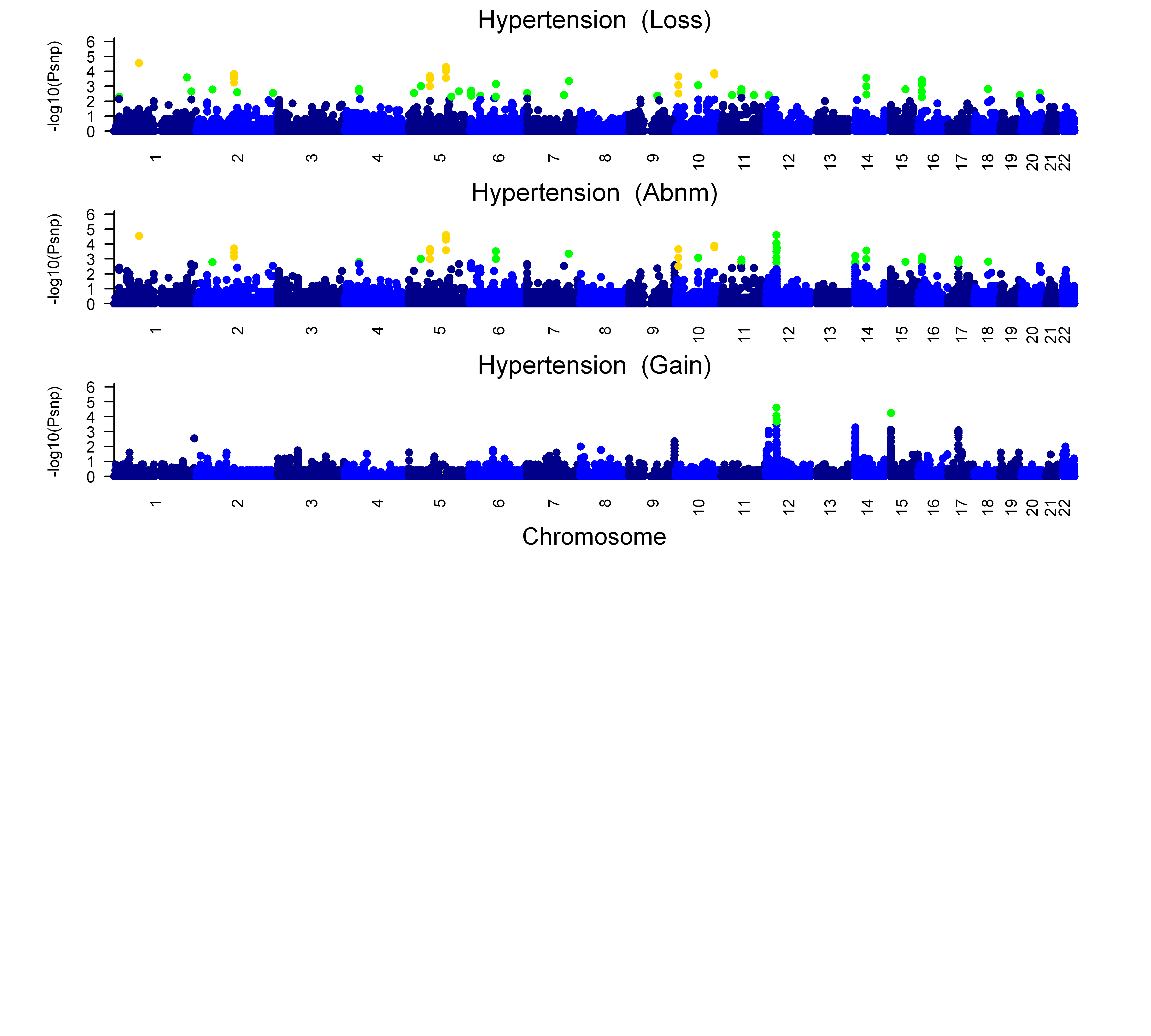
**

**E**

**
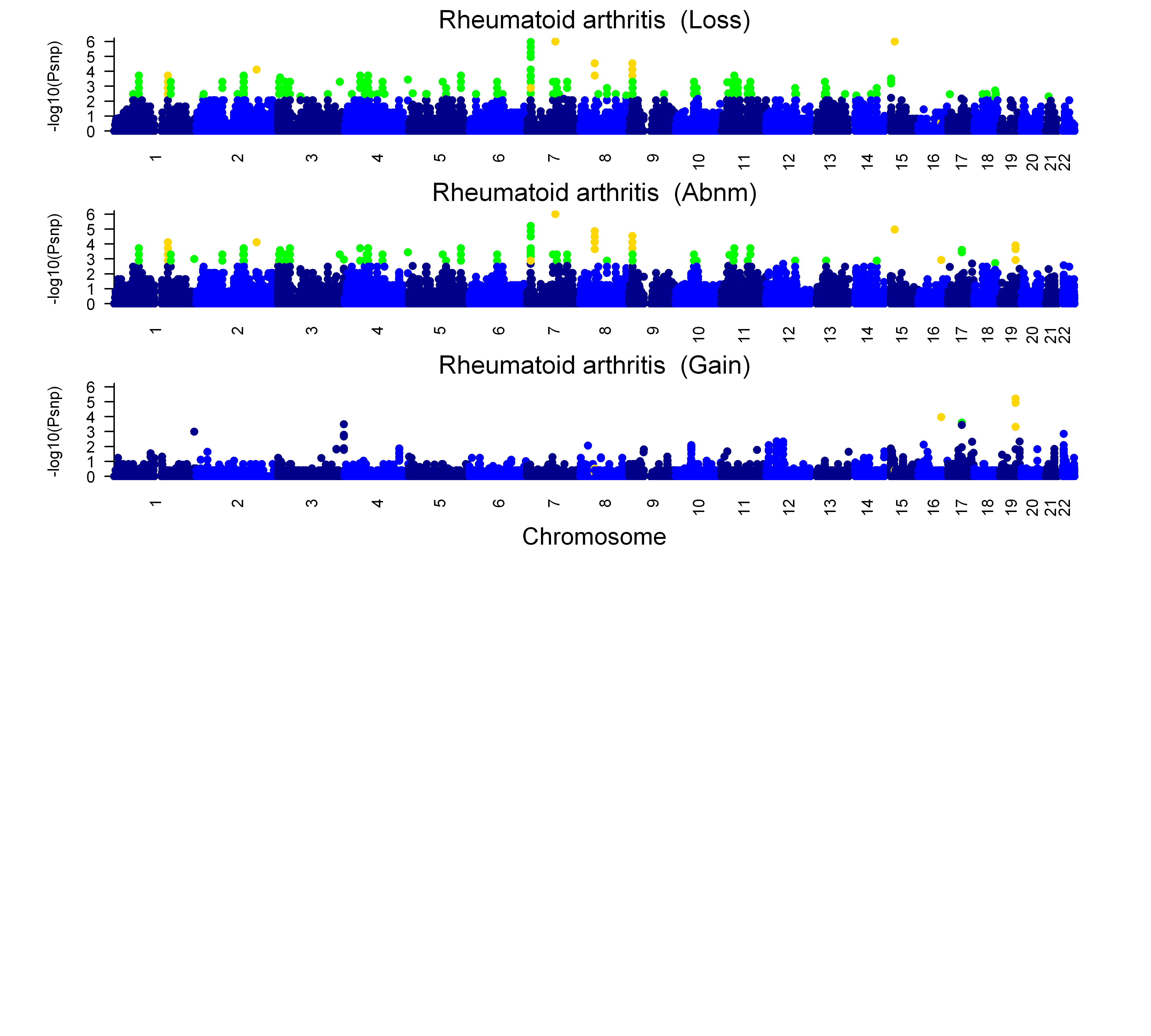
**

**F**

**
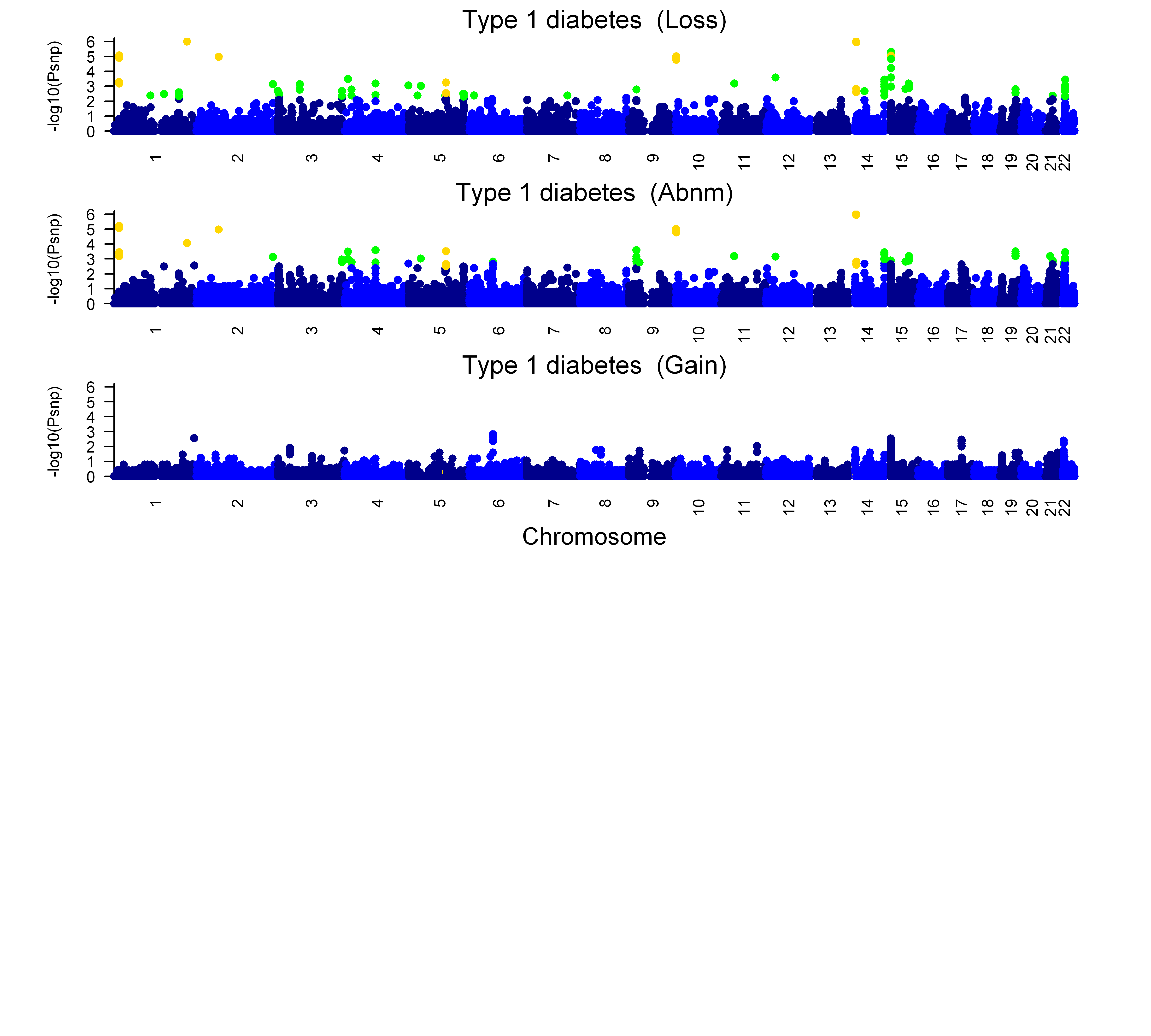
**

**G**

**
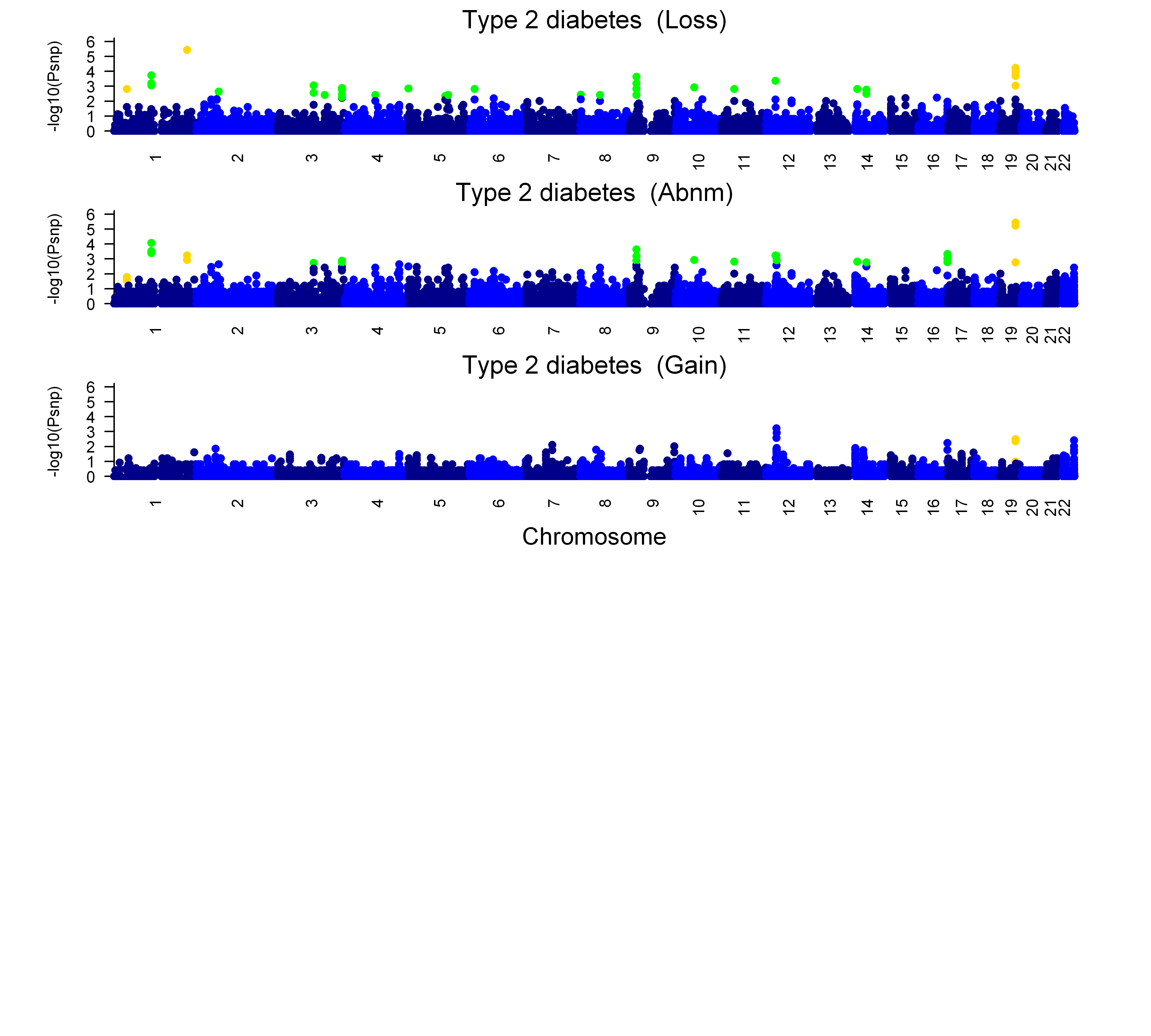
**

Supplement: Figure S1 — The genome-wide distribution of the CNV-association results in the seven diseases. The −log10 of the SNP site-based P values in our testing with the three hypotheses, in which deletion (labeled as Loss), amplification (labeled as Gain) and both (labeled as Abnm) were evaluated as abnormal separately, are plotted against the position on each chromosome. For clarity, P values that passed the SNP site-based testing are highlighted in green and the SNP sites that passed the window-based testing are highlighted in yellow. (1.10 MB DOC) [file pone.0012185.s001.doc]

**A**


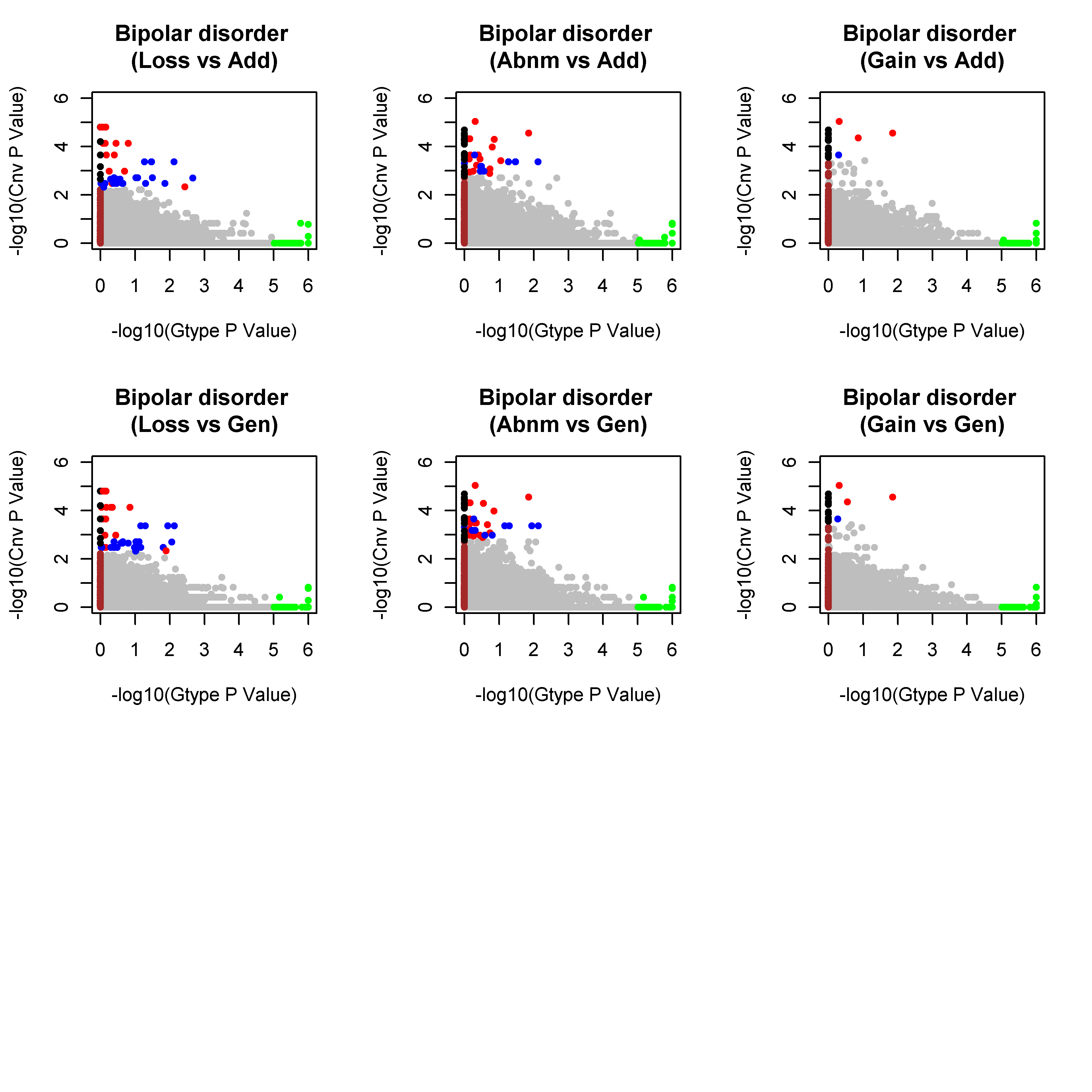


**B**


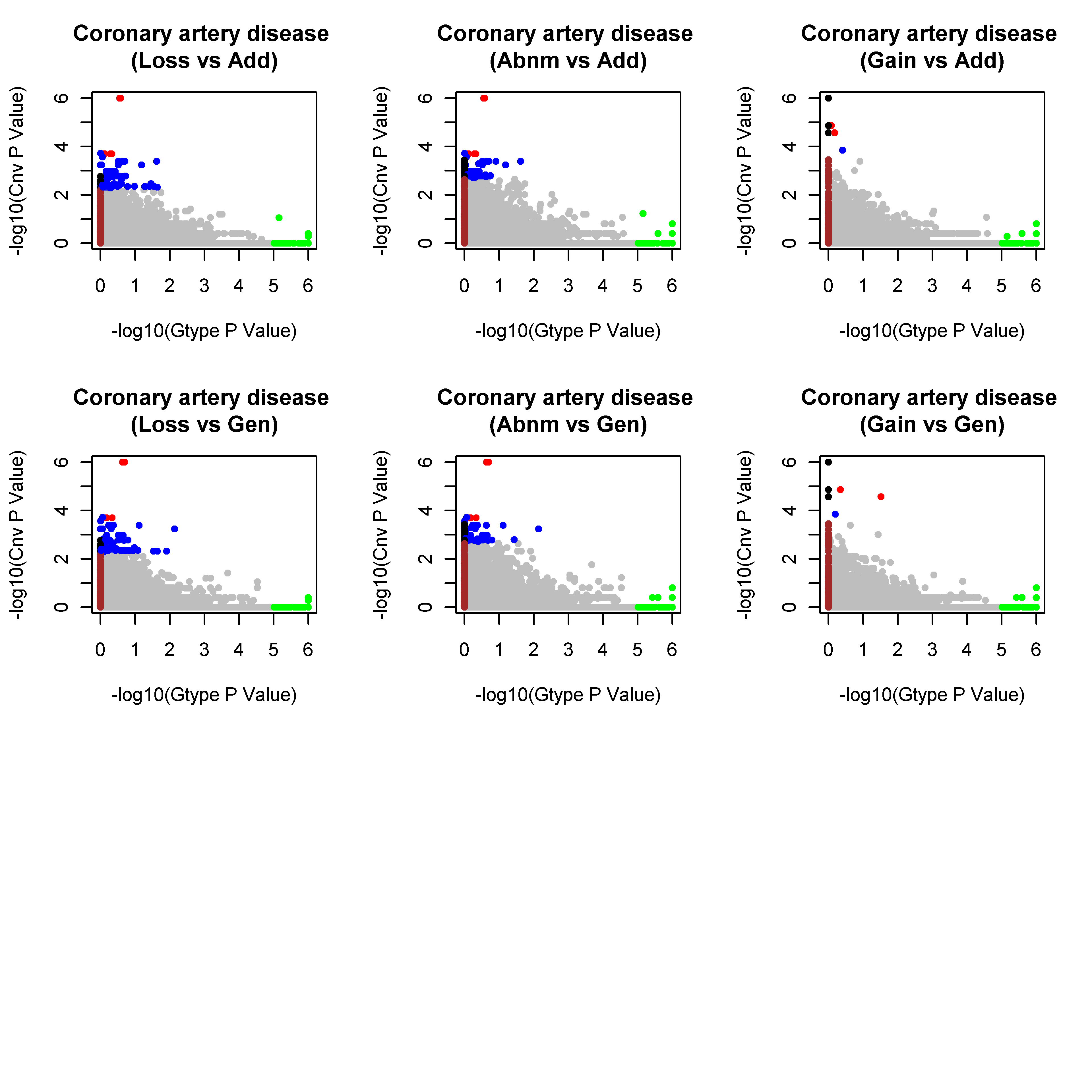


**C**


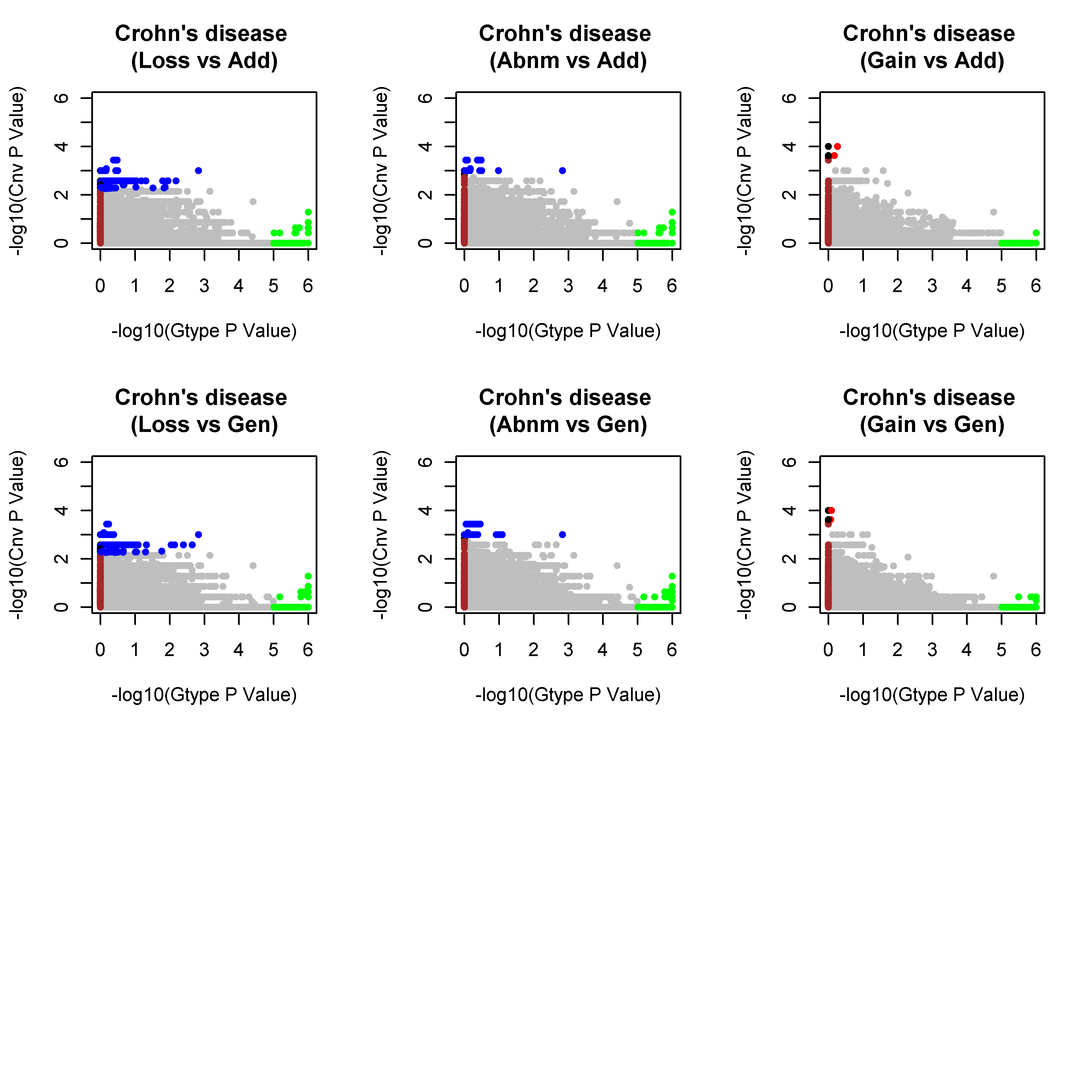


**D**


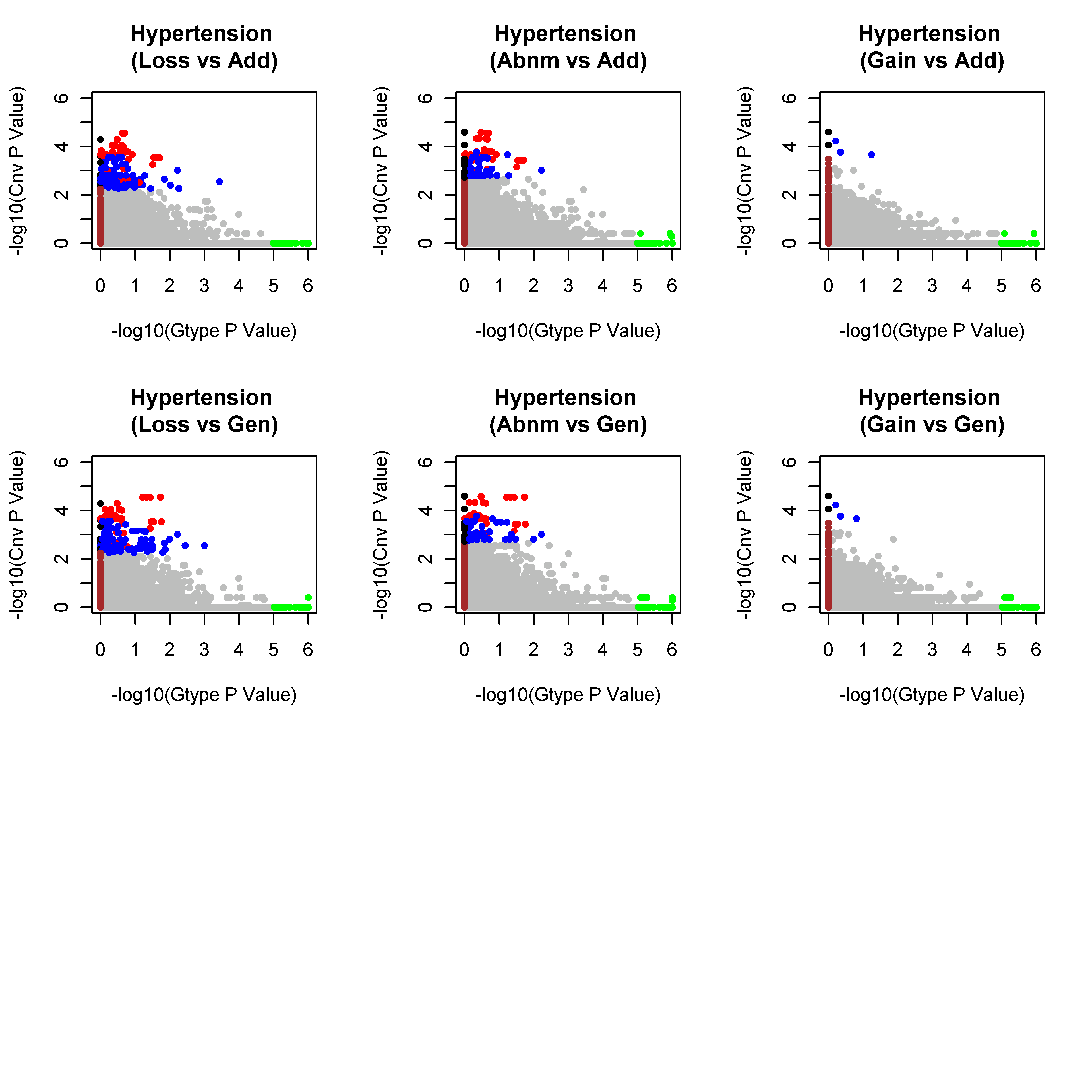


**E**


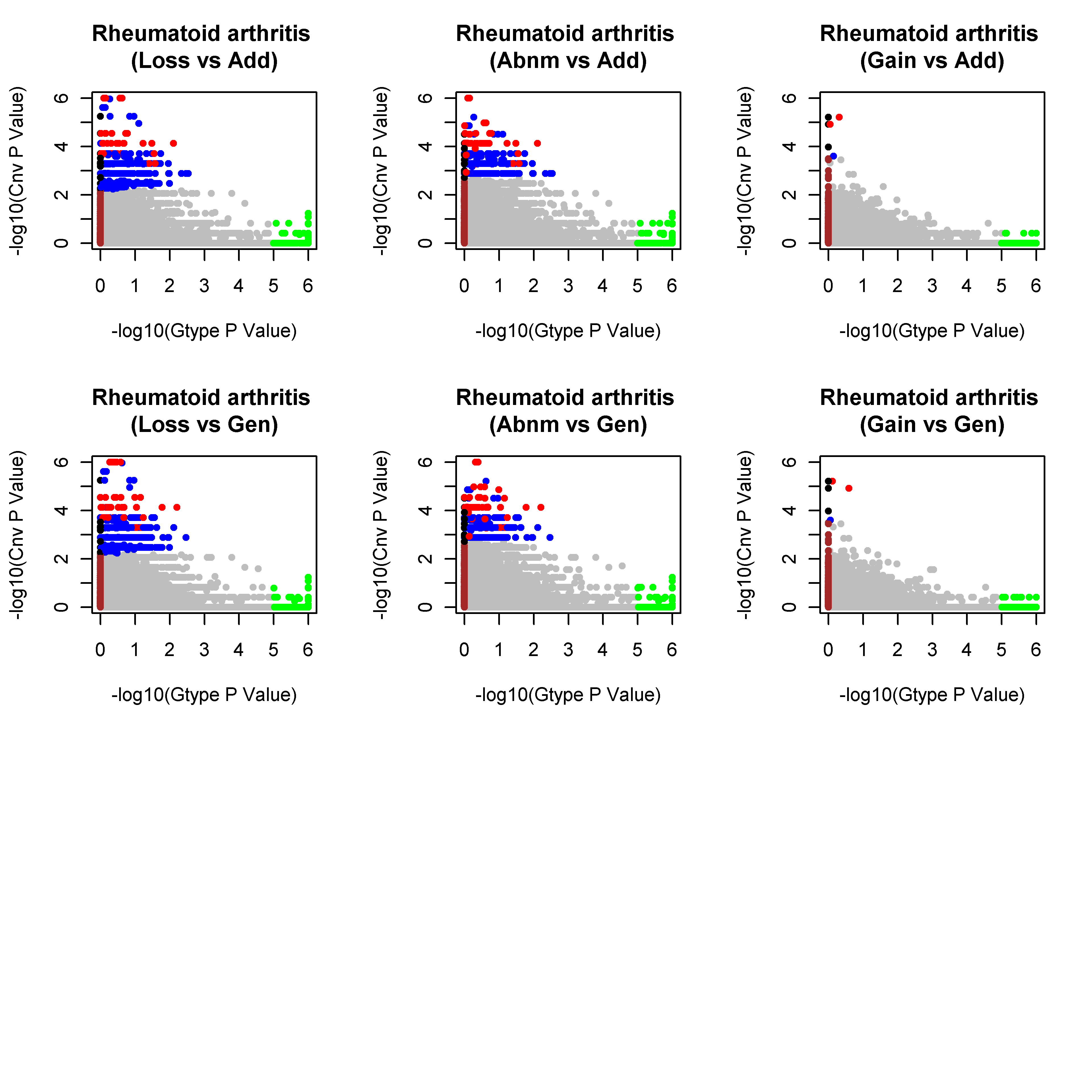


**F**


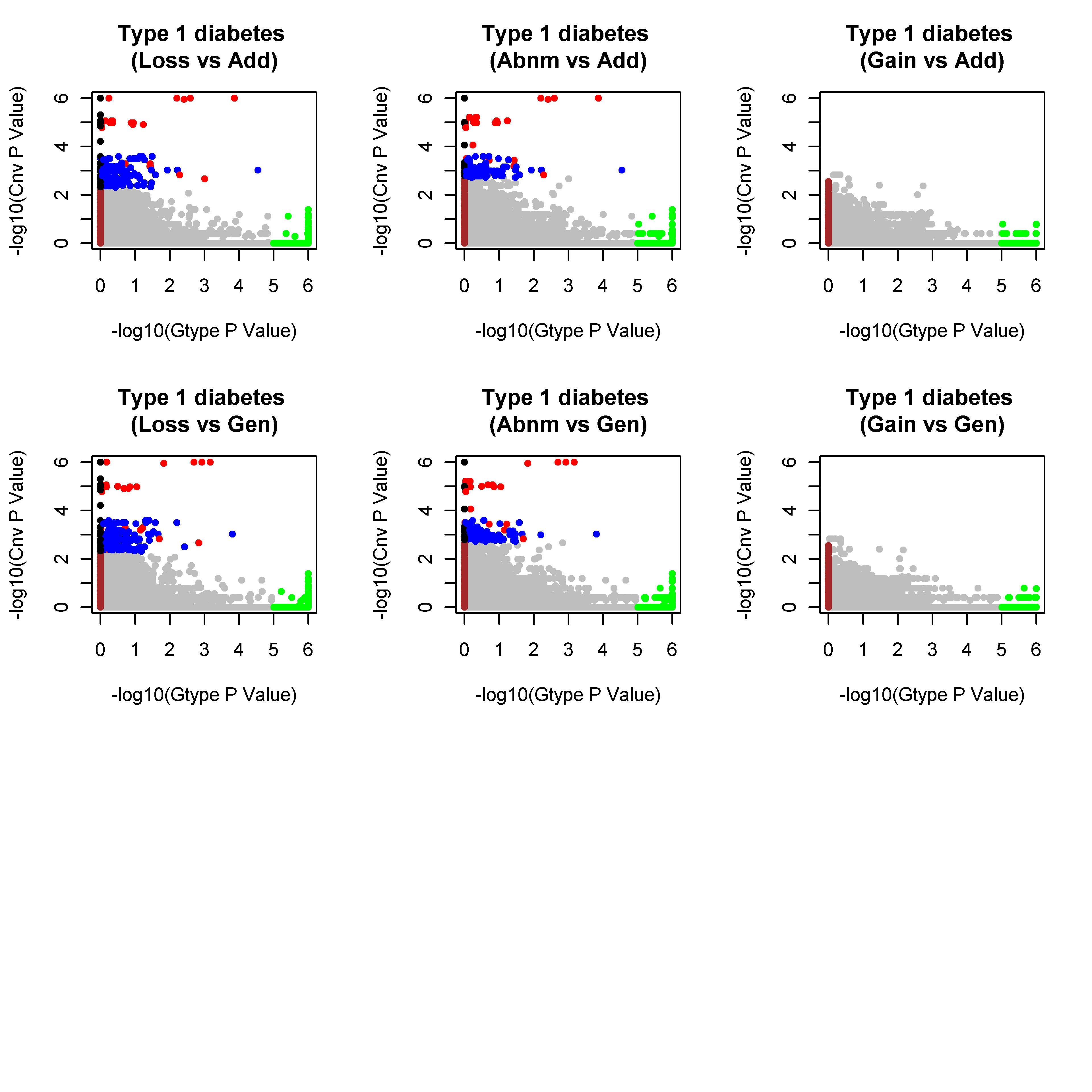


**G**


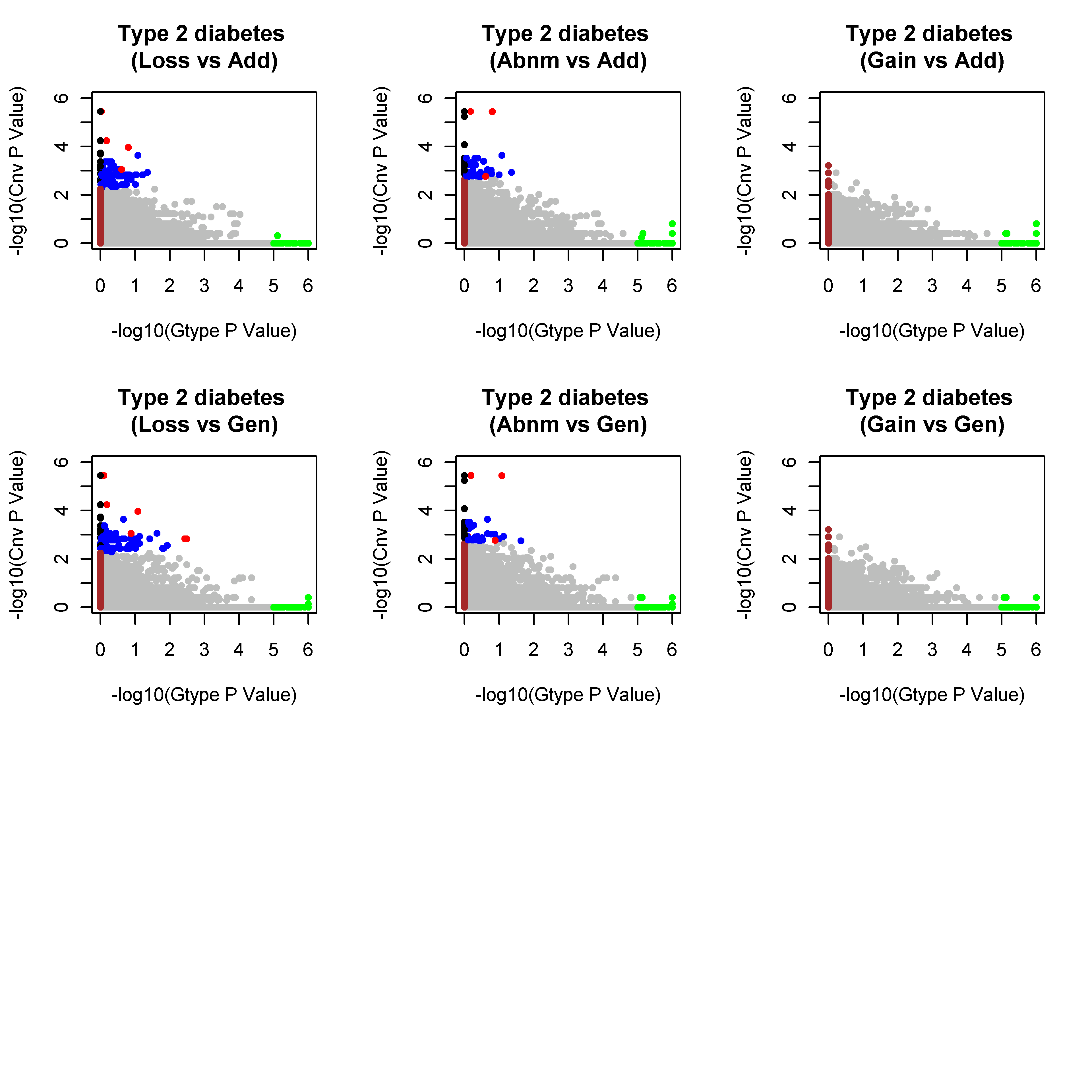

Supplement: Figure S2 — Comparison with the previous genotype-association analysis demonstrates the priority of the CNV-association test in copy number variable regions. “Gen” and “Add” indicate the genotypic test and trend test, respectively, in the WTCCC paper. The −log10 of the SNP site-based P values in our test with the triple NULL hypothesis (Loss, Abnm and Gain) were plotted against the −log10 of the P values from the genotype association test from the WTCCC (A–G). For clarity, the genotype association P values<10−5 are highlighted in green, the CNV-association P values that passed the single SNP site-based testing are in blue and the CNV-association P values that passed the window-based testing are in red. SNPs absent from the genotype association analysis are plotted by default as zero and highlighted in brown, in which many SNPs that passed the SNP site-based testing are labeled with black. (0.78 MB DOC) [file pone.0012185.s002.doc]

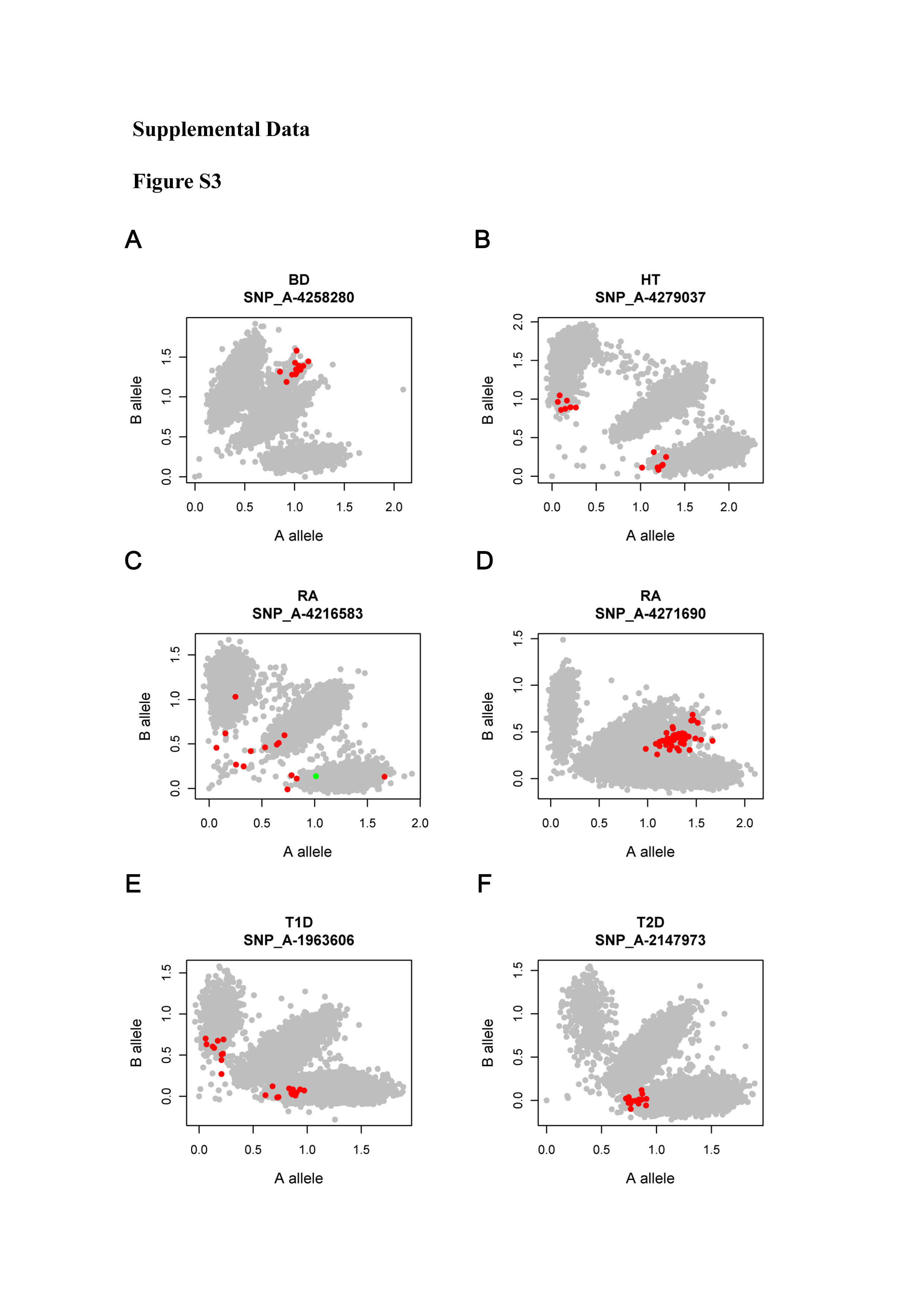

Supplement: Figure S3 — Evidence that CNVs can lead to chaotic genotyping clusters in copy number variable regions. The selected sample-wide intensity maps show the typical influence of CNVs in the seven diseases labeled with abbreviations (A–F). All of the 17000 individuals are labeled with grey, individuals with CNVs in the disease group are in red and individuals with CNVs in controls are in green. (0.49 MB DOC) [file pone.0012185.s003.doc]

**A**


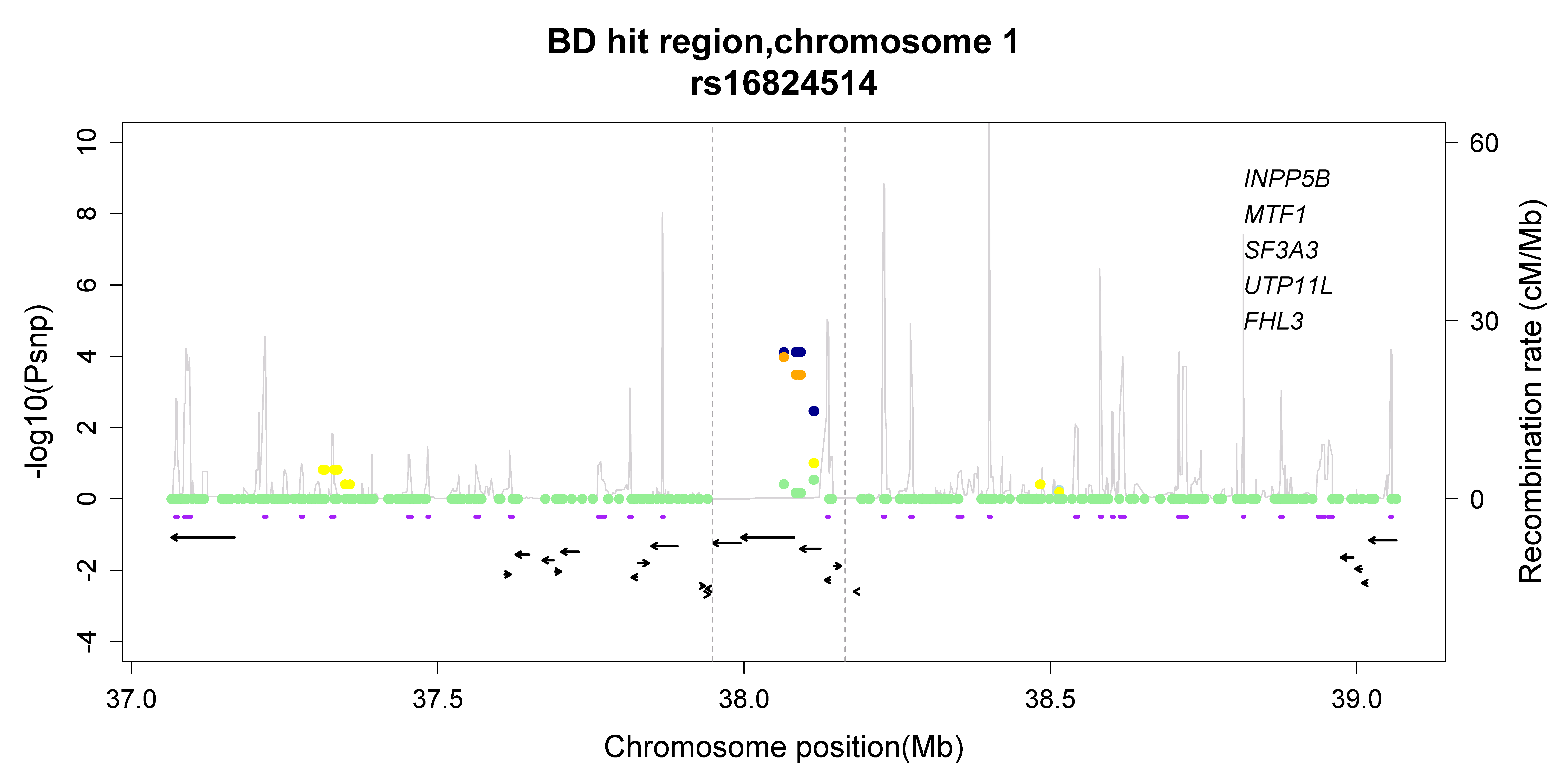


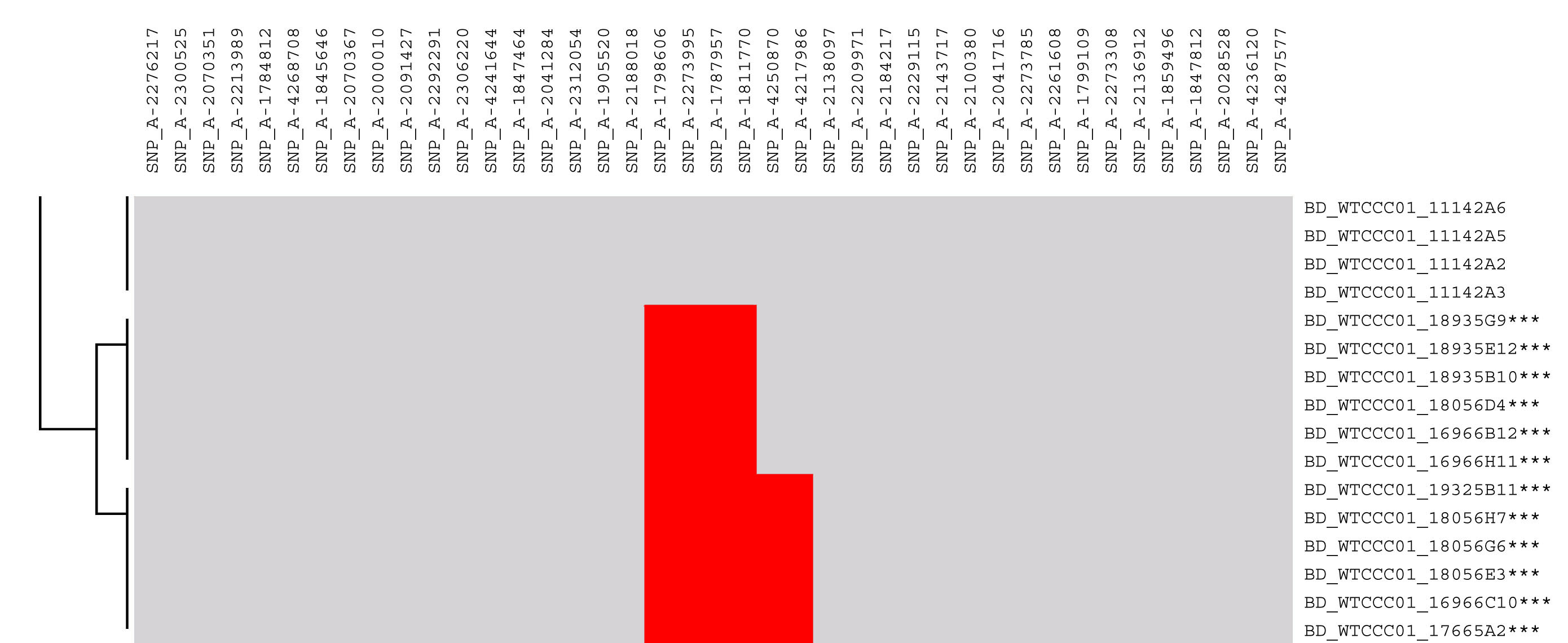


**B**

**
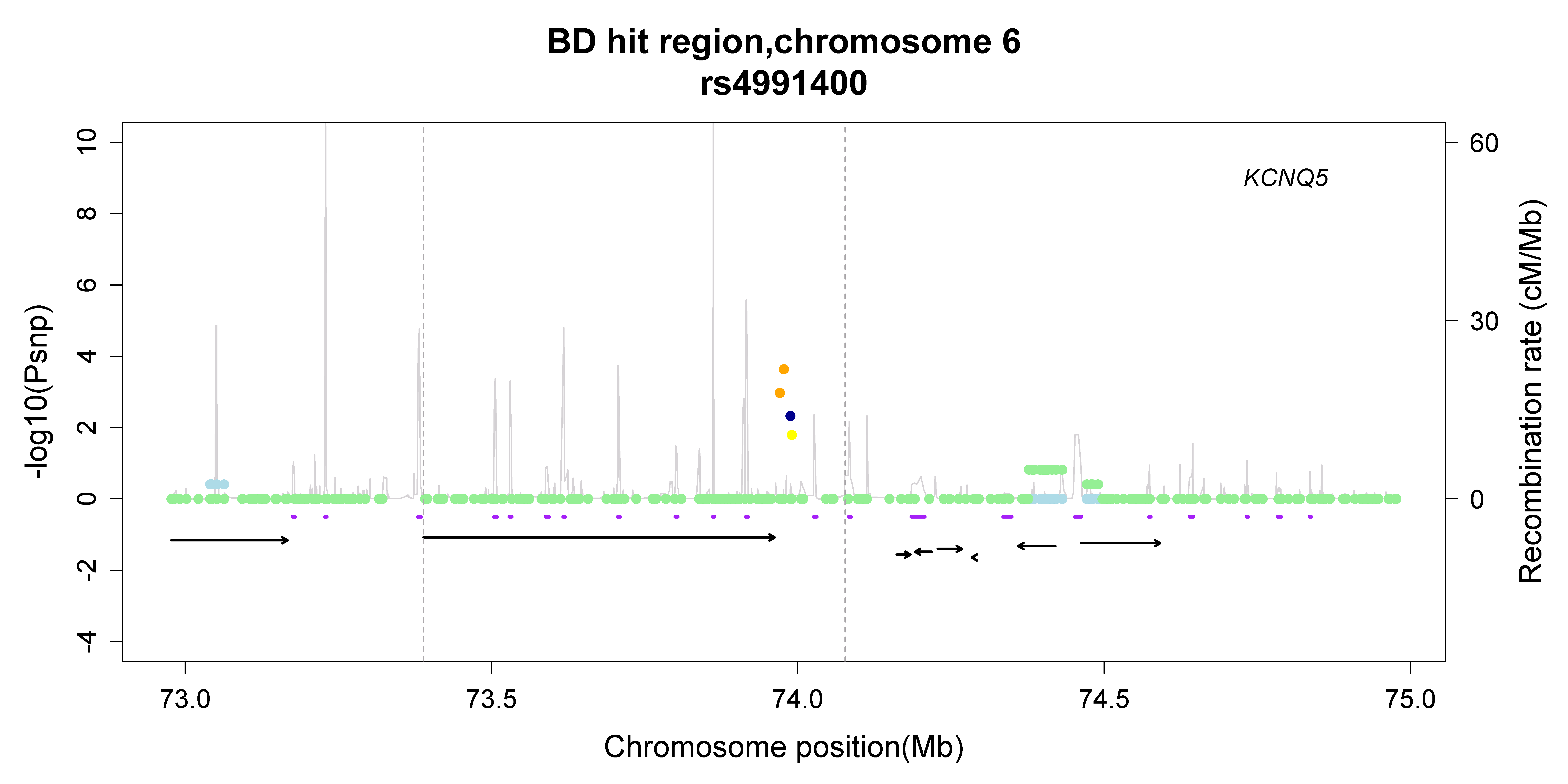
**

**
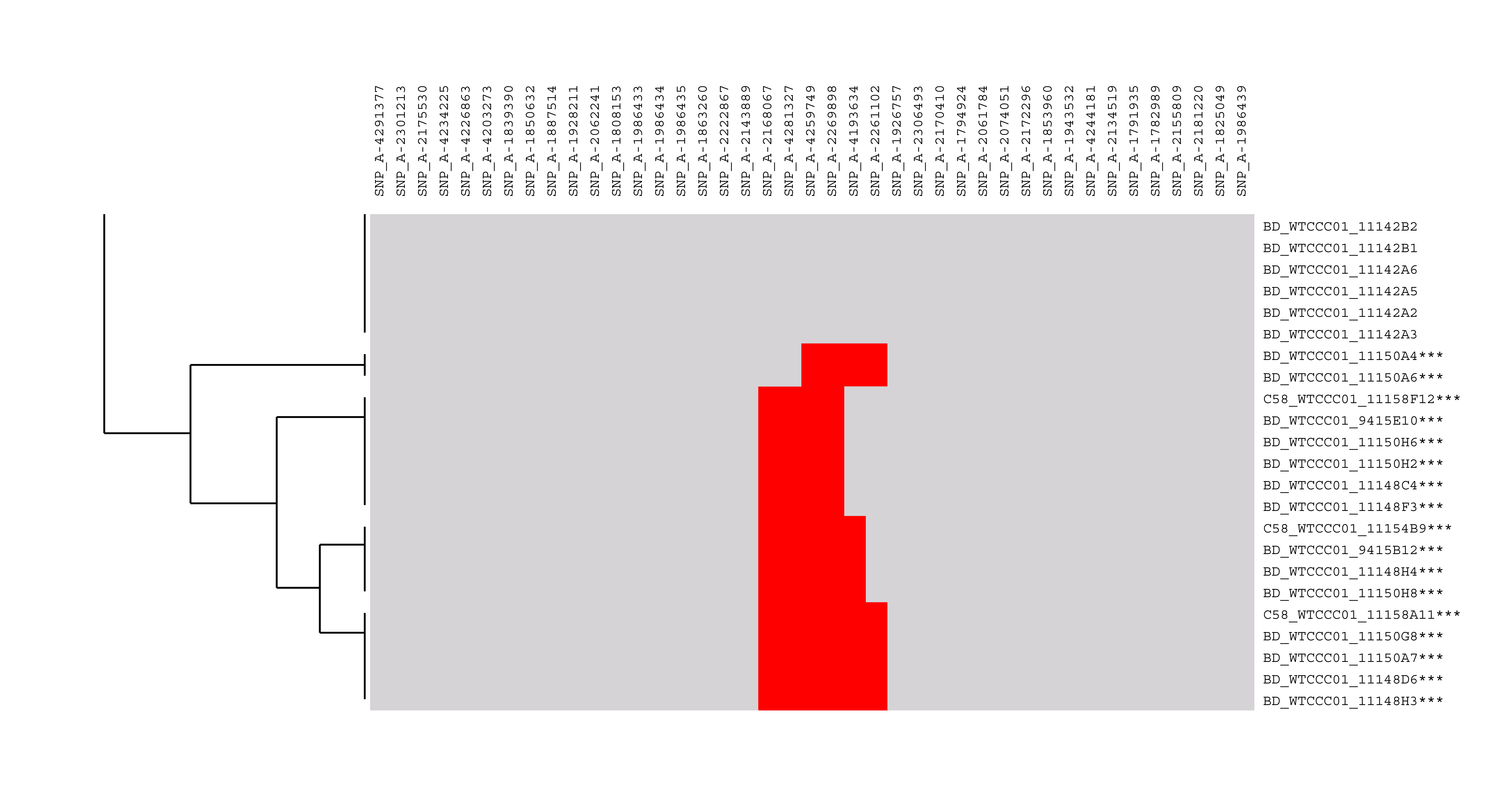
**

**C**


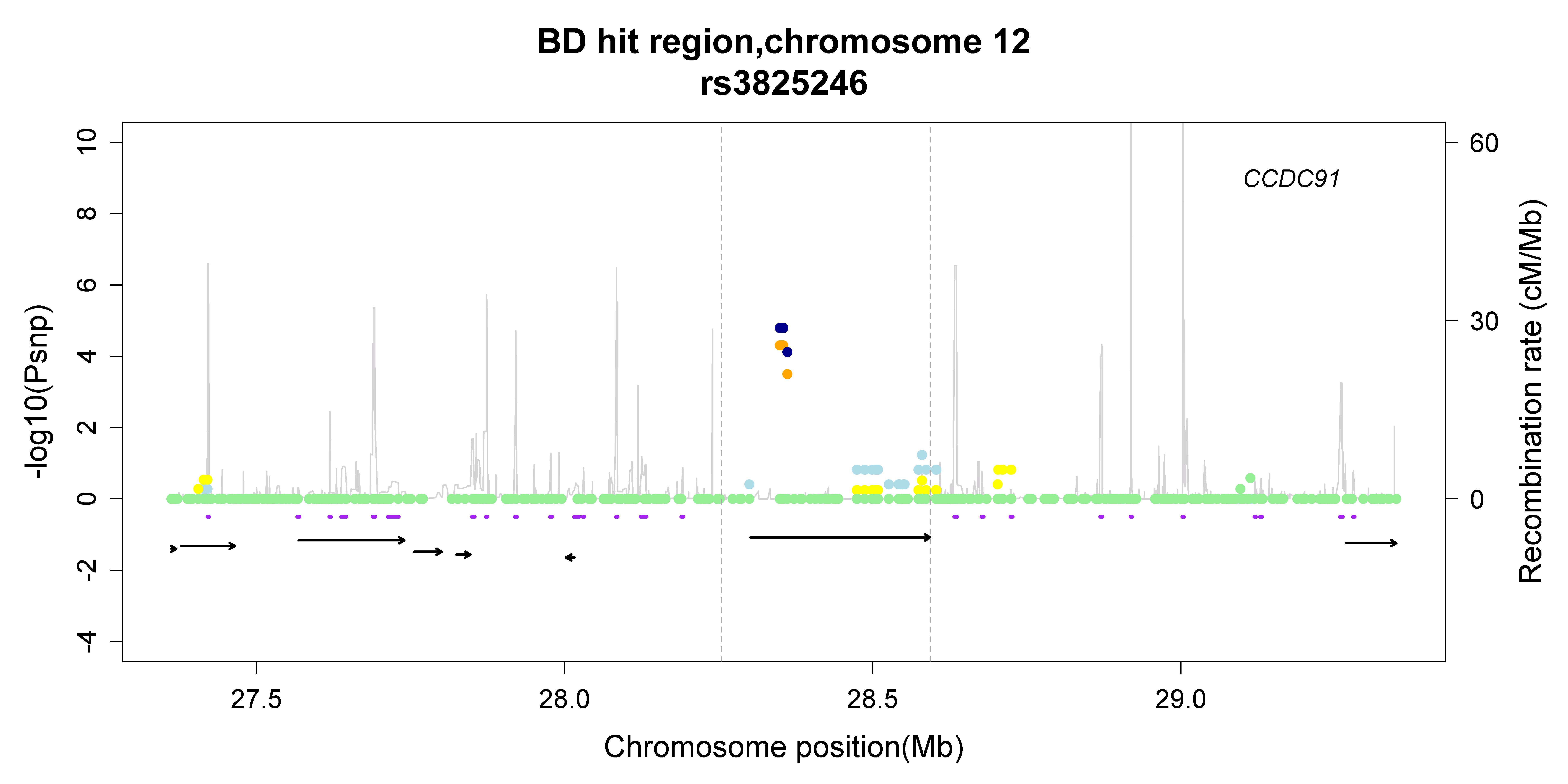


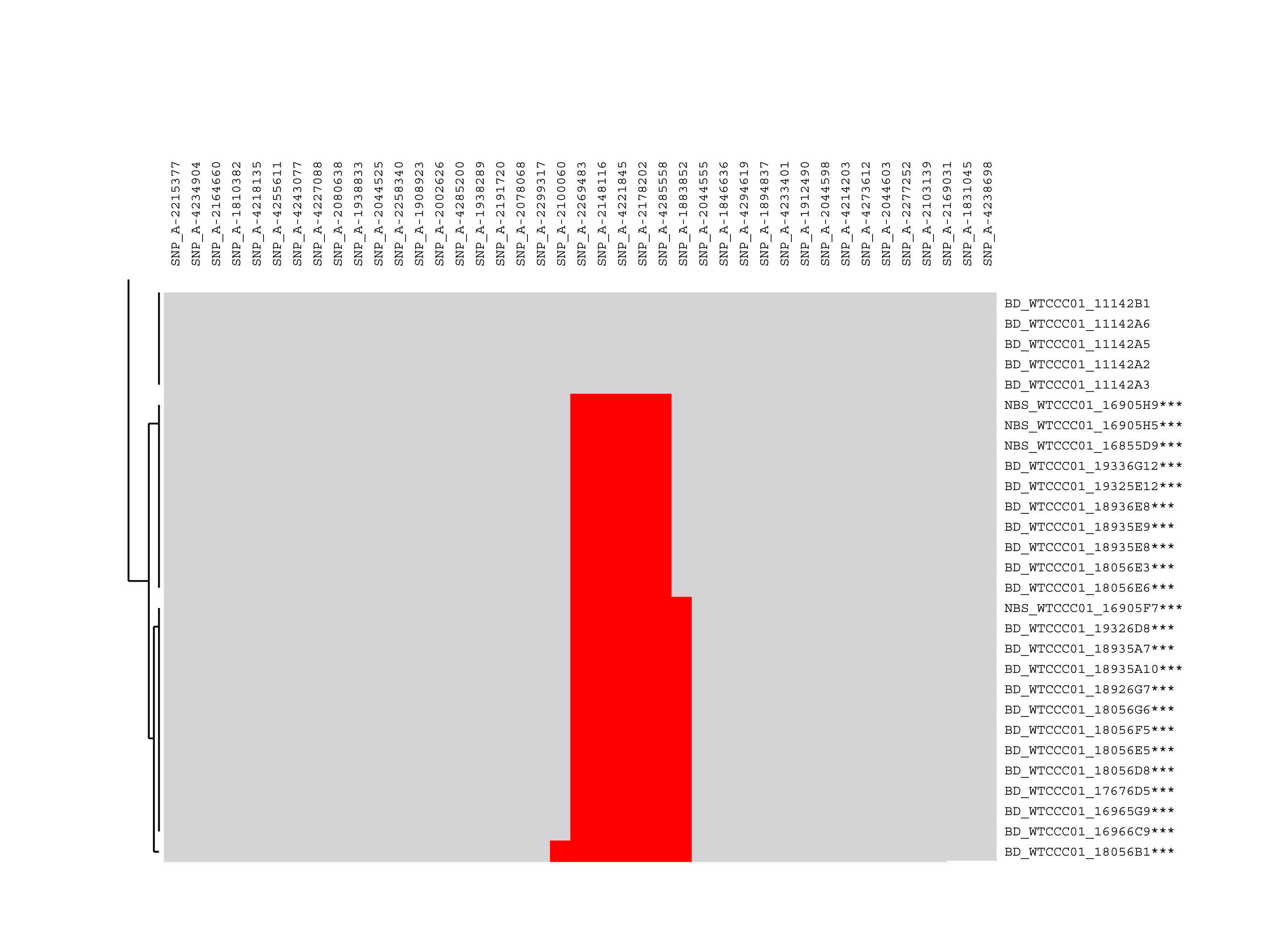


**D**


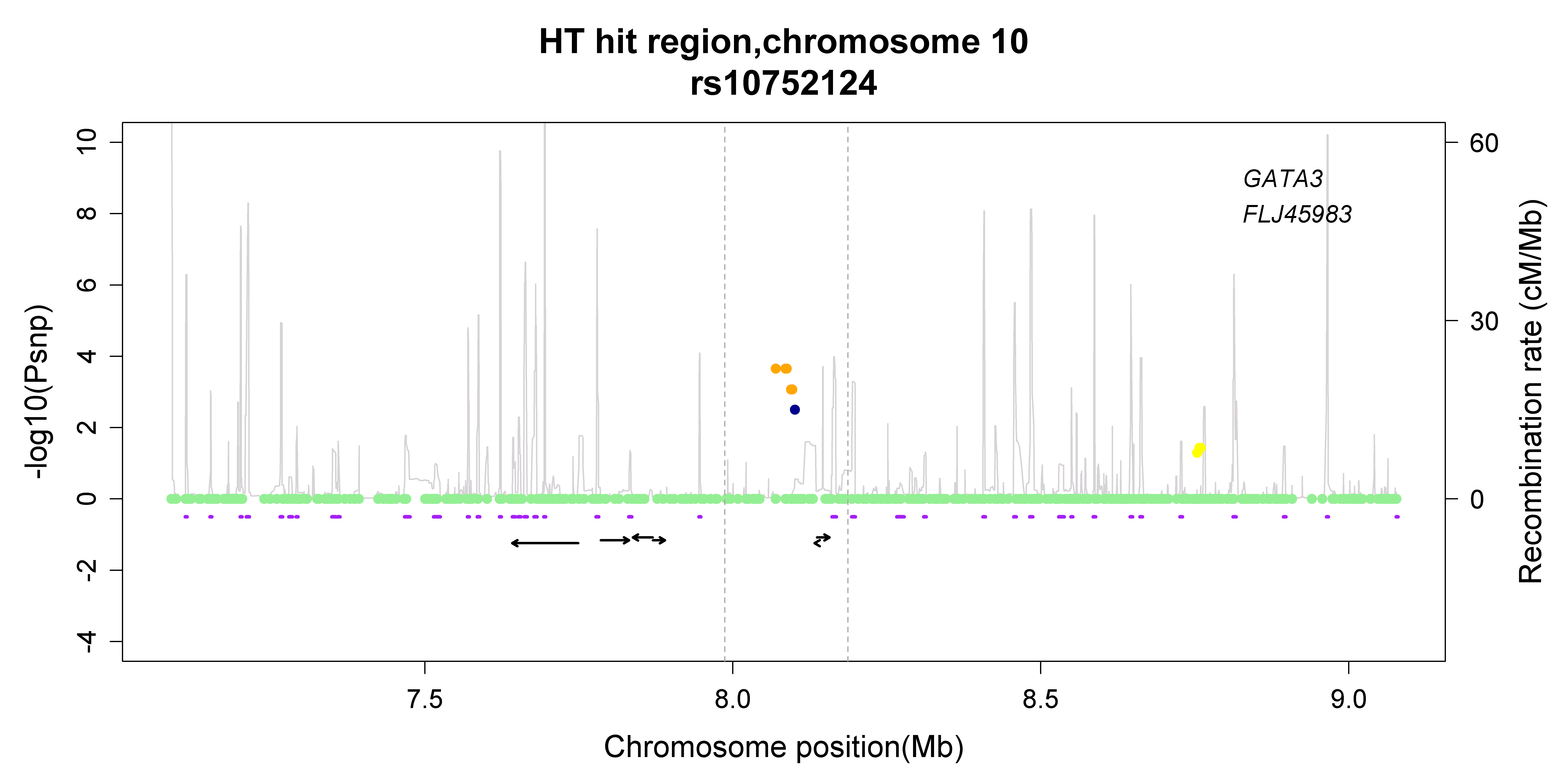


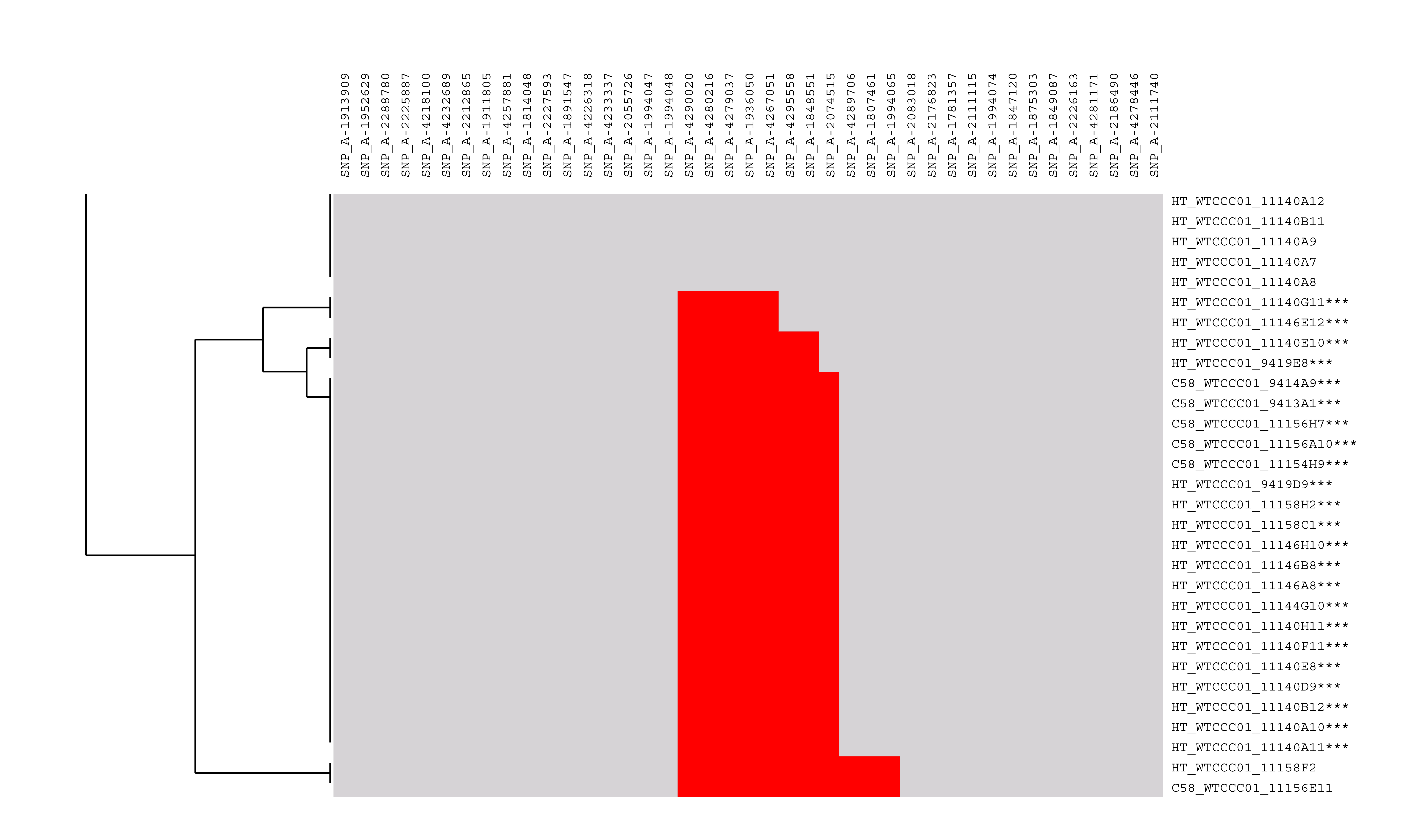


**E**

**
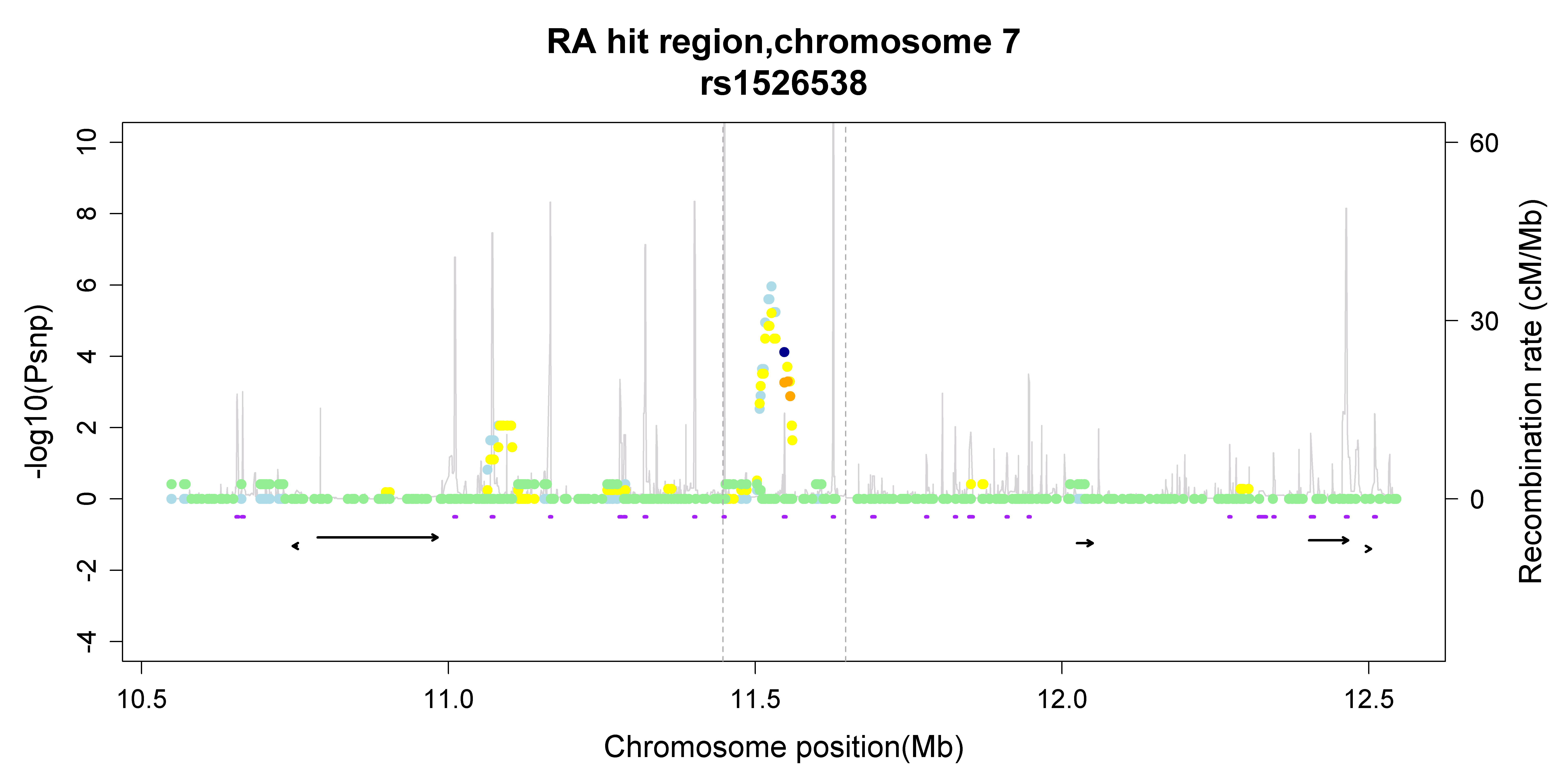
**


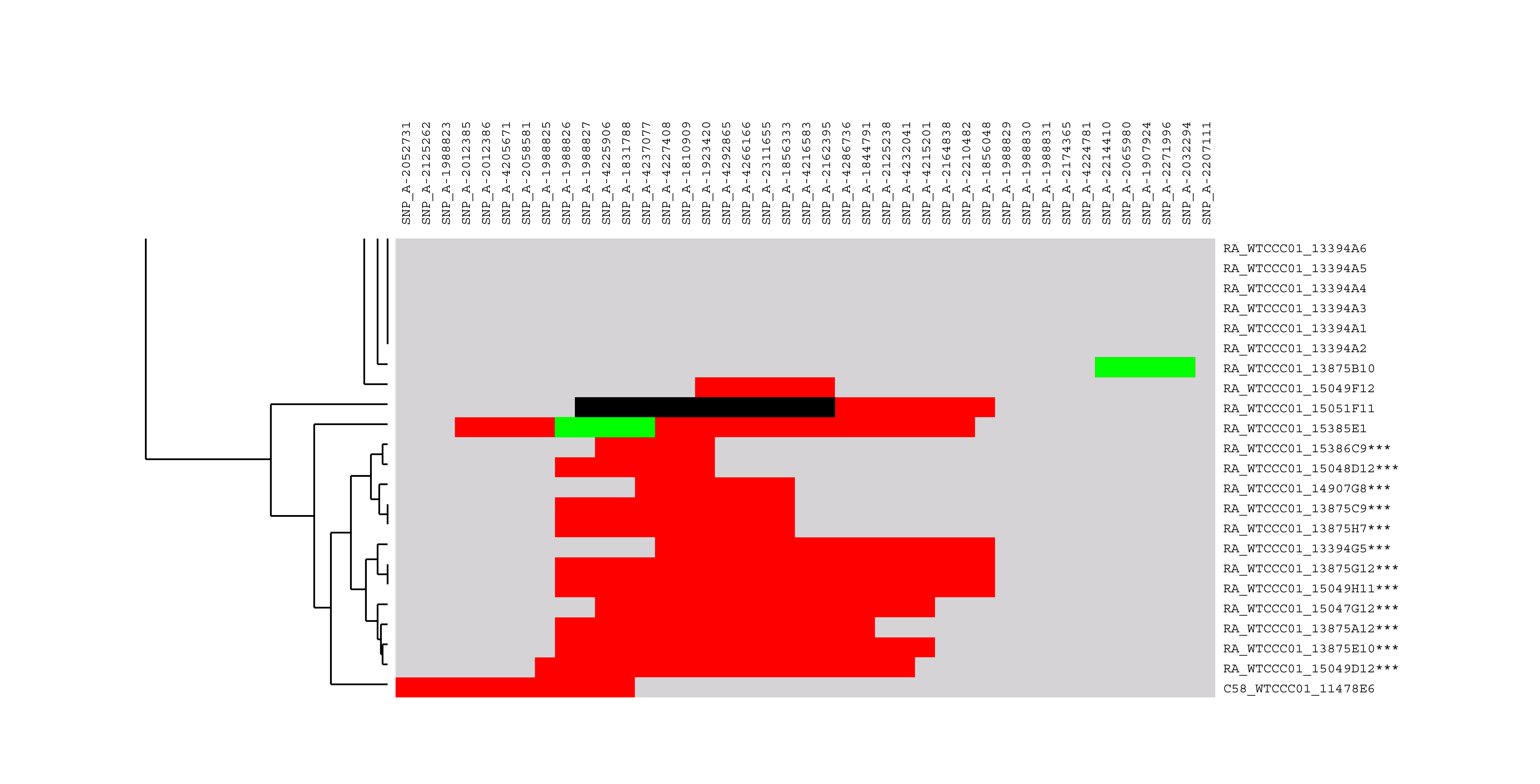


**F**


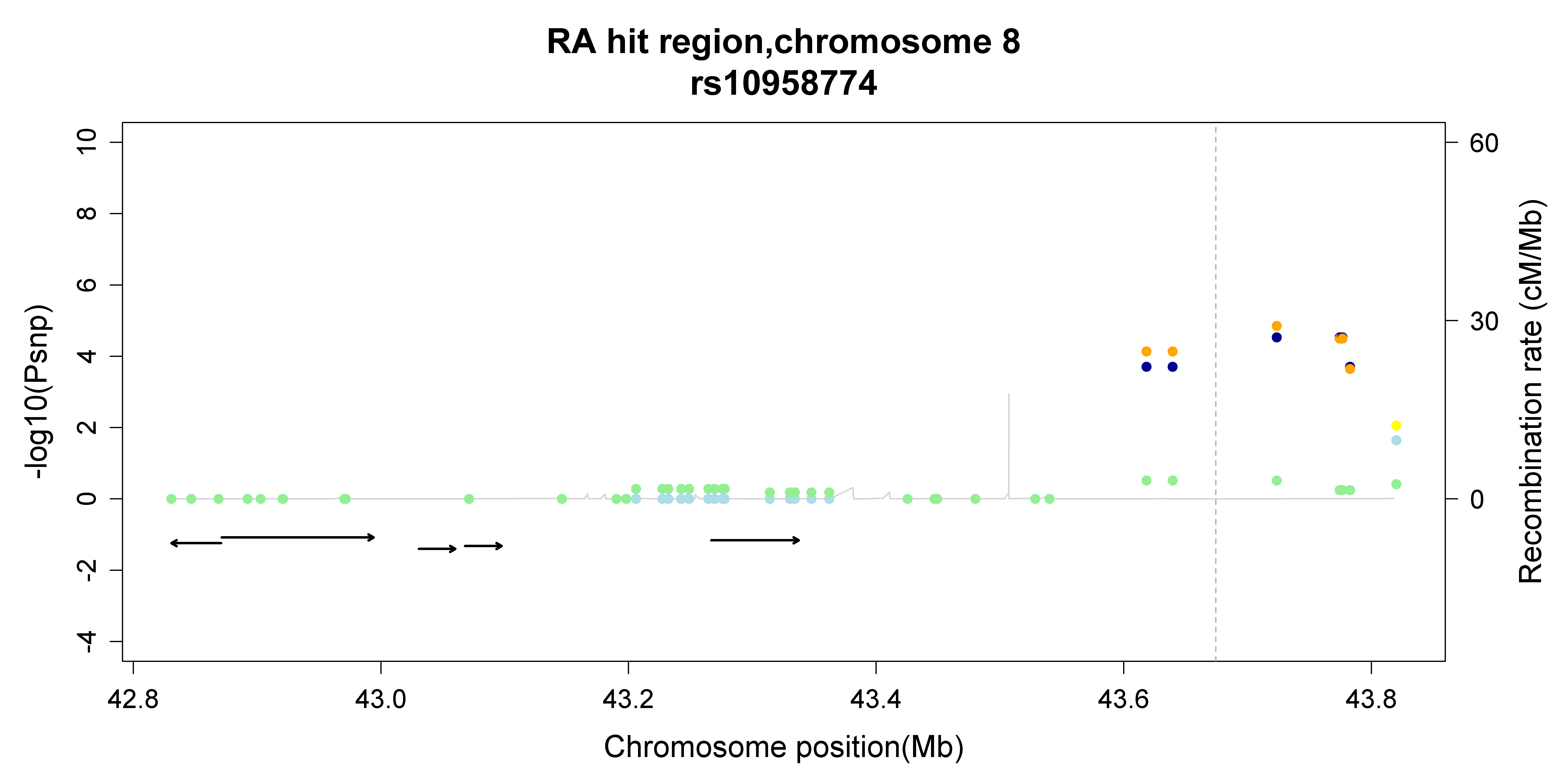


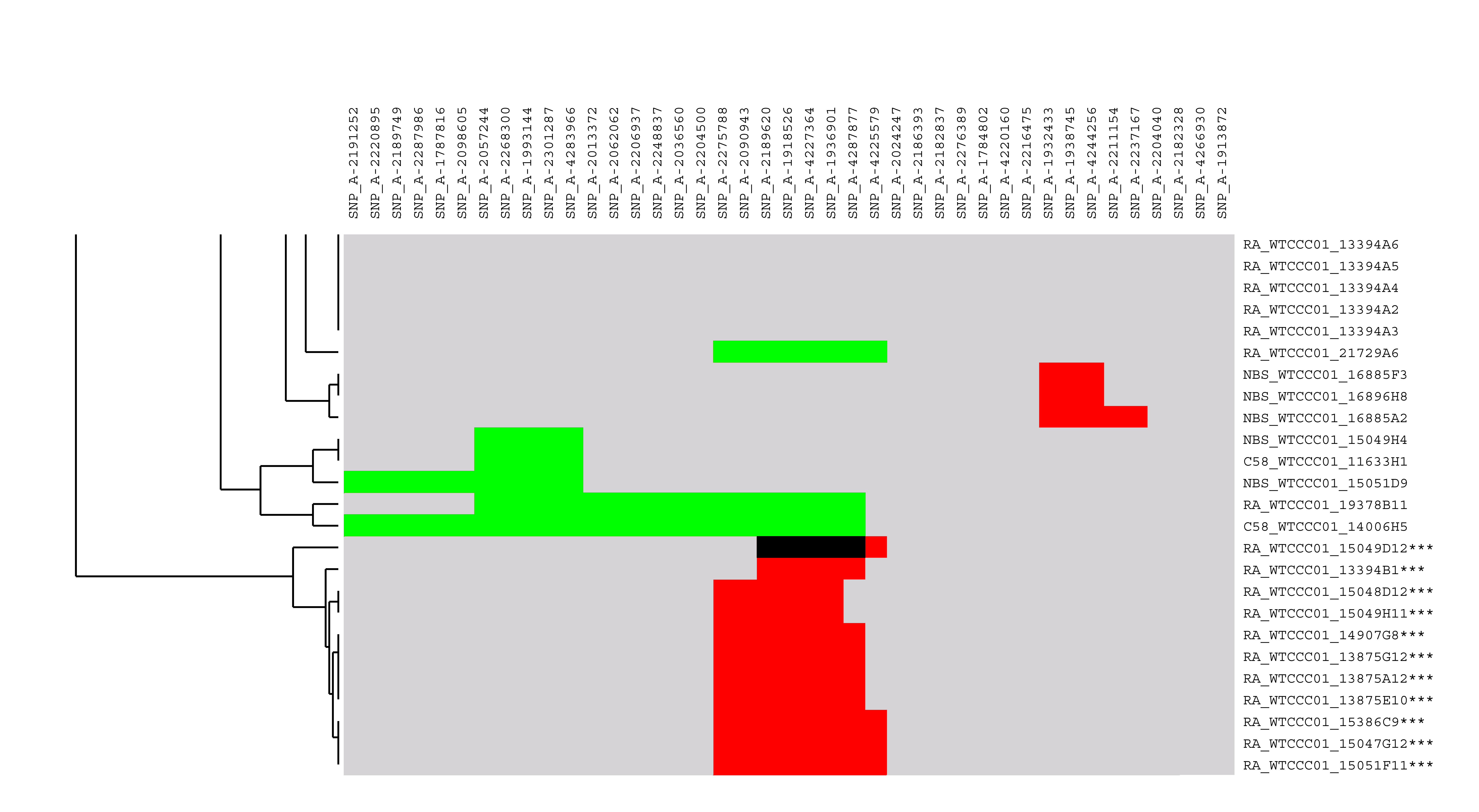


**G**


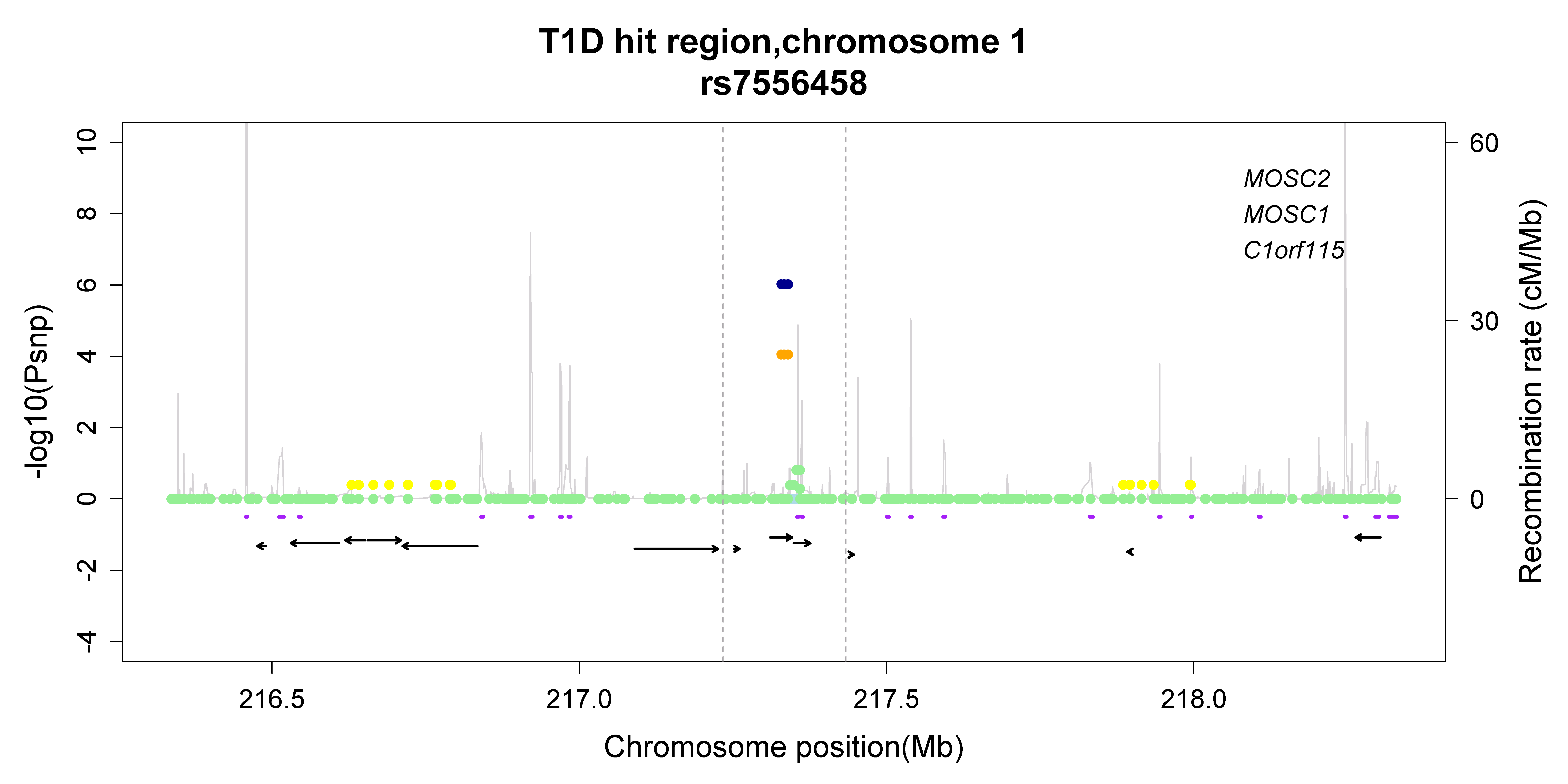


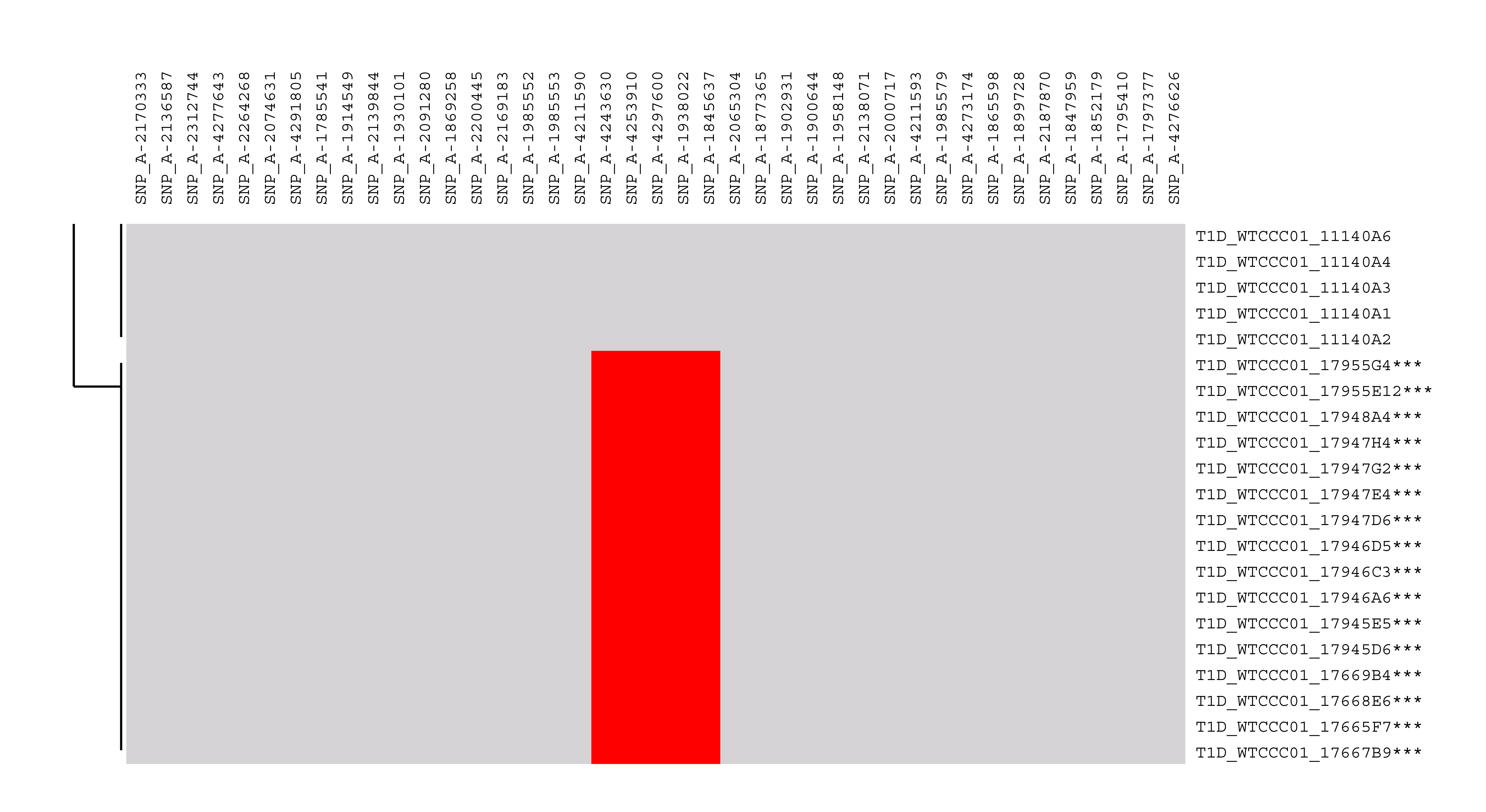


**H**


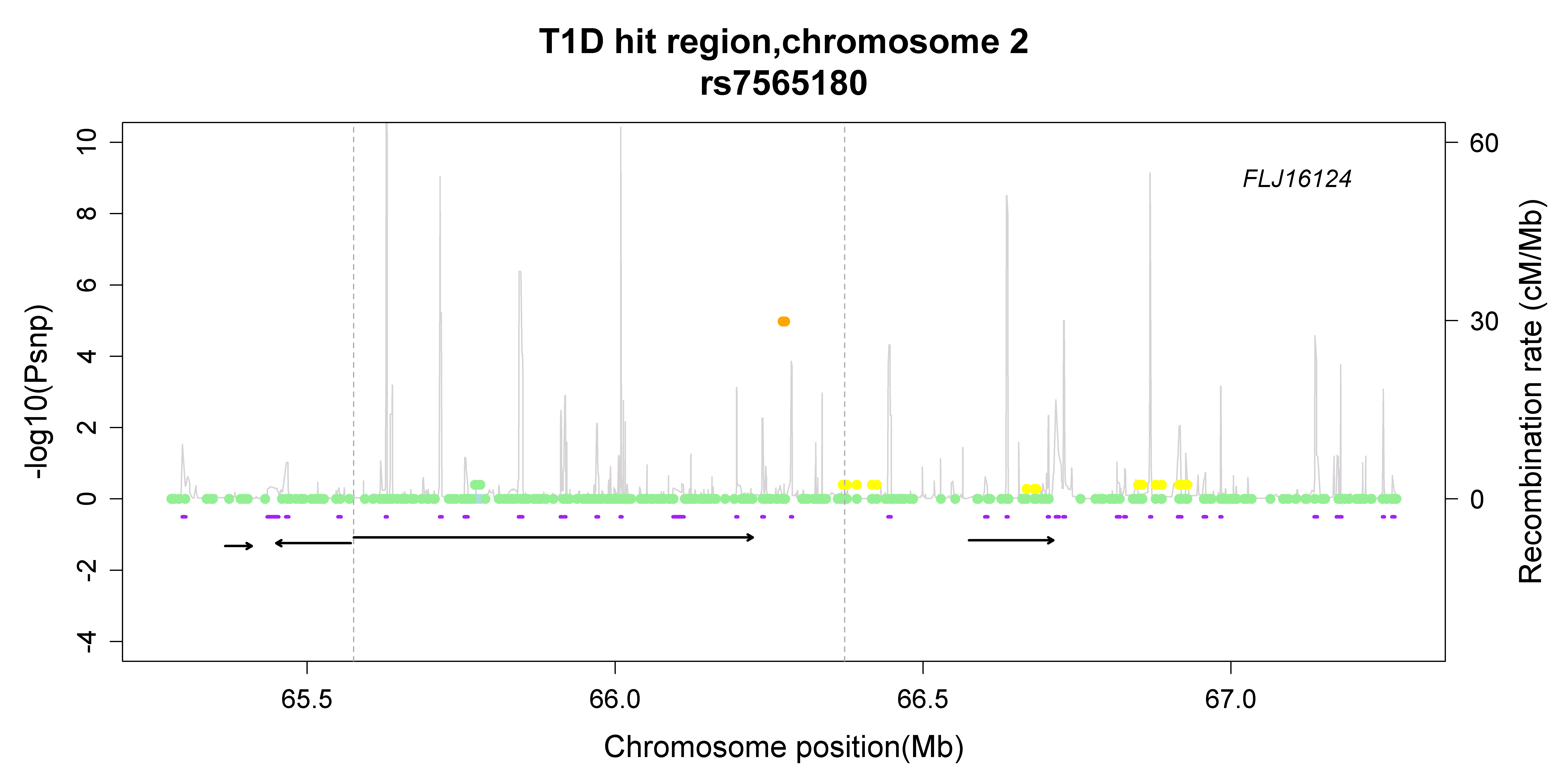


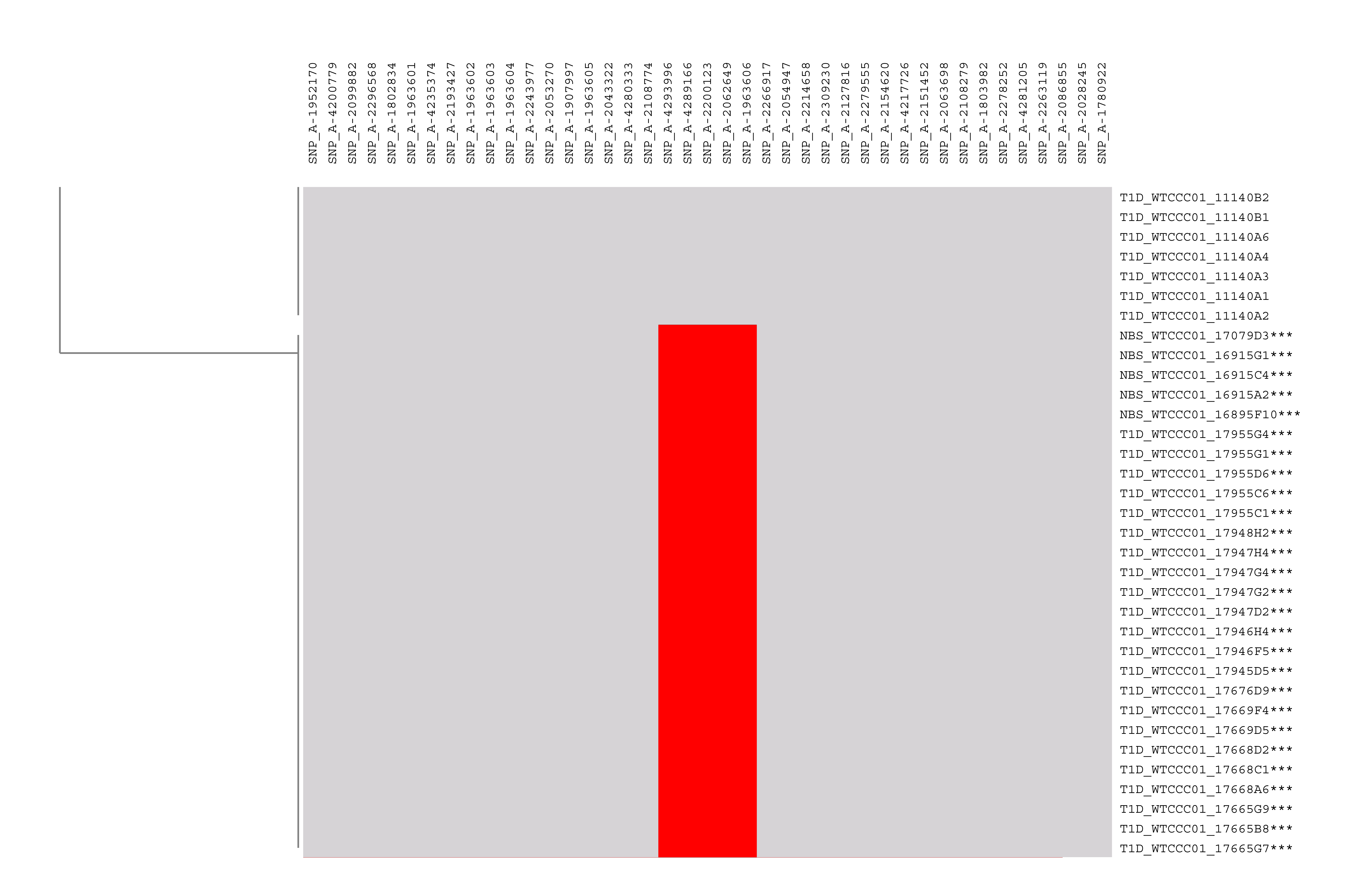


**I**


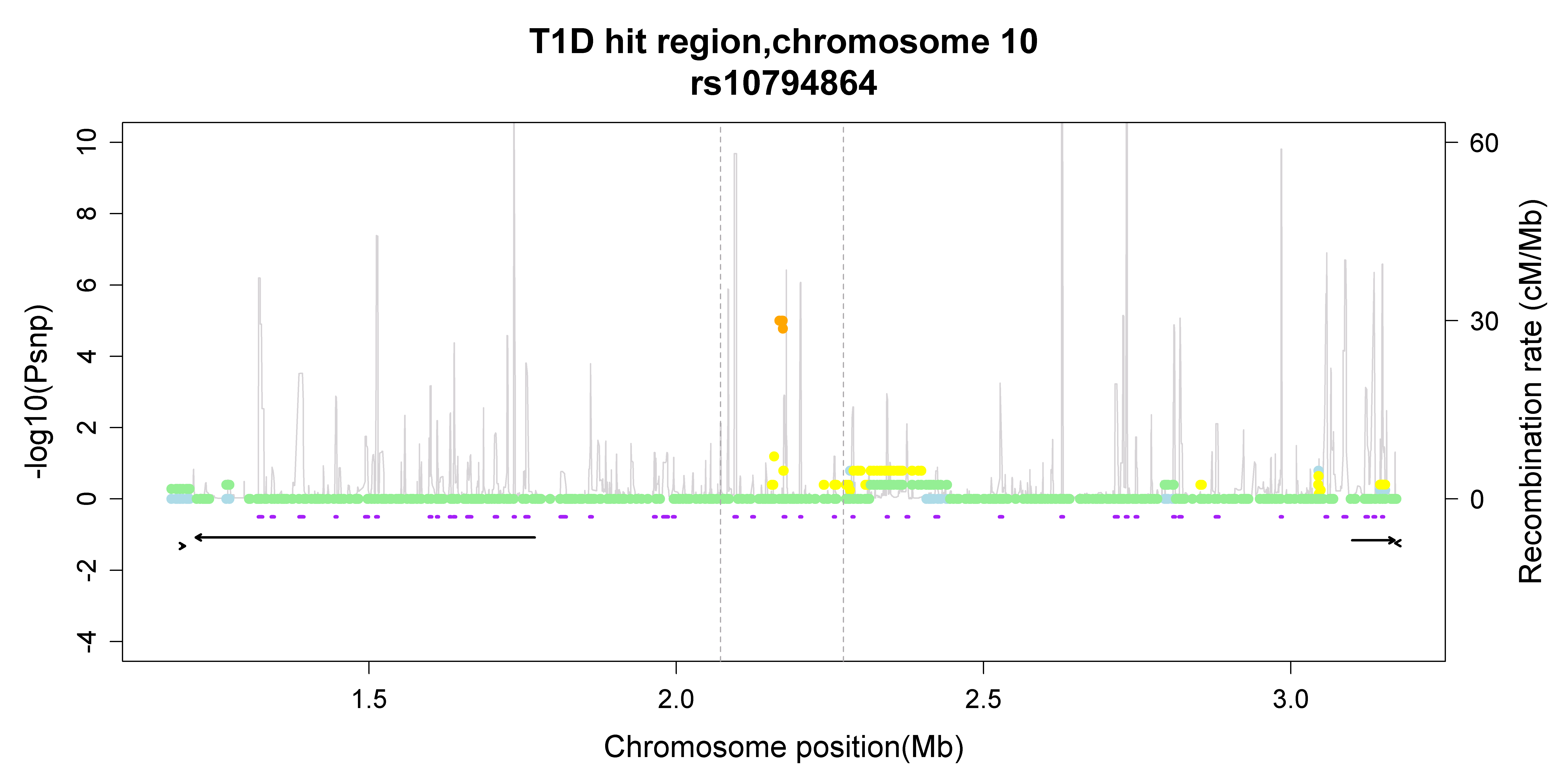


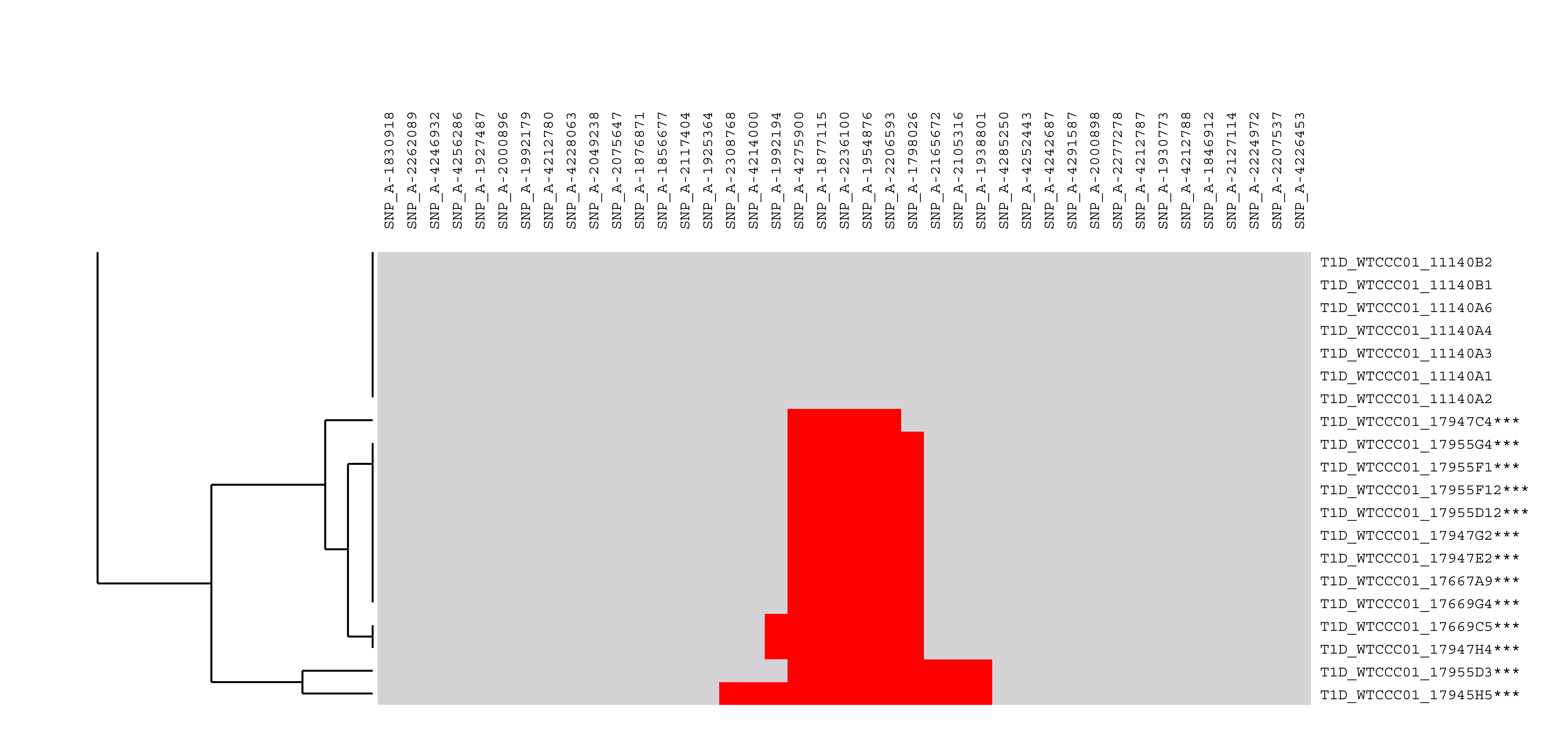


**J**


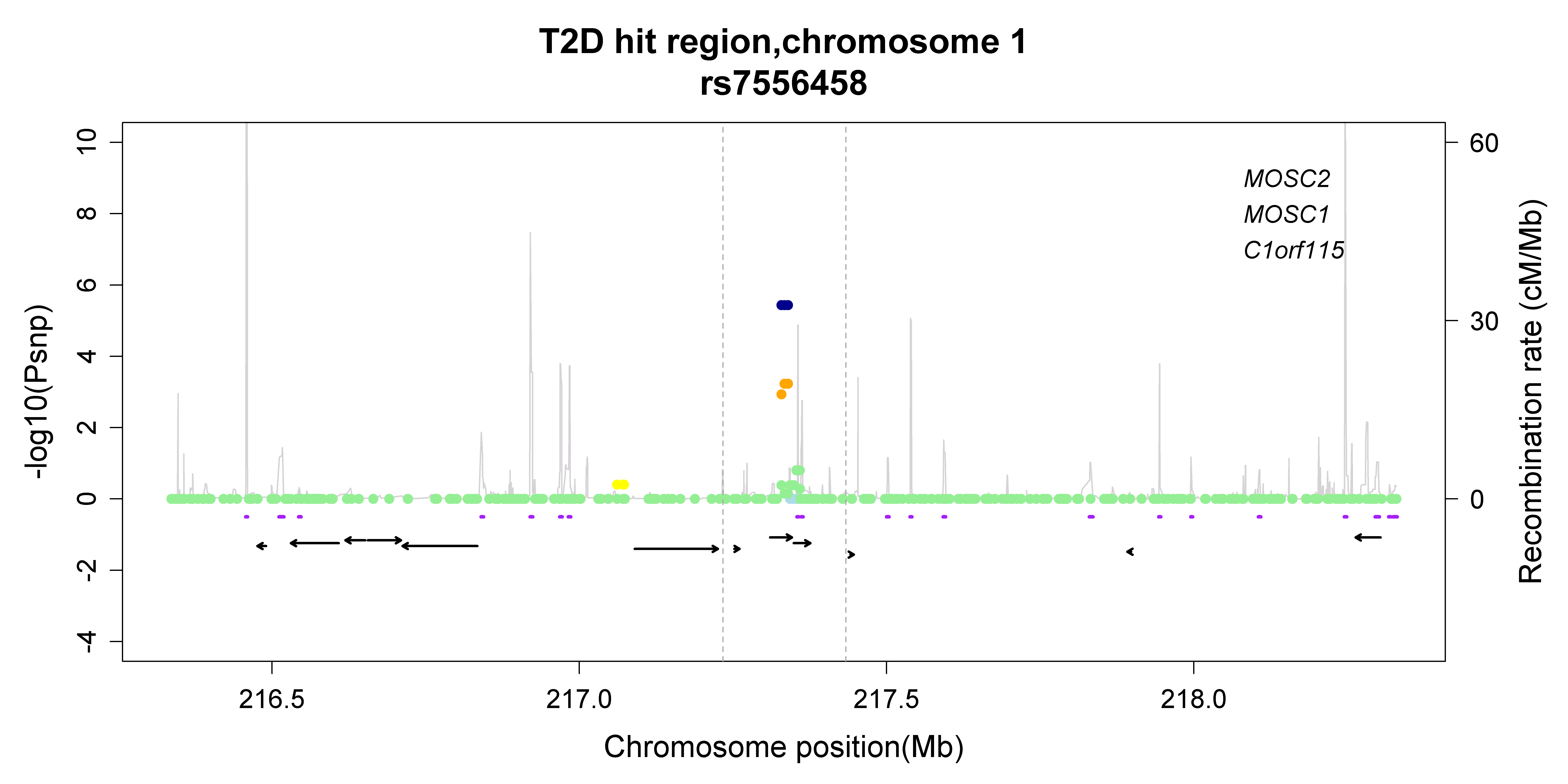


**
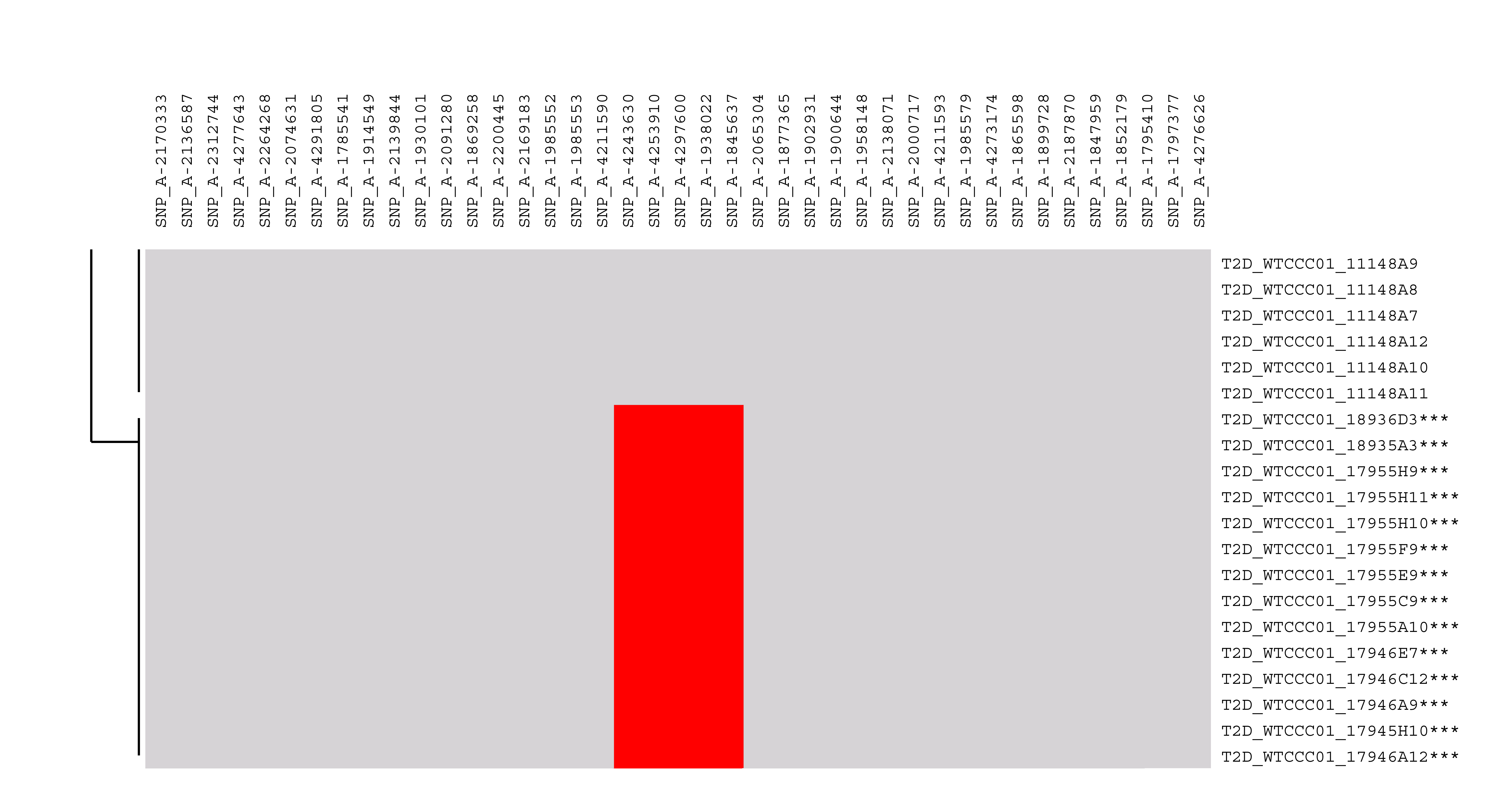
**

**K**

**
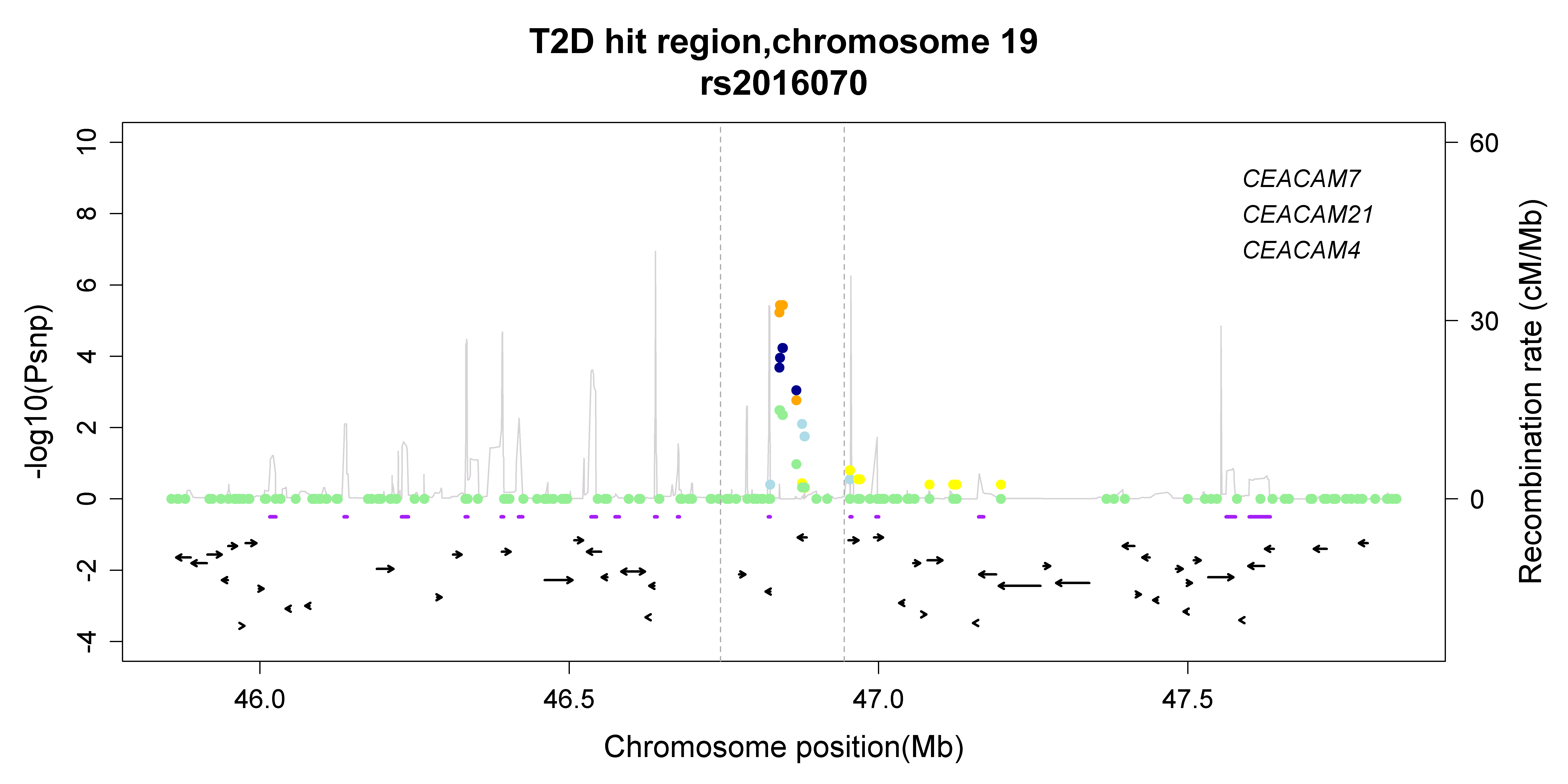
**


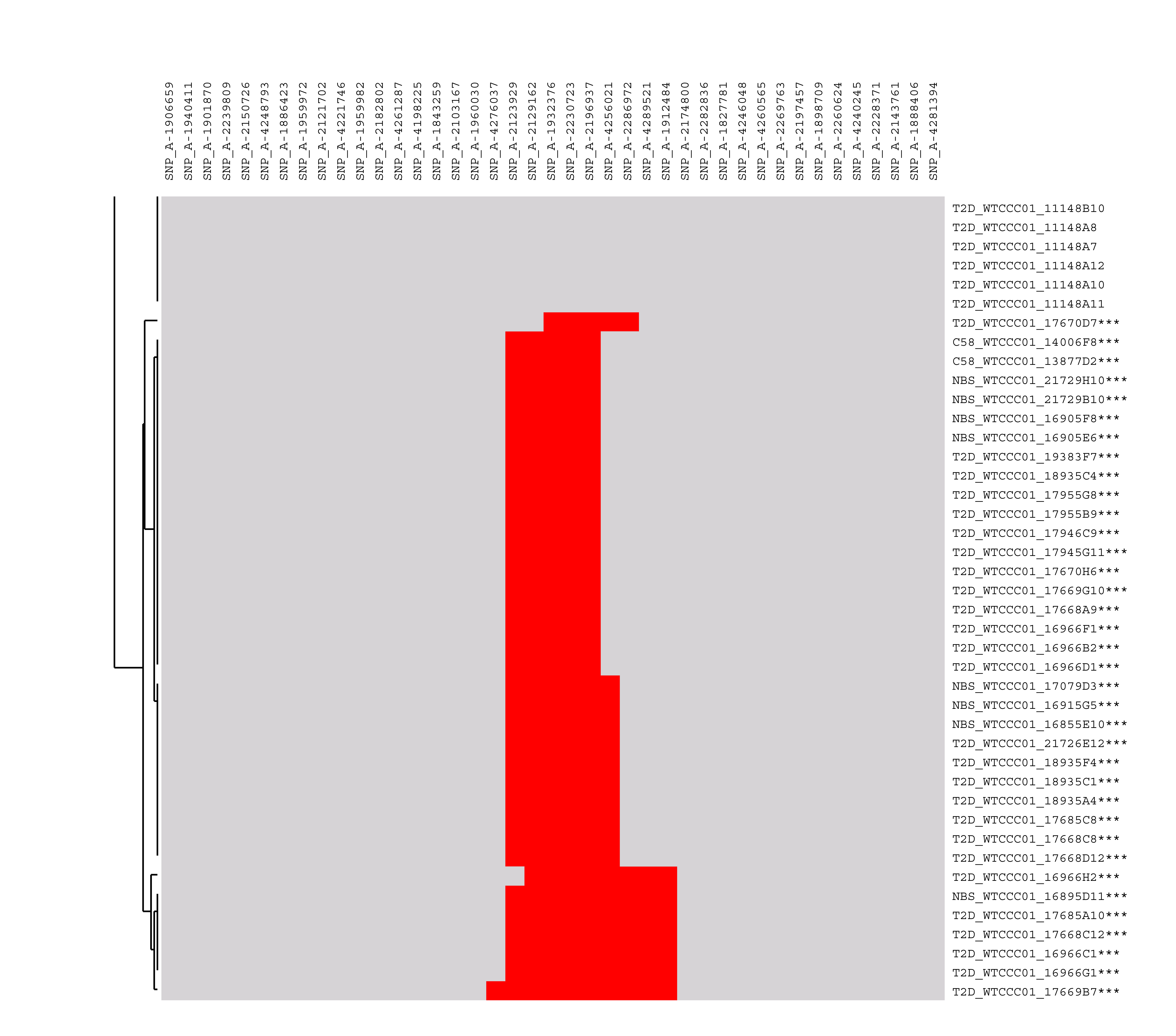

Supplement: Figure S4 — Selected CNV-loci that show strong evidence of association with diseases. The −log10 of the SNP site-based P values are plotted against the genomic location, in which the SNPs that passed the window-based testing are indicated in dark blue for the deletion hypothesis, dark green for the amplification hypothesis and orange for the deletion and amplification hypothesis. SNPs that lacked significance are shown in light colors (light blue for deletion, light green for amplification and yellow for both). Functionally affected regions were characterized within a 0.2 Mb region centered on the identified SNP sites, and the region boundary (vertical dashed line) coincided with the length limitations or the location of neighboring genes. The clustering heat map for 41 SNP windows (each corresponding to the upper CNV-region) demonstrated good CNV boundaries in and around the “first class node.” In the heat map, black indicates a copy number of 0, red a copy number of 1, light grey a copy number of 2 and green a copy number of 3. (6.21 MB DOC) [file pone.0012185.s004.doc]

**
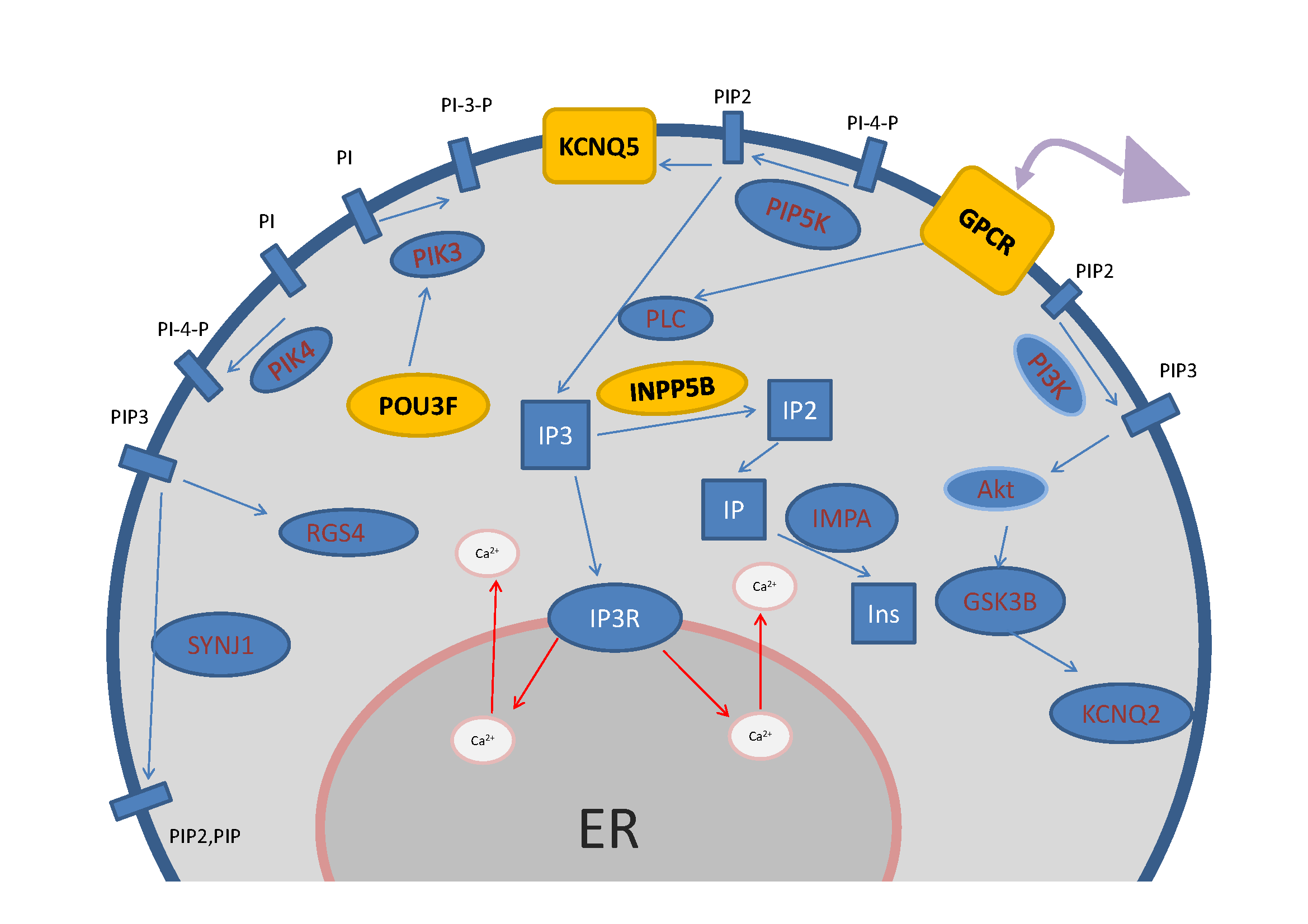
**

Supplement: Figure S5 — The cartoon depicts the function of the calcium-related pathway in bipolar disorder. The Ca2+/IP3 pathway has been reported to be closely related to bipolar disorder, and the molecules revealed in previous studies are labeled with blue circles and in red font. IP3 precursors in the membrane and metabolites in the cytosol are denoted by different shaped boxes. INPP5B, POU3F, Olfactory receptors (belonging to GPCR, G Protein-Coupled Receptors) and KCNQ5, which were found to be associated to CNVs in our work, are labeled with black font and orange circles (or boxes). (0.09 MB DOC) [file pone.0012185.s005.doc]
