# Supplementary material for: Novel Association Strategy with Copy Number Variation for Identifying New Risk Loci of Human Diseases
Source: PLoS One. 2010 Aug 20;5(8):e12185. doi: 10.1371/journal.pone.0012185 (PMC2924882; doi:10.1371/journal.pone.0012185)
Supplement: Table S1 — List of SNP sites showing significance in the window-based testing. (0.63 MB DOC) [file pone.0012185.s006.doc]

| **CNV-landmark** | | | | **CNV-association *P* value** | | | | | | | **Genotype-association *P* value** | |
| --- | --- | --- | --- | --- | --- | --- | --- | --- | --- | --- | --- | --- |
| **Disease** | **Chromosome** | **region(MB)** | **SNP** | **Loss** | **Abnm** | **Gain** | **Window-**  **based** | **Pearson** | **Armitage** | **Armitage exact** | **Trend** | **Genotypic** |
| BD | 1p34.3 | 34.3-39.8 | rs16824514 | 7.55×10^-5^ | 1.06×10^-4^ | 3.83×10^-1^ | 1.16×10^-5^ | 6.23×10^-5^ | 4.38×10^-3^ | 6.31×10^-3^ | 1.58×10^-1^ | 1.42×10^-1^ |
| BD | 1p34.3 | 34.3-39.8 | rs16824518 | 7.55×10^-5^ | 3.29×10^-4^ | 6.83×10^-1^ | 1.16×10^-5^ | 9.37×10^-5^ | 3.12×10^-3^ | 3.60×10^-3^ | 3.59×10^-1^ | 4.54×10^-1^ |
| BD | 1p34.3 | 34.3-39.8 | rs899743 | 7.55×10^-5^ | 3.29×10^-4^ | 6.83×10^-1^ | 1.16×10^-5^ | 9.37×10^-5^ | 3.12×10^-3^ | 3.60×10^-3^ | 8.43×10^-1^ | 6.72×10^-1^ |
| BD | 1p34.3 | 34.3-39.8 | rs1050257 | 7.55×10^-5^ | 3.29×10^-4^ | 6.83×10^-1^ | 1.16×10^-5^ | 9.37×10^-5^ | 3.12×10^-3^ | 3.60×10^-3^ | 7.26×10^-1^ | 5.02×10^-1^ |
| BD | 1p34.3 | 34.3-39.8 | rs6660034 | 3.43×10^-3^ | 9.89×10^-2^ | 2.87×10^-1^ | 1.16×10^-5^ | 9.87×10^-4^ | 2.79×10^-3^ | 7.86×10^-4^ | 7.73×10^-1^ | 8.04×10^-1^ |
| BD | 1p34.3 | 34.3-39.8 | rs10890238 | 3.43×10^-3^ | 9.89×10^-2^ | 2.87×10^-1^ | 1.16×10^-5^ | 9.87×10^-4^ | 2.79×10^-3^ | 7.86×10^-4^ | 4.64×10^-1^ | 7.03×10^-1^ |
| BD | 6q13 | 70-75.9 | rs7740791 | 1.06×10^-3^ | 1.06×10^-3^ | 1.00 | 2.29×10^-4^ | 2.03×10^-3^ | 2.12×10^-3^ | 1.41×10^-3^ | 5.58×10^-1^ | 7.47×10^-1^ |
| BD | 6q13 | 70-75.9 | rs6929988 | 1.06×10^-3^ | 1.06×10^-3^ | 1.00 | 2.29×10^-4^ | 2.03×10^-3^ | 2.12×10^-3^ | 1.41×10^-3^ | 2.01×10^-1^ | 3.63×10^-1^ |
| BD | 6q13 | 70-75.9 | rs4991400 | 2.29×10^-4^ | 2.29×10^-4^ | 1.00 | 2.29×10^-4^ | 5.39×10^-4^ | 4.94×10^-4^ | 3.10×10^-4^ | 6.73×10^-1^ | 8.78×10^-1^ |
| BD | 6q13 | 70-75.9 | rs6453655 | 2.29×10^-4^ | 2.29×10^-4^ | 1.00 | 2.29×10^-4^ | 5.39×10^-4^ | 4.94×10^-4^ | 3.10×10^-4^ | 4.03×10^-1^ | 7.04×10^-1^ |
| BD | 6q13 | 70-75.9 | rs12173399 | 4.68×10^-3^ | 4.68×10^-3^ | 1.00 | 2.29×10^-4^ | 4.68×10^-3^ | 7.14×10^-3^ | 4.68×10^-3^ | 3.69×10^-3^ | 1.27×10^-2^ |
| BD | 12p11.22 | 27.7-30.6 | rs10843150 | 1.60×10^-5^ | 4.90×10^-5^ | 1.00 | 1.60×10^-5^ | 3.33×10^-5^ | 2.54×10^-5^ | 1.42×10^-5^ | 8.26×10^-1^ | 9.59×10^-1^ |
| BD | 12p11.22 | 27.7-30.6 | rs11049519 | 1.60×10^-5^ | 4.90×10^-5^ | 1.00 | 1.60×10^-5^ | 3.33×10^-5^ | 2.54×10^-5^ | 1.42×10^-5^ | 7.03×10^-1^ | 6.90×10^-1^ |
| BD | 12p11.22 | 27.7-30.6 | rs3825246 | 1.60×10^-5^ | 4.90×10^-5^ | 1.00 | 1.60×10^-5^ | 3.33×10^-5^ | 2.54×10^-5^ | 1.42×10^-5^ | 9.34×10^-1^ | 9.54×10^-1^ |
| BD | 12p11.22 | 27.7-30.6 | rs2061759 | 1.60×10^-5^ | 4.90×10^-5^ | 1.00 | 1.60×10^-5^ | 3.33×10^-5^ | 2.54×10^-5^ | 1.42×10^-5^ | NA | NA |
| BD | 12p11.22 | 27.7-30.6 | rs11049520 | 1.60×10^-5^ | 4.90×10^-5^ | 1.00 | 1.60×10^-5^ | 3.33×10^-5^ | 2.54×10^-5^ | 1.42×10^-5^ | 9.90×10^-1^ | 8.35×10^-1^ |
| BD | 12p11.22 | 27.7-30.6 | rs9645730 | 7.55×10^-5^ | 3.17×10^-4^ | 1.00 | 1.60×10^-5^ | 9.72×10^-5^ | 1.66×10^-4^ | 6.13×10^-5^ | 7.84×10^-1^ | 9.36×10^-1^ |
| BD | 12p11.21 | 30.6-33.2 | rs4931443 | 1.00 | 2.98×10^-5^ | 2.98×10^-5^ | 1.36×10^-5^ | 1.49×10^-5^ | 3.61×10^-5^ | 2.31×10^-5^ | NA | NA |
| BD | 12p11.21 | 30.6-33.2 | SNP_A-4239261 | 1.00 | 5.74×10^-5^ | 5.74×10^-5^ | 2.57×10^-5^ | 4.17×10^-5^ | 6.84×10^-5^ | 7.12×10^-5^ | NA | NA |
| BD | 12p11.21 | 30.6-33.2 | rs10843891 | 1.00 | 5.96×10^-4^ | 5.96×10^-4^ | 1.36×10^-5^ | 5.24×10^-4^ | 6.75×10^-4^ | 7.86×10^-4^ | 4.48×10^-1^ | 7.06×10^-1^ |
| BD | 12p11.21 | 30.6-33.2 | rs7971718 | 1.00 | 3.89×10^-4^ | 3.89×10^-4^ | 6.55×10^-5^ | 3.42×10^-4^ | 4.34×10^-4^ | 5.04×10^-4^ | 9.00×10^-2^ | 2.20×10^-1^ |
| BD | 12p11.21 | 30.6-33.2 | rs11051298 | 1.00 | 8.47×10^-4^ | 8.47×10^-4^ | 6.55×10^-5^ | 7.03×10^-4^ | 9.30×10^-4^ | 9.62×10^-4^ | 1.83×10^-1^ | 1.83×10^-1^ |
| BD | 12p11.21 | 30.6-33.2 | rs7969574 | 1.00 | 2.10×10^-5^ | 2.10×10^-5^ | 7.10×10^-5^ | 1.63×10^-5^ | 2.38×10^-5^ | 2.54×10^-5^ | NA | NA |
| BD | 12p11.21 | 30.6-33.2 | rs7136266 | 1.00 | 9.24×10^-6^ | 9.24×10^-6^ | 4.69×10^-5^ | 6.40×10^-6^ | 1.05×10^-5^ | 9.94×10^-6^ | 4.95×10^-1^ | 4.95×10^-1^ |
| BD | 12p11.21 | 30.6-33.2 | SNP_A-2047590 | 1.00 | 2.82×10^-5^ | 2.82×10^-5^ | 4.69×10^-5^ | 2.14×10^-5^ | 3.10×10^-5^ | 3.07×10^-5^ | 1.40×10^-2^ | 1.40×10^-2^ |
| BD | 12p11.21 | 30.6-33.2 | rs7960884 | 1.00 | 1.15×10^-3^ | 1.15×10^-3^ | 4.69×10^-5^ | 1.05×10^-3^ | 1.20×10^-3^ | 1.30×10^-3^ | 7.13×10^-1^ | 5.52×10^-1^ |
| BD | 12p11.21 | 30.6-33.2 | rs7960946 | 1.00 | 1.33×10^-3^ | 1.33×10^-3^ | 6.46×10^-5^ | 1.35×10^-3^ | 1.37×10^-3^ | 1.35×10^-3^ | 1.86×10^-1^ | 2.98×10^-1^ |
| BD | 14q11.2 | 19-24.3 | rs1780934 | 1.00 | 1.51×10^-3^ | 1.51×10^-3^ | 5.57×10^-5^ | 1.47×10^-3^ | 1.52×10^-3^ | 1.70×10^-3^ | NA | NA |
| BD | 14q11.2 | 19-24.3 | rs1319956 | 1.00 | 1.51×10^-3^ | 1.51×10^-3^ | 2.30×10^-4^ | 1.47×10^-3^ | 1.52×10^-3^ | 1.70×10^-3^ | NA | NA |
| BD | 14q11.2 | 19-24.3 | rs1319954 | 1.00 | 1.44×10^-3^ | 1.44×10^-3^ | 8.39×10^-5^ | 1.42×10^-3^ | 1.45×10^-3^ | 1.64×10^-3^ | NA | NA |
| BD | 14q11.2 | 19-24.3 | rs1780930 | 1.00 | 1.29×10^-3^ | 1.29×10^-3^ | 6.69×10^-5^ | 1.23×10^-3^ | 1.30×10^-3^ | 1.42×10^-3^ | NA | NA |
| BD | 14q11.2 | 19-24.3 | rs8013148 | 3.89×10^-1^ | 3.52×10^-4^ | 2.84×10^-4^ | 6.69×10^-5^ | 2.00×10^-4^ | 2.37×10^-4^ | 2.46×10^-4^ | NA | NA |
| BD | 14q11.2 | 19-24.3 | rs1780927 | 3.89×10^-1^ | 2.55×10^-4^ | 2.05×10^-4^ | 1.33×10^-4^ | 1.45×10^-4^ | 1.70×10^-4^ | 1.80×10^-4^ | NA | NA |
| BD | 14q11.2 | 19-24.3 | rs1780909 | 1.00 | 7.08×10^-4^ | 6.47×10^-4^ | 1.33×10^-4^ | 8.73×10^-4^ | 6.20×10^-4^ | 6.20×10^-4^ | NA | NA |
| BD | 14q11.2 | 19-24.3 | rs2318498 | 1.00 | 5.08×10^-5^ | 4.52×10^-5^ | 4.27×10^-5^ | 5.47×10^-5^ | 4.32×10^-5^ | 4.56×10^-5^ | 1.37×10^-1^ | 2.83×10^-1^ |
| BD | 14q11.2 | 19-24.3 | rs4359352 | 1.00 | 5.08×10^-5^ | 4.52×10^-5^ | 1.91×10^-4^ | 5.47×10^-5^ | 4.32×10^-5^ | 4.56×10^-5^ | NA | NA |
| BD | 14q11.2 | 19-24.3 | rs1632089 | 1.00 | 3.06×10^-5^ | 3.14×10^-5^ | 3.73×10^-5^ | 4.84×10^-5^ | 3.59×10^-5^ | 3.78×10^-5^ | NA | NA |
| BD | 14q11.2 | 19-24.3 | rs17242341 | 4.15×10^-1^ | 3.58×10^-5^ | 5.57×10^-5^ | 1.73×10^-5^ | 6.38×10^-5^ | 1.04×10^-4^ | 1.14×10^-4^ | NA | NA |
| BD | 14q11.2 | 19-24.3 | rs1686588 | 4.15×10^-1^ | 3.58×10^-5^ | 5.57×10^-5^ | 2.81×10^-5^ | 6.38×10^-5^ | 1.04×10^-4^ | 1.14×10^-4^ | NA | NA |
| BD | 14q11.2 | 19-24.3 | rs2635556 | 3.34×10^-1^ | 7.55×10^-5^ | 1.36×10^-4^ | 1.58×10^-5^ | 2.23×10^-4^ | 3.18×10^-4^ | 3.70×10^-4^ | NA | NA |
| BD | 14q11.2 | 19-24.3 | rs7146334 | 5.45×10^-1^ | 8.19×10^-5^ | 1.16×10^-4^ | 3.28×10^-5^ | 2.60×10^-4^ | 2.24×10^-4^ | 2.27×10^-4^ | NA | NA |
| BD | 14q11.2 | 19-24.3 | rs6572904 | 5.45×10^-1^ | 2.00×10^-4^ | 2.79×10^-4^ | 4.23×10^-5^ | 6.29×10^-4^ | 5.18×10^-4^ | 5.28×10^-4^ | NA | NA |
| BD | 14q11.2 | 19-24.3 | rs2792111 | 5.45×10^-1^ | 1.96×10^-4^ | 2.74×10^-4^ | 3.28×10^-5^ | 6.11×10^-4^ | 5.12×10^-4^ | 5.62×10^-4^ | NA | NA |
| CAD | 3p26.1 | 4-8.2 | rs163968 | 2.01×10^-4^ | 2.01×10^-4^ | 1.00 | 2.01×10^-4^ | 4.25×10^-5^ | 3.81×10^-4^ | 1.54×10^-4^ | 9.16×10^-1^ | 8.46×10^-1^ |
| CAD | 3p26.1 | 4-8.2 | rs345224 | 2.01×10^-4^ | 2.01×10^-4^ | 1.00 | 2.01×10^-4^ | 4.25×10^-5^ | 3.81×10^-4^ | 1.54×10^-4^ | 8.36×10^-1^ | 6.82×10^-1^ |
| CAD | 3p26.1 | 4-8.2 | rs171121 | 2.01×10^-4^ | 2.01×10^-4^ | 1.00 | 2.01×10^-4^ | 4.25×10^-5^ | 3.81×10^-4^ | 1.54×10^-4^ | 7.58×10^-1^ | 4.68×10^-1^ |
| CAD | 3p26.1 | 4-8.2 | rs7612734 | 2.01×10^-4^ | 2.01×10^-4^ | 1.00 | 2.01×10^-4^ | 4.25×10^-5^ | 3.81×10^-4^ | 1.54×10^-4^ | 5.17×10^-1^ | 8.08×10^-1^ |
| CAD | 3p26.1 | 4-8.2 | rs1356227 | 2.01×10^-4^ | 2.01×10^-4^ | 1.00 | 2.01×10^-4^ | 4.25×10^-5^ | 3.81×10^-4^ | 1.54×10^-4^ | 4.73×10^-1^ | 7.72×10^-1^ |
| CAD | 7q21.11 | 77.2-85.9 | rs10245061 | 4.85×10^-8^ | 4.85×10^-8^ | 1.00 | 4.85×10^-8^ | 1.97×10^-10^ | 9.35×10^-8^ | 3.21×10^-8^ | 2.62×10^-1^ | 2.10×10^-1^ |
| CAD | 7q21.11 | 77.2-85.9 | rs10274362 | 4.85×10^-8^ | 4.85×10^-8^ | 1.00 | 4.85×10^-8^ | 1.97×10^-10^ | 9.35×10^-8^ | 3.21×10^-8^ | 2.81×10^-1^ | 2.31×10^-1^ |
| CAD | 7q21.11 | 77.2-85.9 | rs10248181 | 4.85×10^-8^ | 4.85×10^-8^ | 1.00 | 4.85×10^-8^ | 1.97×10^-10^ | 9.35×10^-8^ | 3.21×10^-8^ | 2.72×10^-1^ | 2.02×10^-1^ |
| CAD | 7q21.11 | 77.2-85.9 | rs10248462 | 4.85×10^-8^ | 4.85×10^-8^ | 1.00 | 4.85×10^-8^ | 1.97×10^-10^ | 9.35×10^-8^ | 3.21×10^-8^ | 2.74×10^-1^ | 2.07×10^-1^ |
| CAD | 16q22.1 | 63.9-69.4 | rs2303200 | 1.64×10^-2^ | 3.93×10^-4^ | 8.98×10^-7^ | 8.98×10^-7^ | 1.94×10^-8^ | 3.98×10^-8^ | 2.49×10^-8^ | NA | NA |
| CAD | 16q22.1 | 63.9-69.4 | rs2287980 | 1.64×10^-2^ | 3.93×10^-4^ | 8.98×10^-7^ | 8.98×10^-7^ | 1.94×10^-8^ | 3.98×10^-8^ | 2.49×10^-8^ | NA | NA |
| CAD | 16q22.1 | 63.9-69.4 | rs4985405 | 1.64×10^-2^ | 3.93×10^-4^ | 8.98×10^-7^ | 8.98×10^-7^ | 1.94×10^-8^ | 3.98×10^-8^ | 2.49×10^-8^ | NA | NA |
| CAD | 19q13.2 | 43.4-48.1 | rs1015758 | 1.51×10^-1^ | 1.54×10^-2^ | 2.77×10^-5^ | 1.39×10^-5^ | 5.64×10^-5^ | 2.98×10^-5^ | 2.00×10^-5^ | NA | NA |
| CAD | 19q13.2 | 43.4-48.1 | rs2109073 | 1.51×10^-1^ | 1.54×10^-2^ | 2.77×10^-5^ | 1.39×10^-5^ | 5.64×10^-5^ | 2.98×10^-5^ | 2.00×10^-5^ | 6.67×10^-1^ | 3.04×10^-2^ |
| CAD | 19q13.2 | 43.4-48.1 | rs2016070 | 1.51×10^-1^ | 9.54×10^-3^ | 1.39×10^-5^ | 1.39×10^-5^ | 2.94×10^-5^ | 1.60×10^-5^ | 1.50×10^-5^ | NA | NA |
| CAD | 19q13.2 | 43.4-48.1 | rs4803494 | 1.51×10^-1^ | 9.54×10^-3^ | 1.39×10^-5^ | 1.39×10^-5^ | 2.94×10^-5^ | 1.60×10^-5^ | 1.50×10^-5^ | 8.31×10^-1^ | 4.54×10^-1^ |
| CAD | 19q13.2 | 43.4-48.1 | rs4803495 | 1.51×10^-1^ | 9.54×10^-3^ | 1.39×10^-5^ | 1.39×10^-5^ | 2.94×10^-5^ | 1.60×10^-5^ | 1.50×10^-5^ | NA | NA |
| CD | 19q13.2 | 43.4-48.1 | rs1015758 | 2.31×10^-1^ | 1.94×10^-3^ | 1.00×10^-4^ | 4.88×10^-5^ | 1.81×10^-4^ | 3.29×10^-5^ | 3.92×10^-5^ | NA | NA |
| CD | 19q13.2 | 43.4-48.1 | rs2109073 | 2.31×10^-1^ | 1.94×10^-3^ | 1.00×10^-4^ | 4.88×10^-5^ | 1.81×10^-4^ | 3.29×10^-5^ | 3.92×10^-5^ | 5.53×10^-1^ | 8.15×10^-1^ |
| CD | 19q13.2 | 43.4-48.1 | rs2016070 | 2.31×10^-1^ | 3.65×10^-3^ | 2.40×10^-4^ | 4.88×10^-5^ | 4.33×10^-4^ | 7.80×10^-5^ | 6.59×10^-5^ | NA | NA |
| CD | 19q13.2 | 43.4-48.1 | rs4803494 | 2.31×10^-1^ | 3.65×10^-3^ | 2.40×10^-4^ | 4.88×10^-5^ | 4.33×10^-4^ | 7.80×10^-5^ | 6.59×10^-5^ | 6.81×10^-1^ | 8.75×10^-1^ |
| CD | 19q13.2 | 43.4-48.1 | rs4803495 | 2.31×10^-1^ | 3.65×10^-3^ | 2.40×10^-4^ | 4.88×10^-5^ | 4.33×10^-4^ | 7.80×10^-5^ | 6.59×10^-5^ | NA | NA |
| HT | 1p31.1 | 69.5-84.6 | rs596204 | 2.85×10^-5^ | 2.85×10^-5^ | 1.00 | 2.85×10^-5^ | 3.43×10^-6^ | 5.00×10^-5^ | 2.58×10^-5^ | 2.02×10^-1^ | 6.05×10^-2^ |
| HT | 1p31.1 | 69.5-84.6 | rs491922 | 2.85×10^-5^ | 2.85×10^-5^ | 1.00 | 2.85×10^-5^ | 3.43×10^-6^ | 5.00×10^-5^ | 2.58×10^-5^ | 2.36×10^-1^ | 4.76×10^-2^ |
| HT | 1p31.1 | 69.5-84.6 | rs648614 | 2.85×10^-5^ | 2.85×10^-5^ | 1.00 | 2.85×10^-5^ | 3.43×10^-6^ | 5.00×10^-5^ | 2.58×10^-5^ | 2.37×10^-1^ | 3.67×10^-2^ |
| HT | 1p31.1 | 69.5-84.6 | rs649058 | 2.85×10^-5^ | 2.85×10^-5^ | 1.00 | 2.85×10^-5^ | 3.43×10^-6^ | 5.00×10^-5^ | 2.58×10^-5^ | 2.04×10^-1^ | 1.87×10^-2^ |
| HT | 2q13 | 108.7-113.9 | rs3906021 | 1.52×10^-4^ | 2.03×10^-4^ | 1.00 | 5.36×10^-5^ | 2.46×10^-4^ | 5.88×10^-4^ | 6.58×10^-4^ | 9.52×10^-1^ | 5.78×10^-1^ |
| HT | 2q13 | 108.7-113.9 | rs17017372 | 2.94×10^-4^ | 3.72×10^-4^ | 1.00 | 5.36×10^-5^ | 6.15×10^-4^ | 1.03×10^-3^ | 1.12×10^-3^ | 5.92×10^-1^ | 8.28×10^-1^ |
| HT | 2q13 | 108.7-113.9 | rs11685349 | 2.94×10^-4^ | 3.72×10^-4^ | 1.00 | 5.36×10^-5^ | 6.15×10^-4^ | 1.03×10^-3^ | 1.12×10^-3^ | 2.62×10^-2^ | 2.87×10^-2^ |
| HT | 2q13 | 108.7-113.9 | rs17777974 | 2.94×10^-4^ | 3.72×10^-4^ | 1.00 | 5.36×10^-5^ | 6.15×10^-4^ | 1.03×10^-3^ | 1.12×10^-3^ | 2.87×10^-2^ | 2.98×10^-2^ |
| HT | 2q13 | 108.7-113.9 | rs12619033 | 2.94×10^-4^ | 3.72×10^-4^ | 1.00 | 5.36×10^-5^ | 6.15×10^-4^ | 1.03×10^-3^ | 1.12×10^-3^ | 1.94×10^-2^ | 1.76×10^-2^ |
| HT | 2q13 | 108.7-113.9 | rs12623828 | 2.94×10^-4^ | 3.72×10^-4^ | 1.00 | 5.36×10^-5^ | 6.15×10^-4^ | 1.03×10^-3^ | 1.12×10^-3^ | 2.45×10^-2^ | 3.49×10^-2^ |
| HT | 2q13 | 108.7-113.9 | rs11122893 | 5.55×10^-4^ | 7.13×10^-4^ | 1.00 | 5.36×10^-5^ | 1.18×10^-3^ | 1.97×10^-3^ | 1.90×10^-3^ | 3.14×10^-2^ | 3.66×10^-2^ |
| HT | 5q12.1 | 58.9-63 | rs4260622 | 1.02×10^-3^ | 1.02×10^-3^ | 1.00 | 2.14×10^-4^ | 1.33×10^-3^ | 1.83×10^-3^ | 1.33×10^-3^ | 6.16×10^-1^ | 5.56×10^-1^ |
| HT | 5q12.1 | 58.9-63 | rs4259134 | 3.36×10^-4^ | 3.36×10^-4^ | 1.00 | 2.14×10^-4^ | 6.10×10^-4^ | 6.00×10^-4^ | 4.17×10^-4^ | 1.55×10^-1^ | 2.36×10^-1^ |
| HT | 5q12.1 | 58.9-63 | rs4302532 | 2.14×10^-4^ | 2.14×10^-4^ | 1.00 | 2.14×10^-4^ | 4.25×10^-4^ | 2.98×10^-4^ | 2.72×10^-4^ | 6.65×10^-1^ | 6.79×10^-1^ |
| HT | 5q12.1 | 58.9-63 | rs4326098 | 2.14×10^-4^ | 2.14×10^-4^ | 1.00 | 2.14×10^-4^ | 4.25×10^-4^ | 2.98×10^-4^ | 2.72×10^-4^ | 2.33×10^-1^ | 4.21×10^-1^ |
| HT | 5q12.1 | 58.9-63 | rs4455508 | 2.14×10^-4^ | 2.14×10^-4^ | 1.00 | 2.14×10^-4^ | 4.25×10^-4^ | 2.98×10^-4^ | 2.72×10^-4^ | 8.86×10^-1^ | 9.76×10^-1^ |
| HT | 5q12.1 | 58.9-63 | rs4470713 | 2.14×10^-4^ | 2.14×10^-4^ | 1.00 | 2.14×10^-4^ | 4.25×10^-4^ | 2.98×10^-4^ | 2.72×10^-4^ | 1.21×10^-1^ | 2.43×10^-1^ |
| HT | 5q22.1 | 109.6-111.5 | rs17458866 | 9.70×10^-5^ | 5.08×10^-5^ | 5.20×10^-1^ | 8.98×10^-5^ | 3.64×10^-5^ | 4.38×10^-4^ | 3.88×10^-4^ | 2.20×10^-1^ | 2.41×10^-1^ |
| HT | 5q22.1 | 109.6-111.5 | rs152875 | 5.08×10^-5^ | 2.65×10^-5^ | 5.20×10^-1^ | 8.98×10^-5^ | 1.38×10^-5^ | 2.39×10^-4^ | 1.33×10^-4^ | NA | NA |
| HT | 5q22.1 | 109.6-111.5 | rs251134 | 8.98×10^-5^ | 4.76×10^-5^ | 5.20×10^-1^ | 8.98×10^-5^ | 4.01×10^-5^ | 3.81×10^-4^ | 3.90×10^-4^ | 4.22×10^-1^ | 2.77×10^-1^ |
| HT | 5q22.1 | 109.6-111.5 | rs251133 | 8.98×10^-5^ | 4.76×10^-5^ | 5.20×10^-1^ | 8.98×10^-5^ | 4.01×10^-5^ | 3.81×10^-4^ | 3.90×10^-4^ | 3.78×10^-1^ | 5.03×10^-1^ |
| HT | 5q22.1 | 109.6-111.5 | rs251130 | 8.98×10^-5^ | 4.76×10^-5^ | 5.20×10^-1^ | 8.98×10^-5^ | 4.01×10^-5^ | 3.81×10^-4^ | 3.90×10^-4^ | 4.52×10^-1^ | 7.31×10^-1^ |
| HT | 5q22.1 | 109.6-111.5 | rs251129 | 8.98×10^-5^ | 4.76×10^-5^ | 5.20×10^-1^ | 8.98×10^-5^ | 4.01×10^-5^ | 3.81×10^-4^ | 3.90×10^-4^ | 2.49×10^-1^ | 2.89×10^-1^ |
| HT | 5q22.1 | 109.6-111.5 | rs524203 | 5.08×10^-5^ | 2.65×10^-5^ | 5.20×10^-1^ | 8.98×10^-5^ | 1.38×10^-5^ | 2.39×10^-4^ | 1.33×10^-4^ | 3.30×10^-1^ | 3.31×10^-1^ |
| HT | 5q22.1 | 109.6-111.5 | rs403237 | 2.73×10^-4^ | 2.73×10^-4^ | 1.00 | 8.98×10^-5^ | 1.56×10^-4^ | 3.79×10^-4^ | 3.07×10^-4^ | 7.93×10^-1^ | 8.73×10^-1^ |
| HT | 10p14 | 6.7-12.3 | rs263431 | 2.21×10^-4^ | 2.21×10^-4^ | 1.00 | 1.67×10^-4^ | 3.13×10^-4^ | 3.82×10^-4^ | 2.66×10^-4^ | 1.00 | 1.00 |
| HT | 10p14 | 6.7-12.3 | rs4749477 | 2.21×10^-4^ | 2.21×10^-4^ | 1.00 | 1.67×10^-4^ | 3.13×10^-4^ | 3.82×10^-4^ | 2.66×10^-4^ | 2.40×10^-1^ | 3.73×10^-1^ |
| HT | 10p14 | 6.7-12.3 | rs10752123 | 2.21×10^-4^ | 2.21×10^-4^ | 1.00 | 1.67×10^-4^ | 3.13×10^-4^ | 3.82×10^-4^ | 2.66×10^-4^ | 4.87×10^-1^ | 2.73×10^-1^ |
| HT | 10p14 | 6.7-12.3 | rs10752124 | 2.21×10^-4^ | 2.21×10^-4^ | 1.00 | 1.67×10^-4^ | 3.13×10^-4^ | 3.82×10^-4^ | 2.66×10^-4^ | 5.03×10^-1^ | 2.98×10^-1^ |
| HT | 10p14 | 6.7-12.3 | rs768166 | 2.21×10^-4^ | 2.21×10^-4^ | 1.00 | 1.67×10^-4^ | 3.13×10^-4^ | 3.82×10^-4^ | 2.66×10^-4^ | 3.03×10^-1^ | 5.84×10^-1^ |
| HT | 10p14 | 6.7-12.3 | rs12240458 | 8.47×10^-4^ | 8.47×10^-4^ | 1.00 | 1.67×10^-4^ | 1.10×10^-3^ | 1.38×10^-3^ | 1.10×10^-3^ | 1.80×10^-1^ | 3.36×10^-1^ |
| HT | 10p14 | 6.7-12.3 | rs10905267 | 8.47×10^-4^ | 8.47×10^-4^ | 1.00 | 1.67×10^-4^ | 1.10×10^-3^ | 1.38×10^-3^ | 1.10×10^-3^ | 2.14×10^-1^ | 2.14×10^-1^ |
| HT | 10p14 | 6.7-12.3 | rs2149110 | 3.13×10^-3^ | 3.13×10^-3^ | 1.00 | 1.67×10^-4^ | 5.80×10^-3^ | 4.77×10^-3^ | 3.74×10^-3^ | 7.54×10^-2^ | 1.71×10^-1^ |
| HT | 10q25.3 | 114.9-118.7 | rs2286739 | 1.69×10^-4^ | 1.69×10^-4^ | 1.00 | 1.69×10^-4^ | 1.10×10^-4^ | 2.26×10^-4^ | 2.20×10^-4^ | 1.60×10^-1^ | 3.72×10^-1^ |
| HT | 10q25.3 | 114.9-118.7 | rs2286737 | 1.69×10^-4^ | 1.69×10^-4^ | 1.00 | 1.69×10^-4^ | 1.10×10^-4^ | 2.26×10^-4^ | 2.20×10^-4^ | 2.12×10^-1^ | 4.47×10^-1^ |
| HT | 10q25.3 | 114.9-118.7 | rs7912665 | 1.69×10^-4^ | 1.69×10^-4^ | 1.00 | 1.69×10^-4^ | 1.10×10^-4^ | 2.26×10^-4^ | 2.20×10^-4^ | 2.04×10^-1^ | 4.14×10^-1^ |
| HT | 10q25.3 | 114.9-118.7 | rs17772944 | 1.69×10^-4^ | 1.69×10^-4^ | 1.00 | 1.69×10^-4^ | 1.10×10^-4^ | 2.26×10^-4^ | 2.20×10^-4^ | 4.50×10^-1^ | 7.37×10^-1^ |
| HT | 10q25.3 | 114.9-118.7 | rs3121458 | 1.69×10^-4^ | 1.69×10^-4^ | 1.00 | 1.69×10^-4^ | 1.10×10^-4^ | 2.26×10^-4^ | 2.20×10^-4^ | 1.93×10^-1^ | 3.92×10^-1^ |
| HT | 10q25.3 | 114.9-118.7 | rs2419854 | 1.34×10^-4^ | 1.34×10^-4^ | 1.00 | 1.69×10^-4^ | 6.68×10^-5^ | 1.84×10^-4^ | 1.25×10^-4^ | 2.70×10^-1^ | 5.33×10^-1^ |
| HT | 11q12.2 | 59.8-61.5 | rs175126 | 2.26×10^-3^ | 2.26×10^-3^ | 1.00 | 5.20×10^-5^ | 1.37×10^-3^ | 4.28×10^-3^ | 1.37×10^-3^ | 2.70×10^-1^ | 4.24×10^-1^ |
| HT | 11q12.2 | 59.8-61.5 | rs11601370 | 2.26×10^-3^ | 2.26×10^-3^ | 1.00 | 5.20×10^-5^ | 1.37×10^-3^ | 4.28×10^-3^ | 1.37×10^-3^ | 1.65×10^-1^ | 3.73×10^-1^ |
| RA | 1q23.3 | 157.3-162.3 | rs4657305 | 3.38×10^-3^ | 1.31×10^-3^ | 3.88×10^-1^ | 1.95×10^-4^ | 1.31×10^-3^ | 3.62×10^-2^ | 2.30×10^-2^ | 7.83×10^-1^ | 5.75×10^-1^ |
| RA | 1q23.3 | 157.3-162.3 | rs2033967 | 1.31×10^-3^ | 5.05×10^-4^ | 3.88×10^-1^ | 1.95×10^-4^ | 5.05×10^-4^ | 1.71×10^-2^ | 1.02×10^-2^ | 9.34×10^-1^ | 5.75×10^-1^ |
| RA | 1q23.3 | 157.3-162.3 | rs4657307 | 1.31×10^-3^ | 5.05×10^-4^ | 3.88×10^-1^ | 1.95×10^-4^ | 5.05×10^-4^ | 1.71×10^-2^ | 1.02×10^-2^ | 8.23×10^-1^ | 7.72×10^-1^ |
| RA | 1q23.3 | 157.3-162.3 | rs10917851 | 1.95×10^-4^ | 7.55×10^-5^ | 3.88×10^-1^ | 1.95×10^-4^ | 7.55×10^-5^ | 3.69×10^-3^ | 1.91×10^-3^ | 1.94×10^-1^ | 4.28×10^-1^ |
| RA | 1q23.3 | 157.3-162.3 | rs12564871 | 1.95×10^-4^ | 7.55×10^-5^ | 3.88×10^-1^ | 1.95×10^-4^ | 7.55×10^-5^ | 3.69×10^-3^ | 1.91×10^-3^ | 3.32×10^-1^ | 5.75×10^-1^ |
| RA | 1q23.3 | 157.3-162.3 | rs16859942 | 1.95×10^-4^ | 7.55×10^-5^ | 3.88×10^-1^ | 1.95×10^-4^ | 7.55×10^-5^ | 3.69×10^-3^ | 1.91×10^-3^ | 7.42×10^-1^ | 7.42×10^-1^ |
| RA | 1q23.3 | 157.3-162.3 | rs13374982 | 1.95×10^-4^ | 7.55×10^-5^ | 3.88×10^-1^ | 1.95×10^-4^ | 7.55×10^-5^ | 3.69×10^-3^ | 1.91×10^-3^ | 2.52×10^-1^ | 2.11×10^-1^ |
| RA | 1q23.3 | 157.3-162.3 | rs16859988 | 1.95×10^-4^ | 7.55×10^-5^ | 3.88×10^-1^ | 1.95×10^-4^ | 7.55×10^-5^ | 3.69×10^-3^ | 1.91×10^-3^ | 4.27×10^-1^ | 4.27×10^-1^ |
| RA | 1q23.3 | 157.3-162.3 | rs6671893 | 1.95×10^-4^ | 7.55×10^-5^ | 3.88×10^-1^ | 1.95×10^-4^ | 7.55×10^-5^ | 3.69×10^-3^ | 1.91×10^-3^ | 4.95×10^-1^ | 3.92×10^-1^ |
| RA | 1q23.3 | 157.3-162.3 | rs1348135 | 1.95×10^-4^ | 7.55×10^-5^ | 3.88×10^-1^ | 1.95×10^-4^ | 7.55×10^-5^ | 3.69×10^-3^ | 1.91×10^-3^ | NA | NA |
| RA | 1q23.3 | 157.3-162.3 | rs1016610 | 1.95×10^-4^ | 7.55×10^-5^ | 3.88×10^-1^ | 1.95×10^-4^ | 7.55×10^-5^ | 3.69×10^-3^ | 1.91×10^-3^ | 6.95×10^-1^ | 9.07×10^-1^ |
| RA | 1q23.3 | 157.3-162.3 | rs7553942 | 1.95×10^-4^ | 7.55×10^-5^ | 3.88×10^-1^ | 1.95×10^-4^ | 7.55×10^-5^ | 3.69×10^-3^ | 1.91×10^-3^ | 6.69×10^-1^ | 8.80×10^-1^ |
| RA | 1q23.3 | 157.3-162.3 | rs1441185 | 5.05×10^-4^ | 1.95×10^-4^ | 3.88×10^-1^ | 1.95×10^-4^ | 1.95×10^-4^ | 7.97×10^-3^ | 5.05×10^-4^ | NA | NA |
| RA | 1q23.3 | 157.3-162.3 | rs1441187 | 1.31×10^-3^ | 5.05×10^-4^ | 3.88×10^-1^ | 1.95×10^-4^ | 5.05×10^-4^ | 1.71×10^-2^ | 1.02×10^-2^ | 7.71×10^-1^ | 9.34×10^-1^ |
| RA | 1q23.3 | 157.3-162.3 | rs1479638 | 1.31×10^-3^ | 5.05×10^-4^ | 3.88×10^-1^ | 1.95×10^-4^ | 5.05×10^-4^ | 1.71×10^-2^ | 1.02×10^-2^ | NA | NA |
| RA | 1q23.3 | 157.3-162.3 | rs12031632 | 1.31×10^-3^ | 5.05×10^-4^ | 3.88×10^-1^ | 1.95×10^-4^ | 5.05×10^-4^ | 1.71×10^-2^ | 1.02×10^-2^ | 5.71×10^-1^ | 8.51×10^-1^ |
| RA | 2q31.2 | 177.8-180.4 | rs2303836 | 7.55×10^-5^ | 7.55×10^-5^ | 1.00 | 7.55×10^-5^ | 7.55×10^-5^ | 2.83×10^-4^ | 7.55×10^-5^ | 5.91×10^-2^ | 6.26×10^-3^ |
| RA | 2q31.2 | 177.8-180.4 | rs4894030 | 7.55×10^-5^ | 7.55×10^-5^ | 1.00 | 7.55×10^-5^ | 7.55×10^-5^ | 2.83×10^-4^ | 7.55×10^-5^ | 3.36×10^-1^ | 5.78×10^-1^ |
| RA | 2q31.2 | 177.8-180.4 | rs10930832 | 7.55×10^-5^ | 7.55×10^-5^ | 1.00 | 7.55×10^-5^ | 7.55×10^-5^ | 2.83×10^-4^ | 7.55×10^-5^ | 3.43×10^-1^ | 2.39×10^-1^ |
| RA | 2q31.2 | 177.8-180.4 | rs11892987 | 7.55×10^-5^ | 7.55×10^-5^ | 1.00 | 7.55×10^-5^ | 7.55×10^-5^ | 2.83×10^-4^ | 7.55×10^-5^ | 2.08×10^-1^ | 2.08×10^-1^ |
| RA | 2q31.2 | 177.8-180.4 | rs10497518 | 7.55×10^-5^ | 7.55×10^-5^ | 1.00 | 7.55×10^-5^ | 7.55×10^-5^ | 2.83×10^-4^ | 7.55×10^-5^ | 8.28×10^-1^ | 6.90×10^-1^ |
| RA | 2q31.2 | 177.8-180.4 | rs12614435 | 7.55×10^-5^ | 7.55×10^-5^ | 1.00 | 7.55×10^-5^ | 7.55×10^-5^ | 2.83×10^-4^ | 7.55×10^-5^ | 3.30×10^-2^ | 9.21×10^-2^ |
| RA | 2q31.2 | 177.8-180.4 | rs16866420 | 7.55×10^-5^ | 7.55×10^-5^ | 1.00 | 7.55×10^-5^ | 7.55×10^-5^ | 2.83×10^-4^ | 7.55×10^-5^ | 7.83×10^-3^ | 1.66×10^-2^ |
| RA | 2q31.2 | 177.8-180.4 | rs7590037 | 7.55×10^-5^ | 7.55×10^-5^ | 1.00 | 7.55×10^-5^ | 7.55×10^-5^ | 2.83×10^-4^ | 7.55×10^-5^ | 2.19×10^-1^ | 3.08×10^-1^ |
| RA | 2q31.2 | 177.8-180.4 | rs1569407 | 7.55×10^-5^ | 7.55×10^-5^ | 1.00 | 7.55×10^-5^ | 7.55×10^-5^ | 2.83×10^-4^ | 7.55×10^-5^ | 2.98×10^-1^ | 5.05×10^-1^ |
| RA | 7p21.3 | 7-13.1 | rs1526538 | 7.55×10^-5^ | 5.39×10^-4^ | 1.00 | 1.13×10^-5^ | 7.55×10^-5^ | 2.09×10^-4^ | 4.63×10^-5^ | 4.88×10^-1^ | 2.36×10^-1^ |
| RA | 7p21.3 | 7-13.1 | rs1526520 | 5.05×10^-4^ | 5.05×10^-4^ | 1.00 | 7.26×10^-5^ | 5.05×10^-4^ | 1.47×10^-3^ | 5.05×10^-4^ | 9.40×10^-1^ | 7.70×10^-1^ |
| RA | 7p21.3 | 7-13.1 | rs1467345 | 1.31×10^-3^ | 1.31×10^-3^ | 1.00 | 6.14×10^-6^ | 1.31×10^-3^ | 3.35×10^-3^ | 1.31×10^-3^ | 6.68×10^-1^ | 9.12×10^-1^ |
| RA | 7q21.11 | 77.2-85.9 | rs10245061 | 8.20×10^-8^ | 8.20×10^-8^ | 1.00 | 8.20×10^-8^ | 2.62×10^-10^ | 1.55×10^-7^ | 9.87×10^-8^ | 8.04×10^-1^ | 4.09×10^-1^ |
| RA | 7q21.11 | 77.2-85.9 | rs10274362 | 8.20×10^-8^ | 8.20×10^-8^ | 1.00 | 8.20×10^-8^ | 2.62×10^-10^ | 1.55×10^-7^ | 9.87×10^-8^ | 7.71×10^-1^ | 4.82×10^-1^ |
| RA | 7q21.11 | 77.2-85.9 | rs10248181 | 8.20×10^-8^ | 8.20×10^-8^ | 1.00 | 8.20×10^-8^ | 2.62×10^-10^ | 1.55×10^-7^ | 9.87×10^-8^ | 7.32×10^-1^ | 4.12×10^-1^ |
| RA | 7q21.11 | 77.2-85.9 | rs10248462 | 8.20×10^-8^ | 8.20×10^-8^ | 1.00 | 8.20×10^-8^ | 2.62×10^-10^ | 1.55×10^-7^ | 9.87×10^-8^ | 7.20×10^-1^ | 4.34×10^-1^ |
| RA | 8p11.1 | 43.2-45.2 | rs7460686 | 1.95×10^-4^ | 7.26×10^-5^ | 3.06×10^-1^ | 2.92×10^-5^ | 5.97×10^-5^ | 3.14×10^-2^ | 2.32×10^-2^ | 9.49×10^-1^ | 8.47×10^-1^ |
| RA | 8p11.1 | 43.2-45.2 | rs7459622 | 1.95×10^-4^ | 7.26×10^-5^ | 3.06×10^-1^ | 2.92×10^-5^ | 5.97×10^-5^ | 3.14×10^-2^ | 2.32×10^-2^ | 8.41×10^-1^ | 2.13×10^-1^ |
| RA | 8p11.1 | 43.2-45.2 | rs12550215 | 2.92×10^-5^ | 1.40×10^-5^ | 3.06×10^-1^ | 2.92×10^-5^ | 4.77×10^-6^ | 8.49×10^-3^ | 3.93×10^-3^ | 9.92×10^-1^ | 1.01×10^-1^ |
| RA | 8p11.1 | 43.2-45.2 | rs10958774 | 2.92×10^-5^ | 3.18×10^-5^ | 5.64×10^-1^ | 2.92×10^-5^ | 1.64×10^-5^ | 2.46×10^-3^ | 1.56×10^-3^ | NA | NA |
| RA | 8p11.1 | 43.2-45.2 | rs13276782 | 2.92×10^-5^ | 3.18×10^-5^ | 5.64×10^-1^ | 2.92×10^-5^ | 1.64×10^-5^ | 2.46×10^-3^ | 1.56×10^-3^ | 7.03×10^-1^ | 2.58×10^-1^ |
| RA | 8p11.1 | 43.2-45.2 | rs12682231 | 2.92×10^-5^ | 3.18×10^-5^ | 5.64×10^-1^ | 2.92×10^-5^ | 1.64×10^-5^ | 2.46×10^-3^ | 1.56×10^-3^ | 7.27×10^-1^ | 7.00×10^-2^ |
| RA | 8p11.1 | 43.2-45.2 | rs4131198 | 1.95×10^-4^ | 2.26×10^-4^ | 5.64×10^-1^ | 2.92×10^-5^ | 1.10×10^-4^ | 1.03×10^-2^ | 1.53×10^-3^ | 8.68×10^-1^ | 6.10×10^-1^ |
| RA | 9p23 | 9-14.1 | rs10977614 | 3.38×10^-3^ | 3.38×10^-3^ | 1.00 | 1.95×10^-4^ | 3.38×10^-3^ | 7.65×10^-3^ | 3.38×10^-3^ | 9.69×10^-1^ | 7.40×10^-1^ |
| RA | 9p23 | 9-14.1 | rs4626664 | 7.55×10^-5^ | 7.55×10^-5^ | 1.00 | 1.95×10^-4^ | 7.55×10^-5^ | 2.83×10^-4^ | 7.55×10^-5^ | 6.38×10^-1^ | 8.92×10^-1^ |
| RA | 9p23 | 9-14.1 | rs11792302 | 7.55×10^-5^ | 7.55×10^-5^ | 1.00 | 1.95×10^-4^ | 7.55×10^-5^ | 2.83×10^-4^ | 7.55×10^-5^ | 8.63×10^-1^ | 9.77×10^-1^ |
| RA | 9p23 | 9-14.1 | rs10977624 | 2.92×10^-5^ | 2.92×10^-5^ | 1.00 | 1.95×10^-4^ | 2.92×10^-5^ | 1.24×10^-4^ | 2.92×10^-5^ | 1.86×10^-1^ | 3.96×10^-1^ |
| RA | 9p23 | 9-14.1 | rs10816081 | 2.92×10^-5^ | 2.92×10^-5^ | 1.00 | 1.95×10^-4^ | 2.92×10^-5^ | 1.24×10^-4^ | 2.92×10^-5^ | 4.71×10^-1^ | 7.14×10^-1^ |
| RA | 9p23 | 9-14.1 | rs10816082 | 2.92×10^-5^ | 2.92×10^-5^ | 1.00 | 1.95×10^-4^ | 2.92×10^-5^ | 1.24×10^-4^ | 2.92×10^-5^ | 9.77×10^-1^ | 9.99×10^-1^ |
| RA | 9p23 | 9-14.1 | rs4329321 | 2.92×10^-5^ | 2.92×10^-5^ | 1.00 | 1.95×10^-4^ | 2.92×10^-5^ | 1.24×10^-4^ | 2.92×10^-5^ | 1.67×10^-1^ | 3.56×10^-1^ |
| RA | 9p23 | 9-14.1 | rs17668620 | 1.95×10^-4^ | 1.95×10^-4^ | 1.00 | 1.95×10^-4^ | 1.95×10^-4^ | 6.43×10^-4^ | 1.95×10^-4^ | 2.85×10^-2^ | 5.82×10^-2^ |
| RA | 9p23 | 9-14.1 | rs1412885 | 5.05×10^-4^ | 5.05×10^-4^ | 1.00 | 1.95×10^-4^ | 5.05×10^-4^ | 1.47×10^-3^ | 5.05×10^-4^ | 2.65×10^-2^ | 6.64×10^-2^ |
| RA | 9p23 | 9-14.1 | rs2802278 | 5.05×10^-4^ | 5.05×10^-4^ | 1.00 | 1.95×10^-4^ | 5.05×10^-4^ | 1.47×10^-3^ | 5.05×10^-4^ | 3.97×10^-2^ | 9.22×10^-2^ |
| RA | 15q13.3 | 29-31.4 | rs2926504 | 8.46×10^-7^ | 1.07×10^-5^ | 4.11×10^-1^ | 8.46×10^-7^ | 3.77×10^-6^ | 9.16×10^-4^ | 8.53×10^-4^ | 2.40×10^-1^ | 2.64×10^-1^ |
| RA | 15q13.3 | 29-31.4 | rs17673589 | 8.46×10^-7^ | 1.07×10^-5^ | 4.11×10^-1^ | 8.46×10^-7^ | 3.77×10^-6^ | 9.16×10^-4^ | 8.53×10^-4^ | 2.73×10^-1^ | 5.45×10^-1^ |
| RA | 15q13.3 | 29-31.4 | rs9672615 | 8.46×10^-7^ | 1.07×10^-5^ | 4.11×10^-1^ | 8.46×10^-7^ | 3.77×10^-6^ | 9.16×10^-4^ | 8.53×10^-4^ | 2.53×10^-1^ | 3.40×10^-1^ |
| RA | 16q22.1 | 63.9-69.4 | rs2303200 | 2.75×10^-1^ | 1.18×10^-3^ | 1.05×10^-4^ | 6.67×10^-5^ | 6.21×10^-5^ | 3.97×10^-5^ | 3.84×10^-5^ | NA | NA |
| RA | 16q22.1 | 63.9-69.4 | rs2287980 | 2.75×10^-1^ | 1.18×10^-3^ | 1.05×10^-4^ | 6.67×10^-5^ | 6.21×10^-5^ | 3.97×10^-5^ | 3.84×10^-5^ | NA | NA |
| RA | 16q22.1 | 63.9-69.4 | rs4985405 | 2.75×10^-1^ | 1.18×10^-3^ | 1.05×10^-4^ | 6.67×10^-5^ | 6.21×10^-5^ | 3.97×10^-5^ | 3.84×10^-5^ | NA | NA |
| RA | 19q13.2 | 43.4-48.1 | rs1015758 | 9.17×10^-1^ | 2.24×10^-4^ | 1.21×10^-5^ | 6.19×10^-6^ | 6.88×10^-5^ | 3.76×10^-4^ | 3.88×10^-4^ | NA | NA |
| RA | 19q13.2 | 43.4-48.1 | rs2109073 | 9.17×10^-1^ | 2.24×10^-4^ | 1.21×10^-5^ | 6.19×10^-6^ | 6.88×10^-5^ | 3.76×10^-4^ | 3.88×10^-4^ | 8.95×10^-1^ | 2.58×10^-1^ |
| RA | 19q13.2 | 43.4-48.1 | rs2016070 | 9.17×10^-1^ | 1.23×10^-4^ | 6.19×10^-6^ | 6.19×10^-6^ | 3.63×10^-5^ | 2.09×10^-4^ | 1.55×10^-4^ | NA | NA |
| RA | 19q13.2 | 43.4-48.1 | rs4803494 | 9.17×10^-1^ | 1.23×10^-4^ | 6.19×10^-6^ | 6.19×10^-6^ | 3.63×10^-5^ | 2.09×10^-4^ | 1.55×10^-4^ | 4.89×10^-1^ | 7.76×10^-1^ |
| RA | 19q13.2 | 43.4-48.1 | rs4803495 | 9.17×10^-1^ | 1.23×10^-4^ | 6.19×10^-6^ | 6.19×10^-6^ | 3.63×10^-5^ | 2.09×10^-4^ | 1.55×10^-4^ | NA | NA |
| RA | 19q13.2 | 43.4-48.1 | rs2215284 | 1.00 | 1.18×10^-3^ | 4.87×10^-4^ | 6.19×10^-6^ | 3.71×10^-4^ | 2.76×10^-3^ | 2.95×10^-3^ | 9.00×10^-1^ | 7.29×10^-1^ |
| T1D | 1p36.13 | 15.6-20.2 | SNP_A-1872931 | 1.23×10^-5^ | 8.75×10^-6^ | 1.00 | 8.75×10^-6^ | 2.63×10^-6^ | 4.14×10^-5^ | 3.03×10^-5^ | 5.78×10^-2^ | 1.55×10^-1^ |
| T1D | 1p36.13 | 15.6-20.2 | rs6429753 | 1.23×10^-5^ | 8.75×10^-6^ | 1.00 | 8.75×10^-6^ | 2.63×10^-6^ | 4.14×10^-5^ | 3.03×10^-5^ | 1.16×10^-1^ | 2.08×10^-1^ |
| T1D | 1p36.13 | 15.6-20.2 | rs6429757 | 8.75×10^-6^ | 6.20×10^-6^ | 1.00 | 8.75×10^-6^ | 2.04×10^-6^ | 2.97×10^-5^ | 1.71×10^-5^ | 4.43×10^-1^ | 6.86×10^-1^ |
| T1D | 1p36.13 | 15.6-20.2 | rs6429758 | 8.75×10^-6^ | 6.20×10^-6^ | 1.00 | 8.75×10^-6^ | 2.04×10^-6^ | 2.97×10^-5^ | 1.71×10^-5^ | 7.16×10^-1^ | 9.36×10^-1^ |
| T1D | 1p36.13 | 15.6-20.2 | rs6429759 | 8.75×10^-6^ | 6.20×10^-6^ | 1.00 | 8.75×10^-6^ | 2.04×10^-6^ | 2.97×10^-5^ | 1.71×10^-5^ | 4.72×10^-1^ | 6.89×10^-1^ |
| T1D | 1p36.13 | 15.6-20.2 | rs3856275 | 5.18×10^-4^ | 3.65×10^-4^ | 1.00 | 8.75×10^-6^ | 2.42×10^-4^ | 1.67×10^-3^ | 1.47×10^-3^ | 1.94×10^-1^ | 1.94×10^-1^ |
| T1D | 1p36.13 | 15.6-20.2 | rs16851819 | 5.18×10^-4^ | 3.65×10^-4^ | 1.00 | 8.75×10^-6^ | 2.42×10^-4^ | 1.67×10^-3^ | 1.47×10^-3^ | 3.67×10^-2^ | 6.01×10^-2^ |
| T1D | 1p36.13 | 15.6-20.2 | rs6669417 | 6.58×10^-4^ | 6.58×10^-4^ | 1.00 | 8.75×10^-6^ | 2.72×10^-4^ | 1.11×10^-3^ | 6.21×10^-4^ | 3.74×10^-2^ | 7.07×10^-2^ |
| T1D | 1p36.13 | 15.6-20.2 | rs12385690 | 6.58×10^-4^ | 6.58×10^-4^ | 1.00 | 8.75×10^-6^ | 2.72×10^-4^ | 1.11×10^-3^ | 6.21×10^-4^ | 3.62×10^-2^ | 6.89×10^-2^ |
| T1D | 1q41 | 210.5-219.8 | rs337147 | 9.51×10^-7^ | 8.85×10^-5^ | 1.00 | 9.51×10^-7^ | 5.81×10^-7^ | 8.81×10^-5^ | 7.67×10^-6^ | 5.66×10^-1^ | 6.62×10^-1^ |
| T1D | 1q41 | 210.5-219.8 | rs12026813 | 9.51×10^-7^ | 8.85×10^-5^ | 1.00 | 9.51×10^-7^ | 5.81×10^-7^ | 8.81×10^-5^ | 7.67×10^-6^ | NA | NA |
| T1D | 1q41 | 210.5-219.8 | rs7556458 | 9.51×10^-7^ | 8.85×10^-5^ | 1.00 | 9.51×10^-7^ | 5.81×10^-7^ | 8.81×10^-5^ | 7.67×10^-6^ | NA | NA |
| T1D | 1q41 | 210.5-219.8 | rs11118603 | 9.51×10^-7^ | 8.85×10^-5^ | 1.00 | 9.51×10^-7^ | 5.81×10^-7^ | 8.81×10^-5^ | 7.67×10^-6^ | NA | NA |
| T1D | 1q41 | 210.5-219.8 | rs6657944 | 9.51×10^-7^ | 8.85×10^-5^ | 1.00 | 9.51×10^-7^ | 5.81×10^-7^ | 8.81×10^-5^ | 7.67×10^-6^ | NA | NA |
| T1D | 2p14 | 64-68.5 | rs13409606 | 1.06×10^-5^ | 1.06×10^-5^ | 1.00 | 1.06×10^-5^ | 1.35×10^-5^ | 2.15×10^-5^ | 1.25×10^-5^ | NA | NA |
| T1D | 2p14 | 64-68.5 | rs12464049 | 1.06×10^-5^ | 1.06×10^-5^ | 1.00 | 1.06×10^-5^ | 1.35×10^-5^ | 2.15×10^-5^ | 1.25×10^-5^ | 5.19×10^-1^ | 7.63×10^-1^ |
| T1D | 2p14 | 64-68.5 | rs7565180 | 1.06×10^-5^ | 1.06×10^-5^ | 1.00 | 1.06×10^-5^ | 1.35×10^-5^ | 2.15×10^-5^ | 1.25×10^-5^ | 4.45×10^-1^ | 6.79×10^-1^ |
| T1D | 2p14 | 64-68.5 | rs17031611 | 1.06×10^-5^ | 1.06×10^-5^ | 1.00 | 1.06×10^-5^ | 1.35×10^-5^ | 2.15×10^-5^ | 1.25×10^-5^ | 1.32×10^-1^ | 1.41×10^-1^ |
| T1D | 2p14 | 64-68.5 | rs11888061 | 1.06×10^-5^ | 1.06×10^-5^ | 1.00 | 1.06×10^-5^ | 1.35×10^-5^ | 2.15×10^-5^ | 1.25×10^-5^ | 1.12×10^-1^ | 8.94×10^-2^ |
| T1D | 5q22.1 | 109.6-111.5 | rs17458866 | 3.94×10^-3^ | 2.27×10^-3^ | 5.20×10^-1^ | 1.58×10^-4^ | 3.50×10^-3^ | 1.04×10^-2^ | 1.21×10^-2^ | 9.42×10^-1^ | 6.43×10^-1^ |
| T1D | 5q22.1 | 109.6-111.5 | rs524203 | 5.68×10^-4^ | 3.11×10^-4^ | 5.20×10^-1^ | 1.58×10^-4^ | 3.10×10^-4^ | 1.91×10^-3^ | 1.80×10^-3^ | 8.76×10^-1^ | 9.77×10^-1^ |
| T1D | 5q22.1 | 109.6-111.5 | rs403237 | 2.94×10^-3^ | 2.94×10^-3^ | 1.00 | 1.58×10^-4^ | 2.90×10^-3^ | 3.63×10^-3^ | 2.90×10^-3^ | 8.66×10^-1^ | 8.83×10^-1^ |
| T1D | 10p15.3 | 0-3 | rs2210553 | 1.00×10^-5^ | 1.00×10^-5^ | 1.00 | 1.00×10^-5^ | 6.67×10^-6^ | 3.95×10^-5^ | 6.67×10^-6^ | 5.49×10^-1^ | 3.16×10^-1^ |
| T1D | 10p15.3 | 0-3 | rs11251071 | 1.00×10^-5^ | 1.00×10^-5^ | 1.00 | 1.00×10^-5^ | 6.67×10^-6^ | 3.95×10^-5^ | 6.67×10^-6^ | 9.20×10^-1^ | 9.84×10^-1^ |
| T1D | 10p15.3 | 0-3 | rs10794864 | 1.00×10^-5^ | 1.00×10^-5^ | 1.00 | 1.00×10^-5^ | 6.67×10^-6^ | 3.95×10^-5^ | 6.67×10^-6^ | 9.98×10^-1^ | 9.57×10^-1^ |
| T1D | 10p15.3 | 0-3 | rs10794865 | 1.00×10^-5^ | 1.00×10^-5^ | 1.00 | 1.00×10^-5^ | 6.67×10^-6^ | 3.95×10^-5^ | 6.67×10^-6^ | 9.73×10^-1^ | 9.49×10^-1^ |
| T1D | 10p15.3 | 0-3 | rs7100110 | 1.00×10^-5^ | 1.00×10^-5^ | 1.00 | 1.00×10^-5^ | 6.67×10^-6^ | 3.95×10^-5^ | 6.67×10^-6^ | NA | NA |
| T1D | 10p15.3 | 0-3 | rs10903683 | 1.67×10^-5^ | 1.67×10^-5^ | 1.00 | 1.00×10^-5^ | 1.67×10^-5^ | 8.59×10^-5^ | 1.67×10^-5^ | 9.09×10^-1^ | 9.14×10^-1^ |
| T1D | 14q11.2 | 19-24.3 | rs6572449 | 2.41×10^-7^ | 1.70×10^-7^ | 1.00 | 1.70×10^-7^ | 8.89×10^-9^ | 9.69×10^-7^ | 3.30×10^-7^ | 1.37×10^-4^ | 6.78×10^-4^ |
| T1D | 14q11.2 | 19-24.3 | rs10873018 | 1.70×10^-7^ | 1.20×10^-7^ | 1.00 | 1.70×10^-7^ | 5.16×10^-9^ | 6.88×10^-7^ | 1.58×10^-7^ | 6.26×10^-3^ | 2.00×10^-3^ |
| T1D | 14q11.2 | 19-24.3 | rs10483273 | 1.70×10^-7^ | 1.20×10^-7^ | 1.00 | 1.70×10^-7^ | 5.16×10^-9^ | 6.88×10^-7^ | 1.58×10^-7^ | NA | NA |
| T1D | 14q11.2 | 19-24.3 | rs2204985 | 1.70×10^-7^ | 1.20×10^-7^ | 1.00 | 1.70×10^-7^ | 5.16×10^-9^ | 6.88×10^-7^ | 1.58×10^-7^ | 2.57×10^-3^ | 1.18×10^-3^ |
| T1D | 14q11.2 | 19-24.3 | rs11997 | 1.13×10^-6^ | 1.13×10^-6^ | 1.00 | 1.70×10^-7^ | 3.80×10^-8^ | 2.04×10^-6^ | 2.96×10^-7^ | 3.86×10^-3^ | 1.50×10^-2^ |
| T1D | 14q11.2 | 19-24.3 | rs17198805 | 1.52×10^-3^ | 1.52×10^-3^ | 1.00 | 1.70×10^-7^ | 7.35×10^-4^ | 2.90×10^-3^ | 7.35×10^-4^ | 5.17×10^-3^ | 2.00×10^-2^ |
| T1D | 14q11.2 | 19-24.3 | rs11850502 | 2.19×10^-3^ | 2.19×10^-3^ | 1.00 | 1.70×10^-7^ | 1.37×10^-3^ | 4.17×10^-3^ | 1.37×10^-3^ | 9.69×10^-4^ | 1.45×10^-3^ |
| T1D | 14q11.2 | 19-24.3 | rs10483277 | 2.45×10^-3^ | 2.45×10^-3^ | 1.00 | 1.70×10^-7^ | 2.45×10^-3^ | 8.68×10^-3^ | 4.95×10^-3^ | NA | NA |
| T1D | 15q11.2 | 18.3-23.2 | rs2880332 | 8.88×10^-6^ | 3.29×10^-1^ | 4.74×10^-2^ | 1.13×10^-4^ | 8.77×10^-6^ | 3.90×10^-3^ | 3.94×10^-3^ | NA | NA |
| T2D | 1p34.3 | 34.3-39.8 | rs16824514 | 1.52×10^-3^ | 1.64×10^-2^ | 5.22×10^-1^ | 2.35×10^-4^ | 8.37×10^-4^ | 1.42×10^-3^ | 6.00×10^-4^ | 6.35×10^-1^ | 8.86×10^-1^ |
| T2D | 1p34.3 | 34.3-39.8 | rs16824518 | 1.52×10^-3^ | 2.85×10^-2^ | 2.82×10^-1^ | 2.35×10^-4^ | 4.96×10^-4^ | 1.00×10^-3^ | 3.77×10^-4^ | 6.61×10^-1^ | 8.59×10^-1^ |
| T2D | 1p34.3 | 34.3-39.8 | rs899743 | 1.52×10^-3^ | 2.85×10^-2^ | 2.82×10^-1^ | 2.35×10^-4^ | 4.96×10^-4^ | 1.00×10^-3^ | 3.77×10^-4^ | 2.87×10^-1^ | 3.22×10^-3^ |
| T2D | 1p34.3 | 34.3-39.8 | rs1050257 | 1.52×10^-3^ | 2.85×10^-2^ | 2.82×10^-1^ | 2.35×10^-4^ | 4.96×10^-4^ | 1.00×10^-3^ | 3.77×10^-4^ | 4.54×10^-1^ | 3.69×10^-3^ |
| T2D | 1q41 | 210.5-219.8 | rs337147 | 3.65×10^-6^ | 1.17×10^-3^ | 4.13×10^-1^ | 3.65×10^-6^ | 1.11×10^-6^ | 1.80×10^-5^ | 5.44×10^-7^ | 9.61×10^-1^ | 8.03×10^-1^ |
| T2D | 1q41 | 210.5-219.8 | rs12026813 | 3.65×10^-6^ | 1.17×10^-3^ | 4.13×10^-1^ | 3.65×10^-6^ | 1.11×10^-6^ | 1.80×10^-5^ | 5.44×10^-7^ | NA | NA |
| T2D | 1q41 | 210.5-219.8 | rs7556458 | 3.65×10^-6^ | 5.84×10^-4^ | 7.11×10^-1^ | 3.65×10^-6^ | 1.67×10^-6^ | 8.28×10^-5^ | 2.47×10^-5^ | NA | NA |
| T2D | 1q41 | 210.5-219.8 | rs11118603 | 3.65×10^-6^ | 5.84×10^-4^ | 7.11×10^-1^ | 3.65×10^-6^ | 1.67×10^-6^ | 8.28×10^-5^ | 2.47×10^-5^ | NA | NA |
| T2D | 1q41 | 210.5-219.8 | rs6657944 | 3.65×10^-6^ | 5.84×10^-4^ | 7.11×10^-1^ | 3.65×10^-6^ | 1.67×10^-6^ | 8.28×10^-5^ | 2.47×10^-5^ | NA | NA |
| T2D | 19q13.2 | 43.4-48.1 | rs1015758 | 2.06×10^-4^ | 5.87×10^-6^ | 3.25×10^-3^ | 5.79×10^-5^ | 1.19×10^-5^ | 7.00×10^-1^ | 7.71×10^-1^ | NA | NA |
| T2D | 19q13.2 | 43.4-48.1 | rs2109073 | 1.10×10^-4^ | 3.67×10^-6^ | 3.25×10^-3^ | 5.79×10^-5^ | 6.55×10^-6^ | 7.89×10^-1^ | 8.47×10^-1^ | 1.59×10^-1^ | 8.30×10^-2^ |
| T2D | 19q13.2 | 43.4-48.1 | rs2016070 | 5.79×10^-5^ | 3.64×10^-6^ | 4.44×10^-3^ | 5.79×10^-5^ | 4.73×10^-6^ | 9.11×10^-1^ | 9.08×10^-1^ | NA | NA |
| T2D | 19q13.2 | 43.4-48.1 | rs4803494 | 5.79×10^-5^ | 3.64×10^-6^ | 4.44×10^-3^ | 5.79×10^-5^ | 4.73×10^-6^ | 9.11×10^-1^ | 9.08×10^-1^ | 6.63×10^-1^ | 6.48×10^-1^ |
| T2D | 19q13.2 | 43.4-48.1 | rs4803495 | 5.79×10^-5^ | 3.64×10^-6^ | 4.44×10^-3^ | 5.79×10^-5^ | 4.73×10^-6^ | 9.11×10^-1^ | 9.08×10^-1^ | NA | NA |
| T2D | 19q13.2 | 43.4-48.1 | rs2215284 | 8.97×10^-4^ | 1.72×10^-3^ | 1.05×10^-1^ | 5.79×10^-5^ | 1.05×10^-3^ | 7.08×10^-1^ | 7.47×10^-1^ | 2.43×10^-1^ | 1.30×10^-1^ |
