# Supplementary material for: Novel Association Strategy with Copy Number Variation for Identifying New Risk Loci of Human Diseases
Source: PLoS One. 2010 Aug 20;5(8):e12185. doi: 10.1371/journal.pone.0012185 (PMC2924882; doi:10.1371/journal.pone.0012185)
Supplement: Table S2 — 22 risk genes that were validated from previous studies. (0.20 MB DOC) [file pone.0012185.s007.doc]

| **Disease** | **Gene** | **Chromosome** | **region(MB)** | **SNP** | **Loss** | **Abnm** | **Gain** | **Window-**  **based** | **Comments** |
| --- | --- | --- | --- | --- | --- | --- | --- | --- | --- |
| BD | *CCDC91* | 12p11.22 | 27.7-30.6 | rs10843150 | 1.60×10^-5^ | 4.90×10^-5^ | 1.00 | 1.60×10^-5^ | (i)Involved in the regulation of membrane traffic through the trans-Golgi network (TGN). (ii)Expressed in rat brain, and interact with GGAs to affect protein sorting, and many members in the same pathway associates with bipolar disorder, and its binding partner AP1G1 was up-regulated in the post-mortem cerebellum of schizophrenia patients[1,2].. |
| BD | *INPP5B* | 1p34.3 | 34.3-39.8 | rs16824514 | 7.55×10^-5^ | 1.06×10^-4^ | 3.83×10^-1^ | 1.16×10^-5^ | (i)Hydrolyzes the calcium-mobilizing second messenger inositol 1,4,5-trisphosphate(IP3), may impose direct effect on neurophysiologic regulation[3] this is a signal-terminating reaction,(ii)many gene in the same pathway are reported to be related to bipolar disease[2,4,5,6]. |
| BD | *MTF1* | 1p34.3 | 34.3-39.8 | rs16824514 | 7.55×10^-5^ | 1.06×10^-4^ | 3.83×10^-1^ | 1.16×10^-5^ | (i) MRE-binding transcription factor (ii) Expressed in multiple regions of the brain and CNS, including the amygdala, caudate, corpus callosum, hippocampus, substantia nigra and subthalmic nucleus. (iii) Its binding partner metal responsive element is regulated by lithium (Li) salts in the treatment of bipolar disorders[7]. |
| BD | *Olfactory receptors* | 14q11.2 | 19-24.3 | rs2635556 | 3.34×10^-1^ | 7.55×10^-5^ | 1.36×10^-4^ | 1.58×10^-5^ | (i)Belongs to the G-protein coupled receptor 1 family, which can affect the calcium flux (ii)OR may provide clues about bipolar disease[2,5]. (iii) CNV differentiation of olfactory receptors can explain the substantial olfactory deficits in patients with schizophrenia[8], a psychotic disorder related to bipolar disorder. |
| BD | *POU3F1* | 1p34.3 | 34.3-39.8 | rs16824514 | 7.55×10^-5^ | 1.06×10^-4^ | 3.83×10^-1^ | 1.16×10^-5^ | (i) with alias Oct6 (Octamer-binding transcription factor 6), playing a crucial role in neurodevelopment, shows potential relevance to schizophrenia[9], a psychotic symptom related to bipolar disorder. (ii) also can impose effects on calcium flux through binding to the promoter region of *PIK3C3*, a member of the phosphatidylinositide 3-kinase family, whose mutations may be involved in BD and SZ in a subset of patients[10]. |
| BD | *KCNQ5* | 6q13 | 70-75.9 | rs4991400 | 2.29×10^-4^ | 2.29×10^-4^ | 1.00 | 2.29×10^-4^ | (i)Recommended name is potassium voltage-gated channel subfamily KQT member 5, probably is important in the regulation of neuronal excitability, and may contribute to episodic disturbances of mood and behaves as well-recognized roles in other ion channelopathies[11].(ii)Similar gene, KCNC2, was found to be associated with bipolar disorder in WTCCC’s work[12].(iii) Recent study suggests that PIP5K2A, a schizophrenia-associated gene, works through failing to activate KCNQ2 and KCNQ5 channels[13]. |
| CAD | *PDPR* | 16q22.1 | 63.9-69.4 | rs2303200 | 1.64×10^-2^ | 3.93×10^-4^ | 8.98×10^-7^ | 8.98×10^-7^ | (i) Its expression is found to be correlated with copy number variation on both platforms[14]. (ii) Pyruvate dehydrogenase, which is regulated by *PDPR*, influences post-ischemic heart function[15]. |
| CAD | *AARS* | 16q22.1 | 63.9-69.4 | rs2303200 | 1.64×10^-2^ | 3.93×10^-4^ | 8.98×10^-7^ | 8.98×10^-7^ | *AARS* may have evolved as a compensatory mechanism so that cells can continue to synthesize protein in extreme conditions, such as myocardial ischemia[16]. |
| CAD | *CEACAMs* | 19q13.2 | 43.4-48.1 | rs2016070 | 1.51×10^-1^ | 9.54×10^-3^ | 1.39×10^-5^ | 1.39×10^-5^ | (i)The HIF-1/vEGF/CEACAM-1 pathway might be important for hypoxia-induced angiogenesis in the heart during hypoxic preconditioning[17].(ii)other cellular matrix protein have been proven to be associated with CAD[18]. |
| CD | *CEACAMs* | 19q13.2 | 43.4-48.1 | rs1015758 | 2.31×10^-1^ | 1.94×10^-3^ | 1.00×10^-4^ | 4.88×10^-5^ | localized to the apical glycocalyx of normal colonic epithelium with functioning as receptors of bacteria^[19,20]^, implied multiple roles in pathogenesis of Crohn’s disease[21,22] |
| HT | *TNNI3K* | 1p31.1 | 69.5-84.6 | rs596204 | 2.85×10^-5^ | 2.85×10^-5^ | 1.00 | 2.85×10^-5^ | (i) Highly expressed in both adult and fetal heart, May play a role in cardiac physiology. (ii) Its substrate, Cardiac ankyrin repeat protein, is a novel marker of cardiac hypertrophy[23], a related syndrome of hypertension. |
| HT | *CAMK4* | 5q22.1 | 109.6-111.5 | rs152875 | 5.08×10^-5^ | 2.65×10^-5^ | 5.20×10^-1^ | 8.98×10^-5^ | (i) Calcium/calmodulin pathway involves in vascular responsiveness to vasoconstrictors in portal hypertension[24]. (ii) The pathway of *CAMK4* involves in the regulation of vascular smooth muscle cell migration[25], a key event in the pathogenesis of many vascular diseases such as hypertension[26]. |
| HT | *STARD4* | 5q22.1 | 109.6-111.5 | rs152875 | 5.08×10^-5^ | 2.65×10^-5^ | 5.20×10^-1^ | 8.98×10^-5^ | (i) May be involved in the intracellular transport of sterols or other lipids, which have implied roles in cardiovascular disease[27]. (ii) Dyslipidemia, including elevated plasma triglycerides, low HDL cholesterol, and increased levels of atherogenic LDL cholesterol particles, is a characterization of Hypertension[28]. |
| HT | *NRAP* | 10q25.3 | 114.9-118.7 | rs2419854 | 1.34×10^-4^ | 1.34×10^-4^ | 1.00 | 1.69×10^-4^ | (i) Involved in anchoring the terminal actin filaments in the myofibril to the membrane and in transmitting tension from the myofibrils to the extracellular matrix, and may be an important stress-strain sensors embedded in cardiac cytoskeleton[29]. (ii) Expression differentiation was found in in pressure overload-induced cardiac hypertrophy in mice[30]. |
| HT | *CASP7* | 10q25.3 | 114.9-118.7 | rs2419854 | 1.34×10^-4^ | 1.34×10^-4^ | 1.00 | 1.69×10^-4^ | (i) Pro-apoptotic phenotype was evidenced in skeletal muscle of hypertensive rats[31]. (ii)A primary role of cytochrome c release and apoptosis was found in the pathogenesis of hypertensive nephrosclerosis in S rats[32]. (iii) Combination of a bradykinin antagonist and a caspase inhibitor (Z-Asp-2,6-dichlorobenzoyl-  oxymethylketone, Z-Asp) prevent severe pulmonary hypertension in a chronically hypoxic rat model[33]. |
| HT | *HABP2* | 10q25.3 | 114.9-118.7 | rs2419854 | 1.34×10^-4^ | 1.34×10^-4^ | 1.00 | 1.69×10^-4^ | (i) with recommended name, hyaluronan binding protein 2, converts the inactive single chain urinary plasminogen activator (pro-urokinase) to the active two chain form, involving in the fibrin clot. (ii) Its binding protein, hyaluronan, increased in idiopathic pulmonary arterial hypertension[34]. |
| HT | *VPS37C* | 11q12.2 | 59.8-61.5 | rs175126 | 2.26×10^-3^ | 2.26×10^-3^ | 1.00 | 5.20×10^-5^ | (i) Vacuolar protein sorting-associated protein 37C, is a component of the ESCRT-I complex, a regulator of vesicular trafficking process. (ii) Vesicular trafficking pathway is important for the vascular function[35], and is associated with  abnormalities of membrane function in hypertension[28]. |
| RA | *CHRNA7* | 15q13.3 | 29-31.4 | rs2926504 | 8.46×10^-7^ | 1.07×10^-5^ | 4.11×10^-1^ | 8.46×10^-7^ | Acetylcholine, the principal neurotransmitter of the vagal nerve, can controls immune cell functions via neuronal acetylcholine receptor subunit alpha-7, which make *CHRNA7* as a pharmacological target for inflammation[36]. |
| T1D | *CASP9* | 1p36.13 | 15.6-20.2 | rs6429757 | 8.75×10^-6^ | 6.20×10^-6^ | 1.00 | 8.75×10^-6^ | proved to participate in immune attack in a murine model for cell therapy-induced type I diabetes[37]. |
| T1D | *RSC1A1* | 1p36.13 | 15.6-20.2 | rs6429757 | 8.75×10^-6^ | 6.20×10^-6^ | 1.00 | 8.75×10^-6^ | The human gene *RSC1A1* encodes a 67-kDa protein named RS1 that mediates transcriptional and post-transcriptional regulation of the Na+-d-glucose cotransporter SGLT1,which is associated with diabetic nephropathy[38]. |
| T1D | *DAD1* | 14q11.2 | 19-24.3 | rs10873018 | 1.70×10^-7^ | 1.20×10^-7^ | 1.00 | 1.70×10^-7^ | With alternative name, defender against cell death, loss of which triggers apoptosis, members of whose pathway show close relationship with autoimmune disease[37,39]. |
| T2D | *CEACAMs* | 19q13.2 | 43.4-48.1 | rs2016070 | 5.79×10^-5^ | 3.64×10^-6^ | 4.44×10^-3^ | 5.79×10^-5^ | Inactivation of *CEACAM1* in L-SACC1 mice by a dominant-negative transgene in liver impairs insulin clearance and increases serum-free fatty acid (FFA) levels, resulting in insulin resistance[40,41,42]. |

**References**

1. Mudge J, Miller NA, Khrebtukova I, Lindquist IE, May GD, et al. (2008) Genomic convergence analysis of schizophrenia: mRNA sequencing reveals altered synaptic vesicular transport in post-mortem cerebellum. PLoS One 3: e3625.

2. Carter CJ (2007) Multiple genes and factors associated with bipolar disorder converge on growth factor and stress activated kinase pathways controlling translation initiation: implications for oligodendrocyte viability. Neurochem Int 50: 461-490.

3. Ooms LM, Horan KA, Rahman P, Seaton G, Gurung R, et al. (2009) The role of the inositol polyphosphate 5-phosphatases in cellular function and human disease. Biochem J 419: 29-49.

4. Craddock N, O'Donovan MC, Owen MJ (2005) The genetics of schizophrenia and bipolar disorder: dissecting psychosis. J Med Genet 42: 193-204.

5. Hahn CG, Gomez G, Restrepo D, Friedman E, Josiassen R, et al. (2005) Aberrant intracellular calcium signaling in olfactory neurons from patients with bipolar disorder. Am J Psychiatry 162: 616-618.

6. Porteous D (2008) Genetic causality in schizophrenia and bipolar disorder: out with the old and in with the new. Curr Opin Genet Dev 18: 229-234.

7. Umbach JA, Cordeiro ML, Gundersen CB (2004) Lithium regulates the expression of dense core vesicle proteins. Clinical Neuroscience Research 4: 253-261.

8. Moberg PJ, Agrin R, Gur RE, Gur RC, Turetsky BI, et al. (1999) Olfactory dysfunction in schizophrenia: a qualitative and quantitative review. Neuropsychopharmacology 21: 325-340.

9. Ilia M (2004) Oct-6 transcription factor. Int Rev Neurobiol 59: 471-489.

10. Stopkova P, Saito T, Papolos DF, Vevera J, Paclt I, et al. (2004) Identification of PIK3C3 promoter variant associated with bipolar disorder and schizophrenia. Biol Psychiatry 55: 981-988.

11. Graves TD, Hanna MG (2005) Neurological channelopathies. Postgrad Med J 81: 20-32.

12. (2007) Genome-wide association study of 14,000 cases of seven common diseases and 3,000 shared controls. Nature 447: 661-678.

13. Fedorenko O, Strutz-Seebohm N, Henrion U, Ureche ON, Lang F, et al. (2008) A schizophrenia-linked mutation in PIP5K2A fails to activate neuronal M channels. Psychopharmacology (Berl) 199: 47-54.

14. McCarroll SA, Altshuler DM (2007) Copy-number variation and association studies of human disease. Nat Genet 39: S37-42.

15. Lewandowski ED, White LT (1995) Pyruvate dehydrogenase influences postischemic heart function. Circulation 91: 2071-2079.

16. Rodovicius H (2003) [Seasonal differences in activity of tRNA and aminoacyl-tRNA synthetases of rabbit liver in myocardial ischemia]. Medicina (Kaunas) 39: 62-67.

17. Chen WJ, Chen HW, Yu SL, Huang CH, Wang TD, et al. (2005) Gene expression profiles in hypoxic preconditioning using cDNA microarray analysis: altered expression of an angiogenic factor, carcinoembryonic antigen-related cell adhesion molecule 1. Shock 24: 124-131.

18. Watkins H, Farrall M (2006) Genetic susceptibility to coronary artery disease: from promise to progress. Nat Rev Genet 7: 163-173.

19. Kuespert K, Pils S, Hauck CR (2006) CEACAMs: their role in physiology and pathophysiology. Curr Opin Cell Biol 18: 565-571.

20. Hauck CR, Agerer F, Muenzner P, Schmitter T (2006) Cellular adhesion molecules as targets for bacterial infection. Eur J Cell Biol 85: 235-242.

21. Servin AL (2005) Pathogenesis of Afa/Dr diffusely adhering Escherichia coli. Clin Microbiol Rev 18: 264-292.

22. Barnich N, Carvalho FA, Glasser AL, Darcha C, Jantscheff P, et al. (2007) CEACAM6 acts as a receptor for adherent-invasive E. coli, supporting ileal mucosa colonization in Crohn disease. J Clin Invest 117: 1566-1574.

23. Aihara Y, Kurabayashi M, Saito Y, Ohyama Y, Tanaka T, et al. (2000) Cardiac ankyrin repeat protein is a novel marker of cardiac hypertrophy: role of M-CAT element within the promoter. Hypertension 36: 48-53.

24. Gadano AC, Sogni P, Yang S, Cailmail S, Moreau R, et al. (1997) Endothelial calcium-calmodulin dependent nitric oxide synthase in the in vitro vascular hyporeactivity of portal hypertensive rats. J Hepatol 26: 678-686.

25. Pauly RR, Bilato C, Sollott SJ, Monticone R, Kelly PT, et al. (1995) Role of calcium/calmodulin-dependent protein kinase II in the regulation of vascular smooth muscle cell migration. Circulation 91: 1107-1115.

26. Tada T, Nawata J, Wang H, Onoue N, Zhulanqiqige D, et al. (2008) Enhanced pulsatile pressure accelerates vascular smooth muscle migration: implications for atherogenesis of hypertension. Cardiovasc Res 80: 346-353.

27. Callera GE, Montezano AC, Yogi A, Tostes RC, Touyz RM (2007) Vascular signaling through cholesterol-rich domains: implications in hypertension. Curr Opin Nephrol Hypertens 16: 90-104.

28. Zicha J, Kunes J, Devynck MA (1999) Abnormalities of membrane function and lipid metabolism in hypertension: a review. Am J Hypertens 12: 315-331.

29. Hoshijima M (2006) Mechanical stress-strain sensors embedded in cardiac cytoskeleton: Z disk, titin, and associated structures. Am J Physiol Heart Circ Physiol 290: H1313-1325.

30. van den Bosch BJ, Lindsey PJ, van den Burg CM, van der Vlies SA, Lips DJ, et al. (2006) Early and transient gene expression changes in pressure overload-induced cardiac hypertrophy in mice. Genomics 88: 480-488.

31. Quadrilatero J, Rush JW (2008) Evidence for a pro-apoptotic phenotype in skeletal muscle of hypertensive rats. Biochem Biophys Res Commun 368: 168-174.

32. Ying WZ, Sanders PW (2001) Cytochrome c mediates apoptosis in hypertensive nephrosclerosis in Dahl/Rapp rats. Kidney Int 59: 662-672.

33. Taraseviciene-Stewart L, Gera L, Hirth P, Voelkel NF, Tuder RM, et al. (2002) A bradykinin antagonist and a caspase inhibitor prevent severe pulmonary hypertension in a rat model. Can J Physiol Pharmacol 80: 269-274.

34. Papakonstantinou E, Kouri FM, Karakiulakis G, Klagas I, Eickelberg O (2008) Increased hyaluronic acid content in idiopathic pulmonary arterial hypertension. Eur Respir J 32: 1504-1512.

35. Mukherjee S, Tessema M, Wandinger-Ness A (2006) Vesicular trafficking of tyrosine kinase receptors and associated proteins in the regulation of signaling and vascular function. Circ Res 98: 743-756.

36. de Jonge WJ, Ulloa L (2007) The alpha7 nicotinic acetylcholine receptor as a pharmacological target for inflammation. Br J Pharmacol 151: 915-929.

37. de Witte MA, Jorritsma A, Swart E, Straathof KC, de Punder K, et al. (2008) An inducible caspase 9 safety switch can halt cell therapy-induced autoimmune disease. J Immunol 180: 6365-6373.

38. Hodgkinson AD, Millward BA, Demaine AG (2001) Polymorphisms of the glucose transporter (GLUT1) gene are associated with diabetic nephropathy. Kidney Int 59: 985-989.

39. Vaux DL, Flavell RA (2000) Apoptosis genes and autoimmunity. Current Opinion in Immunology 12: 719-724.

40. Dai T, Abou-Rjaily GA, Al-Share QY, Yang Y, Fernstrom MA, et al. (2004) Interaction between altered insulin and lipid metabolism in CEACAM1-inactive transgenic mice. J Biol Chem 279: 45155-45161.

41. DeAngelis AM, Heinrich G, Dai T, Bowman TA, Patel PR, et al. (2008) Carcinoembryonic antigen-related cell adhesion molecule 1: a link between insulin and lipid metabolism. Diabetes 57: 2296-2303.

42. Park SY, Cho YR, Kim HJ, Hong EG, Higashimori T, et al. (2006) Mechanism of glucose intolerance in mice with dominant negative mutation of CEACAM1. Am J Physiol Endocrinol Metab 291: E517-524.
