# Supplementary material for: Novel Association Strategy with Copy Number Variation for Identifying New Risk Loci of Human Diseases
Source: PLoS One. 2010 Aug 20;5(8):e12185. doi: 10.1371/journal.pone.0012185 (PMC2924882; doi:10.1371/journal.pone.0012185)
Supplement: Text S1 — Supporting methods of multiple testing for trend. (0.08 MB DOC) [file pone.0012185.s008.doc]

**Supporting methods of multiple trend testing**

Trend effect model can interpret the quantitative phenotype caused by the CNV with altered gene expression. Here, we applied three trend testing on the contingency table summarized from copy number for specific SNP site. The methods were modified from SNP-association analysis[1] to CNV-association with new feartures.

| $Z$ | $cn\leq1$ | $cn=2$ | $cn\geq3$ |
| --- | --- | --- | --- |
| cases | $n_{11}$ | $n_{12}$ | $n_{13}$ |
| Controls | $n_{21}$ | $n_{22}$ | $n_{23}$ |

Pearson Chi-Squared testing is a non-parametric testing with no presumed trend model, and the null hypothesis is that proportions in cases and controls are the same. The calculations for $\chi^{2}$ are as the same in the $2\times2$ table for SNP site-based testing. The *P* value was from the $\chi^{2}$ distribution with 2 degree freedom. If any of the $e_{ij}$is less than 5, a Fisher’s exact test that is as the same in the SNP site-based testing was applied instead.

Armitage trend testing is for ordinary effect of copy number on disease. Let $s_{1}=1$, $s_{2}=2$, and $s_{3}=3$ be for the weight of $cn\leq1$, $cn=2$, $cn\geq3$, and $\bar{s}=\frac{\sum\left(n_{1j}+n_{2j}\right)s_{j}}{N}$,$p_{case}=\frac{\sum n_{1j}}{N}$,$p_{jcase}=\frac{n_{1j}}{n_{1j}+n_{2j}}$,. When the any element $n_{ij}$ is equal to zero, 0.5 was added to every element to eliminate declination. So the prediction equation under ordinary least-squares fit is

$$\hat{p}_{casej}=p_{casej}+b\left(s_{j}-\bar{s}\right)()$$

The statistic for the Armitage trend testing is

$$z^{2}=\frac{b^{2}}{p_{case}\left(1-p_{case}\right)}\sum\left(n_{1j}+n_{2j}\right)\left(s_{j}-\bar{s}\right)()\sum^{2}$$

Which is in a asymptotically $\chi^{2}$ distribution with 1 degree of freedom. The *P* value for the Armitage trend testing can be calculated from this distribution.

Armitage exact testing is for exact probability for the trend, and it used a trend score $T^{\left(m\right)}=\sum n_{1j}^{\left(m\right)}s_{1j}$ to measure trend for the permuted contingency table, in which .

The exact permutation *P* value was evaluated as

$$p_{armexact}=\sum_{\left|T^{\left(m\right)}\right|\geq\left|T_{observed}\right|} p^{\left(m\right)}$$

where $p^{\left(m\right)}$ was calculated as the same in the Fisher exact test that is in the SNP site -based testing.

**References**
